# One Health in Action: Operational Aspects of an Integrated Surveillance System for Zoonoses in Western Kenya

**DOI:** 10.3389/fvets.2019.00252

**Published:** 2019-07-31

**Authors:** Laura C. Falzon, Lorren Alumasa, Fredrick Amanya, Erastus Kang'ethe, Samuel Kariuki, Kelvin Momanyi, Patrick Muinde, Maurice K. Murungi, Samuel M. Njoroge, Allan Ogendo, Joseph Ogola, Jonathan Rushton, Mark E. J. Woolhouse, Eric M. Fèvre

**Affiliations:** ^1^Institute of Infection and Global Health, University of Liverpool, Liverpool, United Kingdom; ^2^International Livestock Research Institute, Nairobi, Kenya; ^3^Faculty of Veterinary Medicine, University of Nairobi, Nairobi, Kenya; ^4^Kenya Medical Research Institute, Nairobi, Kenya; ^5^Veterinary Department, Busia County Government, Busia, Kenya; ^6^Veterinary Department, Bungoma County Government, Bungoma, Kenya; ^7^Centre for Immunity, Infection and Evolution and Usher Institute of Population Health Sciences and Informatics, University of Edinburgh, Edinburgh, United Kingdom

**Keywords:** zoonoses, public health, veterinary public health, livestock markets, slaughterhouses, hospital patients, Kenya, surveillance

## Abstract

Surveillance of diseases in Kenya and elsewhere in East Africa is currently carried out by both human and animal health sectors. However, a recent evaluation highlighted the lack of integration between these sectors, leading to disease under-reporting and inefficiencies. This project aimed to develop an integrated and cost-effective surveillance and reporting system for 15 zoonotic diseases piloted in the counties of Bungoma, Busia, and Kakamega in western Kenya. Specifically, in this paper we describe the operational aspects of such a surveillance system. Interviews were carried out with key informants, and this was followed by field visits to identify sentinel sites and liaise with relevant stakeholders. Based on this information, a sampling strategy comprising 12 sentinel sites, 4 in each county, was developed. Each sentinel site comprised of a livestock market, 1–2 neighboring slaughter houses/slabs, and a hospital in the vicinity; each of the 12 sites, comprising 12 × 3 = 36 sampling locations, was visited every 4 weeks for 20 cycles. At each site, animal or patient sampling included a clinical examination and collection of blood, feces, and nasal swabs; in slaughtered animals, mesenteric lymph nodes, hydatid cysts, and flukes were also collected. At the end of each field visit, data on staff involved and challenges encountered were recorded, while biological samples were processed and tested for 15 zoonotic diseases in the field laboratory in Busia, Kenya. Public engagement sessions were held at each sentinel site to share preliminary results and provide feedback to both stakeholders and study participants. A livestock market visit lasted just over 3 h, and the most common challenge was the frequent refusals of animal owners to participate in the study. At the slaughterhouses, visits lasted just under 4 h, and challenges included poorly engaged meat inspectors or slaughter processes that were too quick for sampling. Finally, the hospital visits lasted around 4 h, and the most frequent challenges included low patients turn-out, frequent staff turn-over leading to poor institutional memory, and difficulty in obtaining patient stool samples. Our experiences have highlighted the importance of engaging with local stakeholders in the field, while also providing timely feedback through public engagement sessions, to ensure on-going compliance.

## Introduction

Surveillance activities involve the systematic collection, analysis, and evaluation of health-related data from a population of interest (1). These data, in turn, can be used to enhance disease preparedness, improve resource allocation, and guide disease intervention strategies (2–5).

The design of the surveillance components is guided by the specific purpose for which the surveillance system is built, and the characteristics of the disease(s) of interest. Zoonotic diseases are naturally transmitted between vertebrate animals and humans (6, 7). Surveillance of zoonoses is therefore suited for collaborative methods that allow for integration of information on their presence and distribution in all affected hosts (8, 9). Such an integrated approach will provide more accurate estimates of the disease burden in the multiple populations. Integration may also enhance the surveillance system's timeliness as the animal population may serve as an early warning for the likelihood of presence of disease in the human population. Moreover, collaborations between multiple sectors could lead to cost savings through sharing of resources and earlier disease detection, thus creating more efficient and effective systems (9–11). Finally, integrated surveillance systems may also lead to intangible benefits in terms of enhanced intellectual and social capacity (12).

In Kenya, surveillance is currently carried out by departments within the Ministry of Health, the Ministry of Agriculture Livestock and Fisheries, and the Kenya Wildlife Services, and each sector has its own priorities and reporting systems. These systems involve different data collection systems, different management processes, and different budgets. Surveillance in Kenya is further complicated by the devolved nature of the government, resulting in certain types of surveillance no longer being a nationally organized function (13, 14). However, a recent evaluation of the systems currently in place revealed various gaps, particularly with regards to lack of infrastructure in the animal health sector, and an overall under-reporting of zoonotic diseases (15). Most information on notifiable zoonotic diseases in Kenya is currently based on reports from meat inspectors in slaughterhouses, and from field veterinarians and animal health assistants present in livestock markets or who are called to visit farms in case of disease. However, farmers may sometimes be unwilling to report a disease due to lack of compensation or feedback, fear of castigation, or if they cannot afford the necessary services (e.g., treatment, vaccination). Furthermore, many diseases are under- or misdiagnosed due to staff shortages and lack of diagnostic capacity.

Despite the recognition of benefits that can be accrued through integration, all those involved in the evaluation of the Kenyan surveillance systems also highlighted the lack of integration between the different sectors (15). This lack of integration is often due to challenges in data sharing, which may itself be a consequence of technological barriers, lack of trained personnel, or lack of trust between the sectors involved (10, 16). Moreover, different sectors collect data for different purposes and often use disparate formats and terminology, making it harder to then collate and interpret (9, 11).

The overall objective of this study was to improve the epidemiological understanding of fifteen zoonoses in western Kenya through the development and implementation of a surveillance system integrated across both human and animal sectors. Specifically, in this paper we describe the operational aspects of the surveillance system, including field implementation and sampling activities, and discuss logistics and challenges encountered during such activities.

## Methods

### Surveillance Purpose

The objective of the surveillance system was to improve the epidemiological understanding and provide an estimate of the amount and distribution of 15 zoonoses in western Kenya. Specifically, the 15 zoonotic pathogens of interest included: *E. coli, Salmonella* spp., *Campylobacter* spp., *Staphylococcus aureus, Brucella* spp., *Coxiella burnetti, Rift Valley Fever virus, Leptospira* spp., *Toxoplasma* spp., *Taenia solium, Mycobacterium* spp., *Typanosoma* spp., *Bacillus anthracis, Echinococcus* spp., and *Fasciola* spp. The selection of the diseases of interest was based on several factors. Firstly, the need to prioritize surveillance for neglected and zoonotic infections for which few public sector data are collected in animal or human health (17, 18); indeed, most of these diseases do not appear in the list of reportable diseases in the national Health Management Information System (19). Secondly, recent research work in the study area which demonstrated an underestimated burden of Q-fever, bovine tuberculosis, Rift Valley Fever, *Campylobacter* spp., *Stapyhylococcus aureus*, and cysticercosis (20–24), and an overestimated burden of brucellosis (25). Lastly, a recent prioritization exercise for zoonotic diseases in Kenya (26), which includes five of the eight notifiable zoonotic diseases that affect our population of interest (i.e., anthrax, Rift Valley Fever, brucellosis, tuberculosis, and trypanosomiasis) (27, 28). The population of interest were (i) cattle, small ruminants and pigs at livestock markets and slaughterhouses/slabs, and (ii) patients at private and public sector hospitals, in the counties of Busia, Bungoma, and Kakamega in western Kenya.

### Sampling Design

The western Kenya region was chosen for this study as it represents the larger Lake Victoria basin ecosystem, a region with, concurrently, the highest rural human and livestock population densities in East Africa, operating in a mixed smallholder livestock production system. Furthermore, husbandry practices in this region are changing as production moves from largely subsistence to increasing intensification and market orientation, with consequent impacts on disease transmission (24).

In July-August 2016, interviews were conducted with relevant stakeholders in the study area, including County directors of health, County and sub-County medical officers, County directors of veterinary services and sub-County veterinarians, and meat inspectors. During the interviews, questions were asked to better understand the health and livestock systems in the study area and the key stakeholders involved. Based on the information collected during these sessions, a sampling frame of all health facilities, livestock markets and slaughterhouses/slabs in the study area was developed (Table S1). This included 20 livestock markets, 17 slaughterhouses/slabs, and 12 hospitals in Bungoma county; 18 livestock markets, 43 slaughterhouses/slabs, and 12 hospitals in Busia county; and 15 livestock markets, 13 slaughterhouses/slabs, and 9 hospitals in Kakamega county.

**Figure 1:**
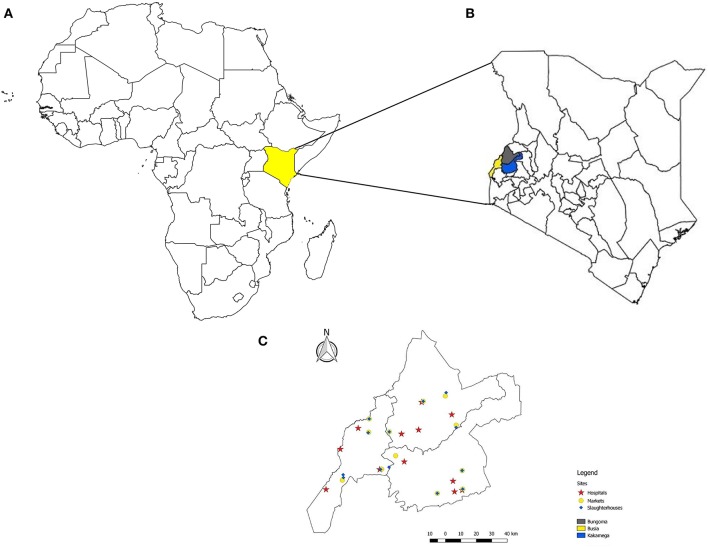
A map of the African continent highlighting the position of Kenya **(A)**; the counties of Busia, Bungoma, and Kakamega in western Kenya **(B)**, and the location of the sentinel sites, i.e., livestock markets, slaughterhouses and slabs, and hospitals, included in an integrated surveillance program for zoonotic diseases in western Kenya **(C)**.

In each county, 4 to 6 livestock markets, 4 to 13 slaughterhouse/slabs, and 4 to 5 hospitals were selected based on both epidemiological (i.e., catchment area and number of patients/animals at each site) and logistical (i.e., distance from the central Busia laboratory and livestock market day) factors. These sites were visited between September-December 2016 to better assess their suitability for sampling purposes and to sensitize local stakeholders about the planned surveillance work. Based on observations and feedback from stakeholders received during the visits, a final sampling scheme including 12 sentinel sites (4 in each County), and representing 20–27%, 9–31%, and 33–44% of the total livestock markets, slaughterhouses, and hospitals in each County, was devised (Figure 1). Each sentinel site comprised of a livestock market, 1–2 neighboring slaughterhouses/slabs, and a hospital in the vicinity. The final selection was based on the geographical location, size, and species present (for slaughterhouses) at each site, to ensure representativeness of the study area. Each sentinel site was visited once every 4 weeks for 20 cycles, and the sampling day was selected so as to coincide with the livestock market day.

### Animal and Patient Sampling

The team visiting the animal sites comprised six to eight veterinarians and animal health assistants, while the team visiting the hospital sites comprised two clinical officers. Team members were primarily project staff, but also included government officials seconded to the project and interns. The same teams visited the animal and human facilities in all 12 sites, and for the entire study duration. Each team departed from a central site (i.e., Busia town) and traveled to the sentinel sites using one of the project vehicles; travel time ranged between 30 min and 2 h each way.

During each site visit, up to 10 animals at the livestock market, up to 10 animals at the neighboring slaughterhouse/slab(s), and up to 10 patients at the selected health facility in the vicinity were sampled. Sample size per visit was determined based on both the total number of individuals that needed to be sampled over the entire study period, in turn based on expected frequencies of specific zoonotic diseases of interest in the study area, and on logistics (i.e., staff and time available).

At the livestock market, the market chairperson and/or market master were approached and informed of our visit. Subsequently, up to 10 animals (6–7 cattle and 3–4 small ruminants) were selected using systematic random sampling. This was done by roughly estimating the number of animals present in the market and, starting from one corner of the market, walking around the market and sampling every nth animal (e.g., if we estimated there to be a 100 animals in the market, we sampled every 10th animal). If the owner of the selected animal refused to participate, the next consenting owner was included. The selected animal was sampled regardless of whether it appeared healthy or sick.

For each selected animal, the owner was identified and the study explained. If consent was obtained, the animal was restrained and its biodata recorded. A rapid clinical examination was first performed, including girth measurement (in cm) taken behind the shoulders and recording of the rectal temperature (in °C). Biological samples were then collected; these included: blood samples from the jugular vein collected into 10 ml plain, heparinized, and EDTA tubes (BD) using an 18-gauge rubber-capped needle; nasal swabs using a sterile nasal swab with saline solution (MWE–E Transwab), and fecal samples collected per rectum (or from the ground if freshly deposited) and stored in 20 ml fecal pots (Greiner Bio-one). Any external parasites present were also collected and placed in plastic Bijoux tubes with 70% ethanol. Samples were then bar-coded and transported in cool-boxes with two ice packs, except for the heparinized blood tubes which were maintained at ambient temperature during transport.

Subsequently, a questionnaire was administered to the animal owner to collect information on where the animal came from and where it would be taken next. If the animal owner identified him/herself as a farmer (rather than a trader), questions on any relevant clinical history, including previous treatments and vaccinations (if the animal was born on their farm), and questions on other owned animals were also asked.

At the slaughterhouses, we liaised with the resident meat inspector and sampled all the animals brought for slaughter (if < 10 animals were slaughtered), or a sub-sample of these (if > 10 animals were slaughtered), on a given visit. If more than 10 animals were brought to the slaughterhouse, the first available 10 animals were sampled. This was done to allow the team sufficient time to complete sampling and travel back to the laboratory on time. Animals were sampled regardless of whether they appeared healthy or sick; only when down animals (e.g., with fractured limbs) were brought, and sampling would prolong their pain, did the team opt to not sample that animal on welfare grounds.

In addition to the ante-mortem sampling described for the livestock market above, a post-mortem inspection of each animal sampled at the slaughterhouse was performed. This included inspection of all organs, with specific focus on the liver, lungs, heart, and spleen. Any liver flukes present were placed in plastic Bijoux tubes with 70% ethanol, while hydatid or lingual cysts were excised and placed in Ziploc bags. Additionally, any condemnations made by the meat inspector were recorded. For slaughtered pigs, the mesenteric lymph nodes were also collected using carbon steel blades size 22 (Swann-Morton) and placed in plastic Bijoux tubes for bacterial cultures. Samples were transported to the laboratory as described above.

At the hospitals, the sampling team worked closely with resident clinical officers and lab technicians to ensure that sampling did not disrupt the normal hospital operations. Up to 10 patients who met the study inclusion criteria (i.e., presenting with one or more of the following clinical signs suggestive of a zoonotic disease: fever, anemia, jaundice, muscle pain, joint pain, headache, epileptic episodes, respiratory signs, skin lesions, diarrhea, vomiting, nausea, and abdominal cramps), and not having a lab-confirmed diagnosis of malaria and/or dengue, were considered for inclusion on a given visit.

If the patient accepted to participate in the study and consent/assent was obtained, a questionnaire on demographics, socio-economic status, dietary habits, and contact with animals was administered. The patient was then clinically examined by one of the team clinical officers; this included measurement of height and middle upper arm circumference (in cm), weight (in kg), and body temperature (in °C). Other parameters, including systolic and diastolic blood pressure, mucous membranes, and any painful areas, were also assessed. The team clinical officers then collected a nasal swab using a sterile nasal swab with saline solution (MWE–E Transwab), before sending the patient to the hospital laboratory where blood was collected into 5 ml serum tubes and 4 ml EDTA tubes using vacutainer butterfly needles gauge 21 and 23, and a butterfly needle adaptor. This was done to avoid pricking the patient twice. The patient was also given a fecal pot and asked to provide the team with a stool sample. All samples were barcoded and transported in a cool-box with two ice-packs.

All data were collected electronically on GPS-enabled hand-held devices using the Field Information Support Tool which was linked directly to a secure cloud server and a custom designed database managed by Kestrel Technology Group, LLC. These data were collected in parallel with the national system and, being a research activity, involved a much greater depth than normal livestock and hospital recording allows. Data analyses will be conducted separately from the national surveillance databases, and key results will be communicated to all relevant authorities to inform which key data components might need to be collected and integrated into the final system chosen for use by the Government of Kenya.

All data collection forms (Tables S2–S4), protocols (Table S5), and list of consumables (Table S6) used during livestock market, slaughterhouse, and hospital sampling are available as Supplementary Material.

### Evaluation of Field Visits

At the end of each field visit, an evaluation form (Table S7) was completed to record the duration of the visits (excluding travel time), the number of staff involved, the number of animals/patients sampled, challenges encountered, and an overall assessment of the visit, ranging from 1 (very difficult) to 5 (very easy). Since the same team members visited each sentinel site, assessment was based on how easy or hard it was to get the samples during a specific visit, compared to both previous visits at the same site and to visits at other sentinel sites.

### Laboratory Testing

Relevant biological samples were transported to the International Livestock Research Institute/Government of Kenya Directorate of Veterinary Services Zoonoses Laboratory and Research Facility in Busia, Kenya. This is an interdisciplinary facility where both animal and human samples are processed. All samples were processed within 4–6 h post-collection and either stored at −40°C or tested for the 15 zoonoses of interest (Table 1 and Tables S8–S15). Samples that tested positive, and any pathogens isolated from bacterial cultures, were stored for further identification and testing, including antimicrobial susceptibility assessment for *E. coli, Salmonella* spp., *Campylobacter* spp., and *Staphylococcus aureus* isolates. In addition to the tests described, fecal and blood samples were aliquoted and stored for future molecular and genetic work.

**Table 1:** Biological samples collected, laboratory tests performed, and pathogens investigated, during an integrated surveillance program for zoonoses in western Kenya.

**Biological sample**	**Laboratory test**	**Pathogen investigated**	**Protocol**
Feces	Bacterial culture and identification	*E. coli*	Table S8
		*Salmonella spp*.	Table S9
		*Campylobacter spp*.	Table S10
Mesenteric lymph nodes (pigs)	Bacterial culture and identification	*Salmonella spp*.	Table S9
		*Campylobacter spp*.	Table S10
Nasal swabs	Bacterial culture and identification	*Staphylococcus aureus*	Table S11
**Blood**			
– Serum	Rose Bengal Test	*Brucella spp*.	Table S12
	ELISA	*Coxiella burnetii* (Human anti-Q-Ab ELISA Kit [Biosource] and Ruminant CHECKIT Q Fever Ab Test [IDEXX])	As per manufacturer's instructions
		*Rift Valley Fever virus* (ID Screen Rift valley fever IgM capture ELISA kit [Innovative Diagnostics])	As per manufacturer's instructions
		*Leptospira spp*. (Porcine Leptospira Bratislava ELISA, Strip Kit, and Linnodee Bovine Leptospirosis Kit [Linnodee])	As per manufacturer's instructions
		*Toxoplasma spp*. (Anti-Toxoplasma gondii IgG Human ELISA Kit [Abcam] and Toxoplasma IgM ELISA [DRG International])	As per manufacturer's instructions
		*Taenia solium*	Table S13
– Heparinized blood	Bovigam	*Mycobacterium spp. (BOVIGAM TB Kit and BOVIGAM TEST KIT (Gamma interferon)[Prionics])*	As per manufacturer's instructions
– Whole blood	Microscopy (thick smears)	*Trypanosoma spp*.	Table S14
	Microscopy (thin smears)^*^	*Bacillus anthracis*	Table S15
External parasites	Stored for further testing		
Liver flukes			
Hydatid cysts			
Other cysts			

* *This test was only performed in case of a suspect animal or patient*.

### Public Engagement Sessions

Public engagement sessions were conducted at each sentinel site. These sessions took on different formats based on the site. At livestock markets, farmers, traders, trekkers and other people present were gathered (as one crowd or in small groups) and one of the field team members briefly presented the study and preliminary results for animals sampled at that site, while also highlighting comparisons with results from the slaughterhouse and health facility. In the slaughterhouse/slabs, the same information was shared with individuals or small groups of stakeholders. Finally, at the hospitals, the team members gave regular presentations to hospital staff during their ongoing Continued Medical Education (CME) sessions. For every session, an informative pamphlet briefly explaining the study and providing preliminary results for animals or patients sampled at that site was prepared and distributed during the public engagement sessions. Information emanating from this study was also continually disseminated to a wider audience via social media platforms (website blog entries at http://www.zoonotic-diseases.org/blog/ and Twitter at https://twitter.com/zoonoticdisease), as well as featured in the study quarterly newsletters.

### Ethical Approval

This study was approved by the Institutional Animal Care and Use Committee (IACUC Reference No. 2017-04) and the Institutional Research Ethics Committee (IREC Reference No. 2017-08) at the International Livestock Research Institute, review bodies approved by the Kenyan National Commission for Science, Technology and Innovation. Approval to conduct the work was also obtained from the Department of Veterinary Services and the Ministry of Health, and the relevant offices of these Ministries at devolved government level.

All recruited animal owners (in markets and slaughterhouses/slabs) and all interviewed patients in hospitals gave written, informed consent prior to their inclusion in the study.

## Results

### Sampling Design

Field sampling started in March 2017 and was completed in May 2019. Sampling was interrupted several times due to prolonged electioneering period, disease outbreaks leading to closure of markets and slaughterhouses, and nation-wide health workers' strikes.

### Animal and Patient Sampling

#### Livestock Markets

During the livestock market visits, 1,977 animals were sampled; these included 1,293 cows, 367 goats, and 317 sheep. The sampled cattle and goats were most commonly classified as either local (56% of cows and 77% of goats) or cross (26% of cows and 22% of goats) breeds, while the sampled sheep were most commonly classified as indigenous hair (49%) or fat-rumped (39%) breeds. Of the 1,977 sampled animals, 1,192 (60%) were female, and the mean age of sampled animals was 28.5 months (ranging from 3 to 154 months); this was slightly higher in cows and sheep (mean age of 30 months), and lower in goats (mean age of 22 months). External parasites, mostly ticks, were identified in 723 (37%) animals, primarily cows (*n* = 667).

Most of the sampled animals belonged to traders (*n* = 1,514; 77%) or farmers (*n* = 418; 21%); 26 participants identified themselves as both traders and farmers. The majority of the owners (*n* = 1,726; 87%) were selling the sampled animal, while fewer (*n* = 251; 13%) had just bought the sampled animal.

#### Slaughterhouses and Slabs

A total of 1,509 animals were sampled in slaughterhouses and slabs; these included 885 cows, 296 pigs, 229 goats, and 99 sheep. The breed distribution was similar to that observed in the livestock markets, whereby cows, pigs, and goats were most commonly classified as either local (64, 41, and 92%, respectively) or cross (20, 56, and 5%, respectively) breeds, while sheep were classified as either fat-rumped (59%) or indigenous hair (33%) breeds. Of the sampled animals, 57% (854) were female, and the mean age was 35 months (ranging from 3 to 120 months); this was higher in cows (mean age of 44 months), and lower in pigs, goats, and sheep (mean age of 16, 27, and 32 months, respectively). Most of the sampled animals were brought to the slaughterhouse by a butcher (*n* = 1,367; 93%), with fewer animals brought by traders (*n* = 85; 6%) or farmers (*n* = 17; 1%).

External parasites were identified in 40% (*n* = 597) of the sampled animals; these were primarily ticks (*n* = 473) in ruminants, and lice (*n* = 98) in pigs; 11 pigs had both ticks and lice. During post-mortem inspection, liver flukes were identified in 330 (22%) animals, mostly cows (*n* = 318). Additionally, hydatid and lingual cysts were identified in 67 and 3 animals, respectively.

#### Hospitals

A total of 944 hospital patients were sampled; of these, 286 (30%), 267 (28%), and 391 (42%) were patients at Referral, Missionary, and sub-County hospitals, respectively. More than half of the sampled patients were female (*n* = 662; 70%), and most patients belonged to the Luhya tribe (*n* = 732; 78%). The mean patient age was 33 years (ranging from 2 to 88 years), though this was slightly higher in females (mean age of 35 years), and slightly lower in males (mean age of 31 years).

### Field Visit Evaluation

Evaluation of the field visits started in November 2017, and data for 15 cycles (i.e., 6th−20th cycle) were collected; this included evaluation of 165, 167, and 165 livestock market, slaughterhouse/slab and hospital visits, respectively (Table 2).

**Table 2:** An evaluation of the livestock market, slaughterhouse and hospital visits during an integrated surveillance program for zoonoses in western Kenya.

**Site**	**No. of evaluated visits**	**Mean duration of visit (hours)**	**Mean no. of core team members involved**	**Mean no. of other staff engaged**	**Mean no. of animals or patients sampled per visit**	**No. of visits with incomplete data**	Proportion of sampled animals or patients with incomplete data^*^	**Median assessment of field visit**	**Main challenges encountered**
Livestock markets	165	3.15 (1–5.5)	3 (1–5)	2 (1–4)	9.9 (6–10)	93 (56%)	0.18 (0.1–0.8)	3 (1–5)	1. Refusal to participate in study 2. Inadequate restraining facilities
Slaughterhouses	167^**^	3.75 (1.5–6)	3 (1–6)	1 (0–2)	7.7 (0–12)				1. Poor engagement of stakeholders 2. Inadequate restraining facilities 3. Slaughter process too quick for sampling
– Ruminant slaughterhouses/slabs	140	–	–	–	7.4 (1–10)	71 (51%)	0.28 (0.1–1)	3 (1–4)	
– Pig slabs	75	–	–	–	3.4 (0–10)	19 (25%)	0.47 (0.11–1.0)	4 (1–5)	
Hospitals	165	4.3 (1–6.5)	2	3 (0–4)	5 (0–10)	109 (67%)	0.36 (0.1–1)	3 (1–4)	1. Poor engagement of stakeholders 2. Frequent staff turnover at some facilities 3. Low patient turn-out

* *Proportion (and range) of patients with missing data on those visits with incomplete data*.

** *Of the 167 slaughterhouse visits evaluated, 119 comprised 1 site while 48 comprised 2 site*.

#### Livestock Markets

A livestock market visit lasted a mean of 3.15 h (ranging from 1 to 5.5 h) and involved 3 core team members. Additionally, a mean of 2 (range from 1 to 4) stakeholders were engaged for the duration of each visit; this included the market master or chairperson, and another one or two persons to assist with recruitment of participants and animal restraint. A mean of 9.9 animals per visit were sampled; fewer than 10 animals were sampled on those occasions when many owners refused to participate, thus delaying sampling.

During 56% (*n* = 93) of the evaluated livestock market visits, ~ 18% (ranging from 10 to 80%) of the sampled animals had incomplete data. Most commonly missed data included fecal samples, blood samples, and nasal swabs; reasons for missing data included an empty rectum, the animal could not be adequately restrained, or the animal owner did not allow for that sample to be taken.

**Figure 2:**
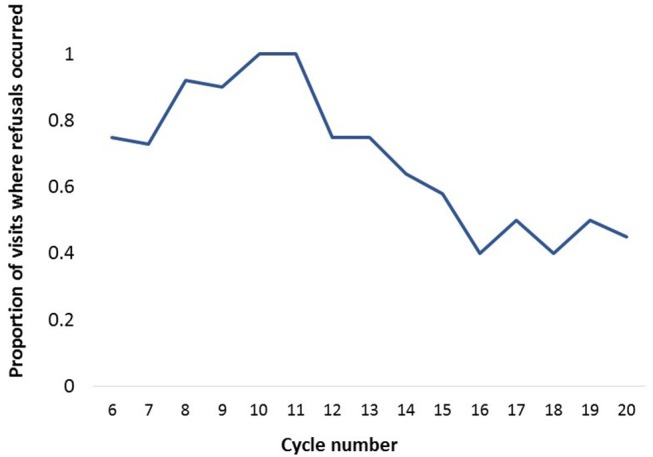
The proportion of livestock market visits where refusals occurred during fifteen (i.e., 6th−20th) 4-week cycles of an integrated surveillance program for zoonotic diseases in western Kenya.

The median assessment score of livestock market visits was 3 (ranging from 1 to 5), and this was based on cooperation of stakeholders and participants during the visit, and restraining facilities present. The primary challenge during livestock market visits was owners refusing to participate in the study, either because they were eager to sell their animals quickly or because they feared that the animal would become weak after sampling. Others refused to participate because they thought that if their animal was seen being sampled, it would be construed by the potential buyers to be unhealthy. Some owners expected payment; others associated the sampling with movement restrictions imposed during disease outbreaks, while others simply refused without giving any reason. However, we observed that the proportion of visits where refusals occurred decreased over time, going from 73 to 100% in the first 5 evaluated (i.e., 6th−10th) cycles, to 40–50% in the last 5 (i.e., 16th−20th) cycles (Figure 2).

#### Slaughterhouses and Slabs

Of the 167 slaughterhouse visits evaluated, 119 visits (71%) comprised 1 site (i.e., only a ruminant or pig slaughterhouse/slab) while 48 (29%) comprised 2 sites (both a ruminant and pig slab). Each visit lasted a mean of 3.75 h (ranging from 1.5 to 6 h), and involved 3 core team members. Additionally, one or two stakeholders were also engaged for the duration of each visit; this included the meat inspector to facilitate the sampling process, and occasionally a slaughterhouse worker to assist with animal restraint. A mean of 7.7 animals per visit were sampled; on those visits when the target sample size of 10 was not reached, this was either because fewer than 10 animals were brought for slaughter on the sampling day, or because the slaughter process was too quick and did not allow for sampling of all animals present.

A ruminant slaughterhouse or slab was included in 140 (84%) of the 167 visits, and the mean number of ruminants sampled per visit was 7.4 (ranging from 1 to 10). During 51% (*n* = 71) of the ruminant slab visits, 28% (ranging from 10 to 100%) of the sampled ruminants had incomplete data. Fecal samples were the most commonly missed, followed by post-mortem and clinical data (e.g., temperature recording, lymph node palpation), and this was primarily because of empty rectums, the animal could not be adequately restrained, or the slaughter process was too quick for sampling purposes. The median assessment score of the visits to the ruminant slaughterhouses was 3 (ranging from 1 to 4); this score was influenced by the interactions with the meat inspector, slaughterhouse workers and other butchers and traders present; the restraining facilities; and the dynamics of the slaughter process.

Pig slabs were included in 45% (*n* = 75) of the visits, and the mean number of pigs sampled per visit was 3.4 (ranging from 0, where either the slab did not open or the pig had already been slaughtered by the time the team arrived, to 10). Incomplete data were obtained during 19 (25%) of these visits; mesenteric lymph nodes were the most frequently missed data, either because the intestines were discarded or because the animal was not slaughtered on the day. The median assessment score of pig slab visits was 4 (ranging from 1 to 5), and the factors behind this assessment were similar to those cited above for ruminant slaughterhouse visits.

#### Hospitals

Each hospital visit lasted a mean of 4.3 h (ranging from 1 to 6.5 h), and involved 2 core team members and an additional 2 to 3 other staff. The latter included clinical officers to help with identification and recruitment of patients, and lab technologists to assist with sample collection. A mean number of 5 patients were sampled per visit, though this ranged from 0 to 10. Fewer than 10 patients were sampled on 157 (96%) visits, and this was mostly because there were not enough patients who met the inclusion criteria at the facility, or due to logistical issues (e.g., changes in staff or other administrative issues) which delayed sampling.

At least one patient refused to be sampled during 46 of the 165 (28%) visits, and the number of patients who refused on these occasions ranged between 1 and 3. Most of these patients refused either because they were in a hurry, or they simply were not interested in the study; a few patients wanted to first get their hospital test results before agreeing to further sampling. Incomplete data were obtained during 109 of the 165 (66%) visits, and on these occasions 36% (ranging between 10 to 100%) of the sampled patients had incomplete data. The most frequently missing data were stool and blood samples, either because the patient could not produce stool, or did not proceed to the lab for blood and stool sample collection.

The median assessment score of hospital visits was 3 (ranging from 1 to 4), and this was based on the willingness of patients to participate in the study, hospital staff cooperation and engagement, and patient turn-out.

### Public Engagement Sessions

**Figure 3:**
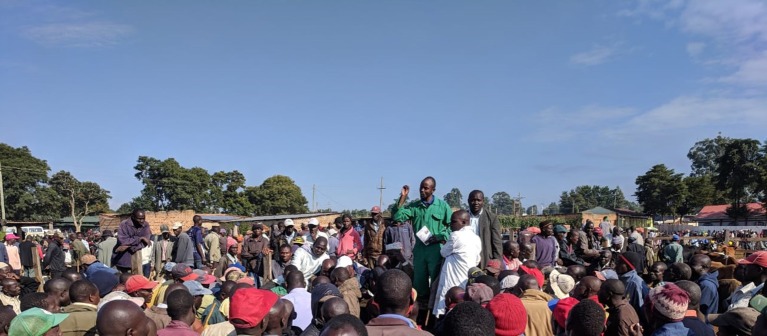
Public engagement sessions consisting of public addresses and distribution of pamphlets at sentinel livestock markets. Consent has been obtained from all participants identifiable in the photos.

**Figure 4:**
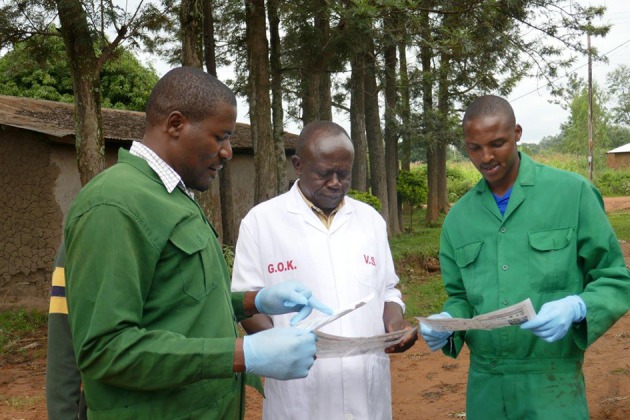
Public engagement sessions consisting of discussions with the meat inspector and slaughterhouse workers at sentinel slaughterhouses and slabs. Consent has been obtained from all participants identifiable in the photos.

**Figure 5:**
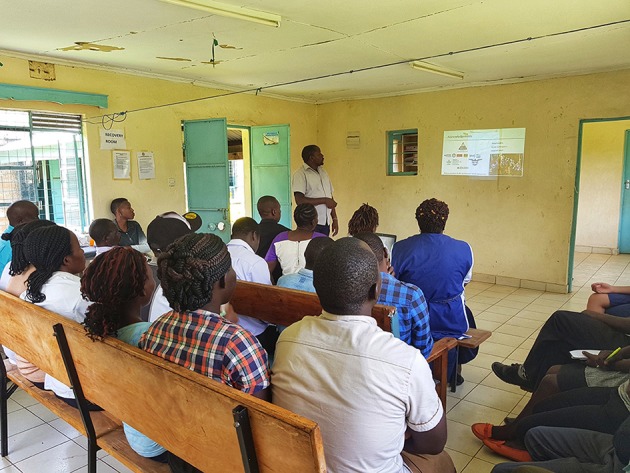
Public engagement sessions consisting of presentations during organized CME sessions at sentinel hospitals. Consent has been obtained from all participants identifiable in the photos.

**Figure 6:**
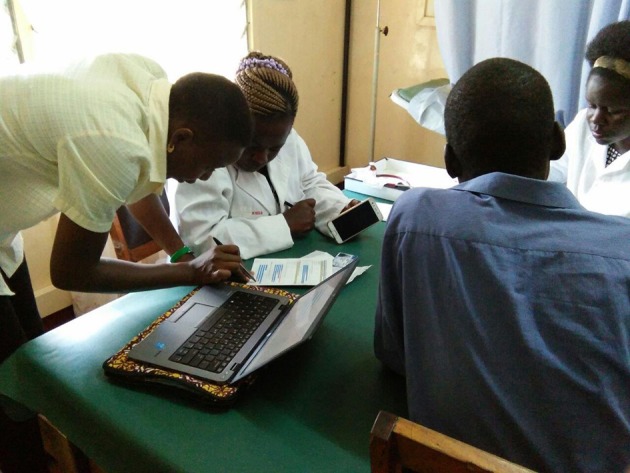
Public engagement sessions consisting of discussions with individual stakeholders at the sentinel hospitals. Consent has been obtained from all participants identifiable in the photos.

A public engagement session was conducted at each sentinel site (Figures 3–**6**). At livestock markets, between 30 and 200 stakeholders per session were engaged depending on how big and busy the market was, and sessions were primarily directed toward livestock traders, trekkers, farmers and other interested people within the market (Figure 3). At slaughterhouses and slabs, 10 to 30 stakeholders per session were engaged, often in smaller groups, and these included the meat inspector, slaughterhouse workers, and any butchers or traders present (Figure 4). Hospital engagements were conducted in small groups of 2–7 people, and during organized CME sessions (Figures 5, 6). The latter were attended by 30 to 60 participants each, including doctors, clinical officers, laboratory technologists and nurses. The information pamphlets distributed at each session were made available on the study website and can be viewed here: http://www.zoonotic-diseases.org/project/zoolink-project/.

## Discussion

In this paper we present the development of an integrated surveillance system for zoonotic diseases in western Kenya by integrating staff and structures for sample collection and analysis across animal and human sectors, and fostering engagement. In this report we focus specifically on the operational and logistical aspects, while discussing challenges in the implementation of, and opportunities arising from, such a system. Our intention is that lessons from our research-focussed surveillance activity will inform the public sector integration of surveillance across animal and human sectors, providing key data on efficacy, cost-effectiveness, coverage, and effort.

The described surveillance system is based on selected sentinel sites, namely livestock markets, slaughterhouses and slabs, and hospitals, which are repeatedly investigated. We recognize that this design may introduce some bias as the samples were passive and self-selected since we had no control over which animals and patients visited our sentinel sites. Furthermore, the sampled population in a sentinel design is not randomly selected and is therefore less representative of the target population (5). In this study we tried to ensure representativeness by including sites distributed in different geographical areas within the study area. Moreover, we included both smaller and more local livestock markets and slaughterhouses, and larger livestock markets and slaughterhouses with wider catchment areas. Finally, we included County hospitals which have a wider catchment area, sub-County hospitals which attend to more local patients, and Missionary hospitals which have higher fees and are therefore usually frequented by better-off people. We opted not to use a cross-sectional community survey as this has already been done (24), and we wanted to develop a system that is more efficient and cost-effective compared to traditional surveys, yet still captures changes in the amount and distribution of circulating pathogens over time.

While other integrated surveillance systems have been described, such as the Canadian Integrated Program for Antimicrobial Surveillance and the Danish Program for surveillance of antimicrobial consumption and resistance in bacteria from food animals, food and humans, to our knowledge this is the first surveillance system focusing on a broad array of zoonotic pathogens in both animal and human populations simultaneously. As such, analysis of its operation will be key to integrating the surveillance of neglected zoonoses in national surveillance systems. The novel design of this surveillance system allows us to investigate multiple zoonoses in livestock and human populations living in the area. This is particularly relevant for such an area where numerous zoonotic diseases persist endemically, with considerable impact on both human and animal health and welfare (20–24, 26). Additionally, such an approach provides us with the opportunity to explore other relevant diseases and public health issues alongside the main surveillance system, creating synergies and additional value. These surveillance data will therefore provide us with more accurate estimates of the burden and impact of disease in these populations, while also providing a basis for investigation of changes in the temporal and geographical distribution of these zoonoses. Moreover, such robust epidemiological data can then inform the development and parameterization of models to predict future disease trends in this study area which is undergoing significant demographic changes, with a consequent shift in agricultural systems from subsistence to more market-oriented, or explore the impact of different control strategies (29). Finally, a key element is to optimize the structure and design of the surveillance system itself, eventually moving beyond research-focussed activities to one integrated in the national surveillance system.

Integrated surveillance systems require political goodwill and commitment, together with adequate funding resources (9, 16). In Kenya, the Zoonotic Disease Unit, a government One Health coordinating unit at the national level (30), creates such a conducive political environment. In fact, the Zoonotic Disease Unit has a vision to devolve integrated activities—including surveillance—to regional level, thus providing a policy environment in which integrated surveillance is linked to government departments, and can thus proceed relatively smoothly. A principal aim of our work was to provide the evidence base for the design of this devolution.

Another requisite for the implementation of such integrated activities is a multi-disciplinary team. The core team involved in this project includes epidemiologists, veterinarians, animal health assistants, clinical officers, microbiologists, laboratory technologists, and administrators. A broader team includes economists, biologists and anthropologists. Furthermore, this project relies on the engagement of various short term trainees who spend between 1 and 12 months working on the project, building their own skill base and professional training. While these trainees provide assistance with the daily activities, they receive hands-on training in epidemiology, laboratory techniques and public health, and are directly exposed to One Health thinking and implementation. Developing well-trained personnel is an important factor in ensuring the sustainability of such integrated endeavors (3, 10).

Throughout the implementation of field activities we have encountered numerous challenges and set-backs. In livestock markets, the most frequent challenge was livestock owners refusing to participate in the study, either because they were in a hurry to sell their animal, or because they feared that sampling would deter future buyers. Over the course of the study we strove to address these concerns by making our sampling more efficient, and by repeatedly explaining the rationale and benefits of our sampling. While in some sentinel sites the refusals persisted throughout the study period, in other sites they reduced or stopped over time (Figure 2), and we believe this is largely attributable to the public engagement sessions. Direct and regular feedback is important in maintaining stakeholder compliance, building involvement and empowerment directly within stakeholder communities (16, 31). In this study we witnessed first-hand the importance of providing regular feedback to all stakeholders and study participants. Public engagement sessions were iterative processes, tailored to context, where we continually revised the content of the information pamphlets and the mode of delivery based on feedback received. This ensured an open dialogue, and helped develop rapport and trust between all those involved, while enhancing knowledge and awareness of zoonoses in the region. The importance of such engagement extends beyond research, and should be sustained in any government-led community surveillance programmes.

A frequent challenge in slaughterhouses/slabs was a quick slaughter process which hindered our sampling. While this improved in some sentinel sites, it persisted in others. Over time we came to understand that this was due to pressure put on the slaughterhouse workers by the butchers who brought their animals for slaughter and were in a hurry to sell the meat. Sampling pigs at slaughterhouses has also proven to be a challenge. While the demand for pork meat in western Kenya, as elsewhere, is growing (32), much of the trading and slaughtering of pigs still happens at the household level (33). Presently, there are no consistent markets for pigs in western Kenya, and only a few rudimental pig slabs. This not only raises concerns regarding the safety of the pork meat (34), as it is often not inspected prior to consumption, but also hinders the implementation of surveillance for pig diseases at such sentinel sites. These challenges highlight the need to better understand the beef and pork value chain in western Kenya, including the governing power structure and actors involved.

Obtaining support and compliance from the public health sector has been more arduous, as compared to the animal health sector, as is often the case in One Health initiatives (9). In this study, hospital sampling was delayed due to challenges in obtaining permissions, and ongoing health-workers' strikes. When designing the sampling scheme, we expected it to be easier to sample patients at Referral hospitals, compared to sub-County hospitals, given the higher patient throughput at the former compared to the latter. However, the opposite proved to be true during the study, primarily since the Referral hospitals included in this study experienced frequent staff turn-overs, which meant that our staff had to re-introduce themselves and the study at each visit. Such issues are well-recognized as a constraint in the health sector across developing countries (35). Moreover, as the Referral hospitals were in general busier, they tended to be less willing to cooperate, as compared to the sub-County hospitals where it was easier to establish a rapport with the hospital personnel. Overall, this resulted in us sampling fewer patients than originally anticipated.

In each site we aimed to sample up to 10 animals or patients. This was easiest to achieve at the livestock markets, where the number of animals present at each market ranged between a 100 and 800 animals. Indeed, only rarely was the team unable to sample 10 animals in livestock markets. In contrast, it was not always possible to sample 10 animals in slaughterhouses, as this depended on the daily kill. Some of the selected slaughterhouses/slabs had a daily kill that ranged between 3 and 8 animals, while others slaughtered more than 30 animals in a day. Therefore, the proportion of animals sampled in a slaughterhouse could range between 30 and 100% of the animals presented for slaughter on the day. Lastly, we rarely sampled the maximum number of ten patients in hospitals, primarily due to the challenges described above, together with the low patient turn-out in some of the smaller hospitals.

A challenge common to all sampling sites was missing data, which was due to different reasons in the different sites. In livestock markets and slaughterhouses/slabs, blood and fecal, samples were missed due to challenges in restraining the animal or empty rectums, respectively. While the option of constructing crushes at each market to improve animal restraint was discussed at the start of the study, we realized that this would not be practical or feasible in many of the livestock markets where the owners would have had to move their animals over large distances to allow for sampling (markets themselves cover a broad area where selling and purchase take place). We therefore opted to restrain the animals manually to improve our chances of owners accepting to participate. In hospitals, we frequently missed stool or blood samples, either because the patient failed to turn up at the laboratory after being visited by one of our clinicians, or because they could not produce a stool sample. As mentioned earlier, we opted to have the blood sample taken by a hospital lab technologist, rather than one of our own staff, to avoid unnecessary invasive sampling. Similarly, while we discussed other options for collecting stool samples, such as taking rectal swabs, we agreed that these would be considered more obtrusive and, consequently, less likely to achieve compliance.

While this surveillance system integrates animal and human population data, it invariably misses other important facets involved in disease epidemiology, such as the role of wildlife, climatic factors, and vector data (8). However, in this study we chose to focus on making surveillance as efficient as possible, which is at odds with detailed household surveillance (24), and therefore certain trade-offs were consciously made.

This research-focussed surveillance activity served as a proof of principle for an integrated system, which can then allow us to make recommendations on a sustainable surveillance system that can be implemented in the long-term and, possibly, nation-wide. We believe that the general structure of co-located sentinel sites was successful as it allows for comparisons between the human and animal populations, while also fostering relationships between the two sectors and increasing awareness of zoonotic diseases. Furthermore, sampling on market days and including smaller, more local, hospitals, also optimized sampling and increased the catchment area. However, we recognize that this system is too costly and cannot be implemented as is. Data are currently being generated on the existing surveillance system in Kenya, and the socioeconomic aspects of the surveillance system implemented in this research project. These, together with disease data, will allow us to better evaluate attributes such as sensitivity, timeliness and cost-effectiveness of this health surveillance system (36). This, in turn, will inform our recommendations on which diseases to prioritize, which sites to visit and how often, and which clinical data and biological samples to focus on.

Our experiences have highlighted the importance of engaging with local stakeholders in the field, while also providing timely feedback through regular public engagement sessions, to ensure ongoing compliance. Findings have also shed light on future areas of research, including better understanding of the pork value chain in western Kenya and the promising use of social networks in promoting education and behavior change communication.

## Ethics Statement

This study was approved by the Institutional Animal Care and Use Committee (IACUC Reference No. 2017-04) and the Institutional Research Ethics Committee (IREC Reference No. 2017-08) at the International Livestock Research Institute, review bodies approved by the Kenyan National Commission for Science, Technology and Innovation. Approval to conduct the work was also obtained from the Department of Veterinary Services and the Ministry of Health, and the relevant offices of these Ministries at devolved government level.

## Author Contributions

LF implemented the study and wrote the manuscript. EK, SK, JR, MW, and EF conceived the study. LA, FA, KM, PM, MM, SN, AO, and JO devised and implemented the field and laboratory protocols. All authors participated in drafting the manuscript and approved the final version.

### Conflict of Interest Statement

The authors declare that the research was conducted in the absence of any commercial or financial relationships that could be construed as a potential conflict of interest.
