# Supplementary material for: One Health in Action: Operational Aspects of an Integrated Surveillance System for Zoonoses in Western Kenya
Source: Front Vet Sci. 2019 Jul 31;6:252. doi: 10.3389/fvets.2019.00252 (PMC6684786; doi:10.3389/fvets.2019.00252)
Supplement: Supplementary file 1 [file Table_1.DOCX]

**Sampling frame for ZooLinK project**

**1. Bungoma County**

|  | **Livestock markets** | | | | | | **Slaughterhouses/slabs*** | **Hospitals** |
| --- | --- | --- | --- | --- | --- | --- | --- | --- |
| **sub-County** | **Mon** | **Tues** | **Wed** | **Thurs** | **Fri** | **Sat** |  |  |
| **Bungoma South/**  **Kanduyi** |  | Mianga Chwele  Sangalo | Dorofu  Bukembe |  | Bungoma town | Mianga Chwele | Bungoma South (class B)    Nandolia (small)  Bukembe  Mechi meru | **Bungoma Referral hospital**  St. Damiano Missionary hospital  sub-County hospital |
| **Bumula** | Mateka |  | **Mianga** | Mateka |  | Mianga | **Mianga**  Buyofu  Kimaeti (quite active) | **sub-County hospital** |
| **Webuye** |  |  | **Webuye** |  | Lugulu |  | **Webuye (class B)**  Bokoli (not very active)  Misikhu | **Lugulu Missionary hospital**  sub-County hospital |
| **Kimilili** |  | Kamukuywa |  | **Kimilili** |  |  | **Kimilili (class B)**  Kamukuywa | sub-County hospital |
| **Bungoma North** | Mbakalu |  | Ndalu |  | Tongaren |  | Naitiri  Ndalu | sub-County hospital |
| **Chwele** | **Chwele** |  |  |  | Chwele |  | **Chwele (active)** | **sub-County hospital** |
| **Bungoma West** | Malakisi |  | Sirisia |  |  |  |  | sub-County hospital |
| **Mount Elgon** | Kapsokwony | Kapkaten |  |  | Cheptais | Kapkaten | Cheptais (active)  Kaptama | sub-County hospital |

*All these slaughterhouses are ruminant slaughterhouses; there are no pig slaughterhouses or slabs in Bungoma county

** Sites in blue and bold font are those selected for the study

**2. Busia County**

|  | **Livestock markets** | | | | | | | **Slaughterhouses/slabs** | **Hospitals** |
| --- | --- | --- | --- | --- | --- | --- | --- | --- | --- |
| **sub-County** | **Mon** | **Tues** | **Wed** | **Thurs** | **Fri** | **Sat** | **Sun** |  |  |
| **Teso North** | Amogoro |  |  | **Angurai** |  | Malaba |  | **Malaba** [ruminants]  Amagoro  [pigs and ruminants]  Angurai  [pigs and ruminants]  Kolanya  [pigs and ruminants]  Moding  [pigs and ruminants] | Amagoro sub-County hospital  Moding sub-County hospital |
| **Teso South** |  | **Amukura** | Adongosi  Lukolis  Kemodo |  |  |  |  | **Amukura [ruminants]**  Amerikwai [ruminants]  Burumba [pigs]  Adongosi [ruminants]  Lukolis [ruminants]  Angorom [pigs]  Asinge  [pigs and ruminants]  Amairo [pigs]  Machakus [ruminants]  Amoni [pigs]  Apokor [pigs]  Simbachai  [pigs and ruminants]  Kotur [pigs] | Alupe sub-County hospital  Amukura sub-County hospital  **Lukolis sub-County hospital** |
| **Matayos [Busia]** |  |  |  |  |  |  |  | Busia town abattoir [ruminants]  Mundika  [pigs and ruminants]  Matayos  [pigs and ruminants]  Ekero [pigs] | **Busia Referral hospital** |
| **Nambale** |  | Mungatsi |  |  |  | Nambale |  | Nambale  [pigs and ruminants]  Kemodo  [pigs and ruminants]  Tangakona [pigs]  Nasewa [pigs]  Busibwabo  [pigs and ruminants]  Mungatsi  [pigs and ruminants] | Nambale sub-County hospital |
| **Butula** |  | **Bumala-Butula** | Lugula | K’Oloo |  |  | Bumala-Butula | Butula  [pigs and ruminants]  **Bumala**  **[pigs and ruminants]**  Bujumba  [pigs and ruminants] | **Butula Missionary hospital**  Bujumba sub-County hospital |
| **Bunyala** | Harambe |  |  |  |  | Budalangi  Port Victoria |  | Budalangi  [pigs and ruminants]  Mundare [pigs]  Harambe  [pigs and ruminants]  Morambwa  [pigs and ruminants]  Maumau  [pigs and ruminants]  Port Victoria  [pigs and ruminants] | Port Victoria sub-County hospital |
| **Samia** |  |  |  | **Funyula** |  |  |  | **Funyula**  **[pigs and ruminants]**  Iganga [ruminants]  Bukiri  [pigs and ruminants]  Namboboto  [pigs and ruminants]  Ganjala  [pigs and ruminants]  Sioport  [pigs and ruminants] | Holy Family Missionary Hospital  **Sioport sub-County hospital** |

**3. Kakamega County**

|  | **Livestock markets** | | | | | | **Slaughterhouses/slabs** | **Hospitals** |
| --- | --- | --- | --- | --- | --- | --- | --- | --- |
| **sub-County** | **Mon** | **Tues** | **Wed** | **Thurs** | **Fri** | **Sat** |  |  |
| **Butere** | Butere |  |  |  |  |  | Butere town | Butere sub-County hospital |
| **Khwisero** |  |  | Imanyulia |  |  | Imanyulia |  |  |
| **Lurambi** |  | Kambi mwanza |  | **Lubao** |  |  | Kakamega town  **Lubao**  [pigs and ruminants] | **Kakamega Referral hospital**  Shamakhobu sub-County hospital |
| **Navakholo** | Nambacha |  |  |  |  |  | Navakholo town  Nambacha | Navakholo sub-County hospital |
| **Shinyalu** |  |  | **Shinyalu** |  |  | Shinyalu | Khayega town  [pigs and ruminants]  **Shinyalu**  [pigs and ruminants] | **Shinyalu sub-County hospital** |
| **Malava** |  |  | Butali | Lubao |  |  | Malava | Malava sub-County hospital |
| **Lugari** |  |  |  |  |  | Matete  Matunda | Lugari |  |
| **Ikolomani** |  | **Musoli/Shikulu** |  |  |  |  | **Musoli/Shikulu** [pigs]  Shivagala [ruminants] | Ikolomani sub-County hospital  **Mukumu Missionary hospital** |
| **Likuyani** |  |  |  |  |  |  |  |  |
| **Matungu** | **Koyonzo** |  |  | Ogalo |  |  | **Harambee** [ruminants]  **Koyonzo** [ruminants] | **Matungu sub-County hospital** |
| **Mumias east** |  |  | Makunga |  | Mumias town |  | Mumias town | Makunga sub-County hospital  Mumias Missionary hospital |
| **Mumias west** |  | Ejenje |  |  |  |  |  |  |
