# Supplementary material for: One Health in Action: Operational Aspects of an Integrated Surveillance System for Zoonoses in Western Kenya
Source: Front Vet Sci. 2019 Jul 31;6:252. doi: 10.3389/fvets.2019.00252 (PMC6684786; doi:10.3389/fvets.2019.00252)
Supplement: Supplementary file 5 [file Table_5.DOCX]

# ZooLinK sampling protocol for cattle, sheep and goats at livestock markets

**Protocol operational:**

**Version:** 1

**Created:** November 2016

**Last saved:** March 2017

**Primary authors:** Laura Cristina Falzon

**Secondary authors:** James Akoko, Maurice Karani, Kelvin Momanyi, Patrick Muinde, Joseph Ogola

**Total number of pages:** 11

1. **Sampling strategy:**

Twelve livestock markets have been selected (4 in each county). Each of these shall be visited every four weeks, over a two year period. Thus, each location shall be sampled for a total of 24 times during this study.

At each livestock market:

- The location and GPS coordinates of the site shall be automatically recorded by the tablet.
- We shall aim to sample up to 10 randomly selected animals: 6-7 cattle and 3-4 small ruminants (there are no pigs at the livestock markets). Selection will be done either by standing at the entrance of the market and marking every *n*^th^ animal (where *n*=approx. animal population at the market /10), and then sampling the marked animals once they have entered the market premises, or by counting the animals inside the market fence and sampling every *n*th animal.
- In addition to these 10 randomly sampled animals, any other animals that look emaciated and/or are suspected of being infected with a zoonotic disease of interest, shall also be sampled but their data shall be treated and analysed separately.
- Once the animals have been identified and/or marked, the person responsible for the animal shall be identified, the study scope and procedure shall be explained to them, and permission to sample the animal and to ask the accompanying person relevant questions shall be sought.
- If the person responsible for the animal agrees to participate in the study and signs the consent form*,* the animal shall be restrained and sampling and data collection will ensue.

1. **Animal handling and restraint:**

All animals should be securely restrained by a competent handler prior to commencing sampling.

- - 1. **Cattle:**
- Restrain the animal with a rope halter and/or tethering to the fence or, if not strong enough, a nearby tree or strong poll. An additional handler, if available, may hold the tail.
- Use a crush to restrain the animal if one is available.
- Care must be taken with horned animals; keep one hand on horn at all times when dealing with head.
  - 1. **Sheep and goats:** Restrain the animal manually by holding between legs.

1. **Animal Identification and clinical examination:**

**IN THE EVENT OF THE ANIMAL PROVING DIFFICULT TO HANDLE THE CLINICAL EXAMINATION AND SAMPLING WILL BE DISCONTINUED.**

Use a combination of your own knowledge and the animal owner’s information when making observations about the animal’s breed, age, sex and physiological state, etc.

**3.1 Body Condition Scoring**

- - 1. **Cattle** (as described in the “Condition scoring of dairy cows”, available at: <http://assurance.redtractor.org.uk/contentfiles/Farmers-5476.pdf>)
- The scoring method involves a manual assessment of the thickness of fat cover and prominence of bone at the tail head and loin area.
- You should stand directly behind the cow to score both areas and always handle the animal quietly and carefully using the same hand. The tail-head is scored by feeling for the amount of fat around the tail-head and the prominence of the pelvic bones.
- The loin is scored by feeling the horizontal and vertical projections of the vertebrae and the amount of fat in-between. Assessment relies mainly on the tail-head, but is refined by the loin score if both are very different.
- On a scale of 1-5, a score of 1 is extremely thin and a score of 5 is extremely fat (see pictures below for further information). If possible, assess the scores to the nearest half point.
- You may also choose to take a picture of the animal from the back and side.


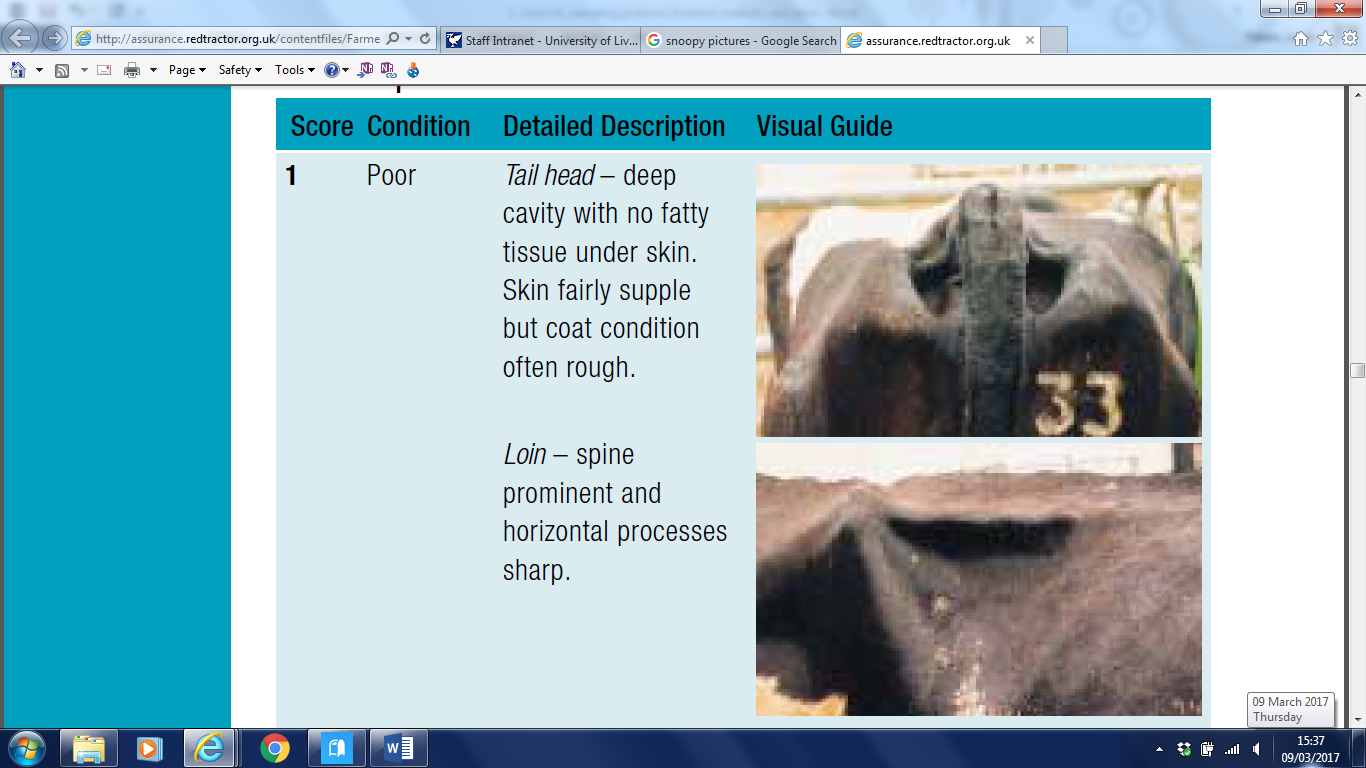


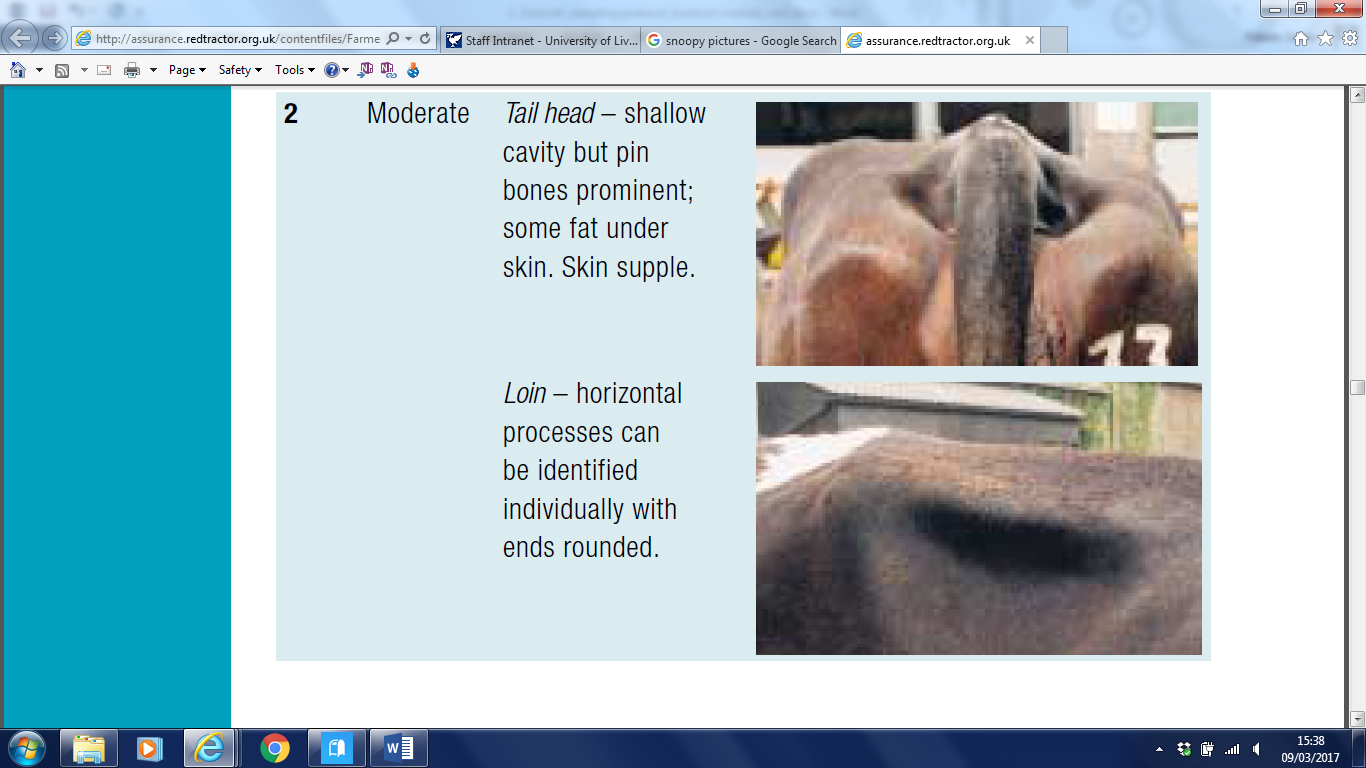


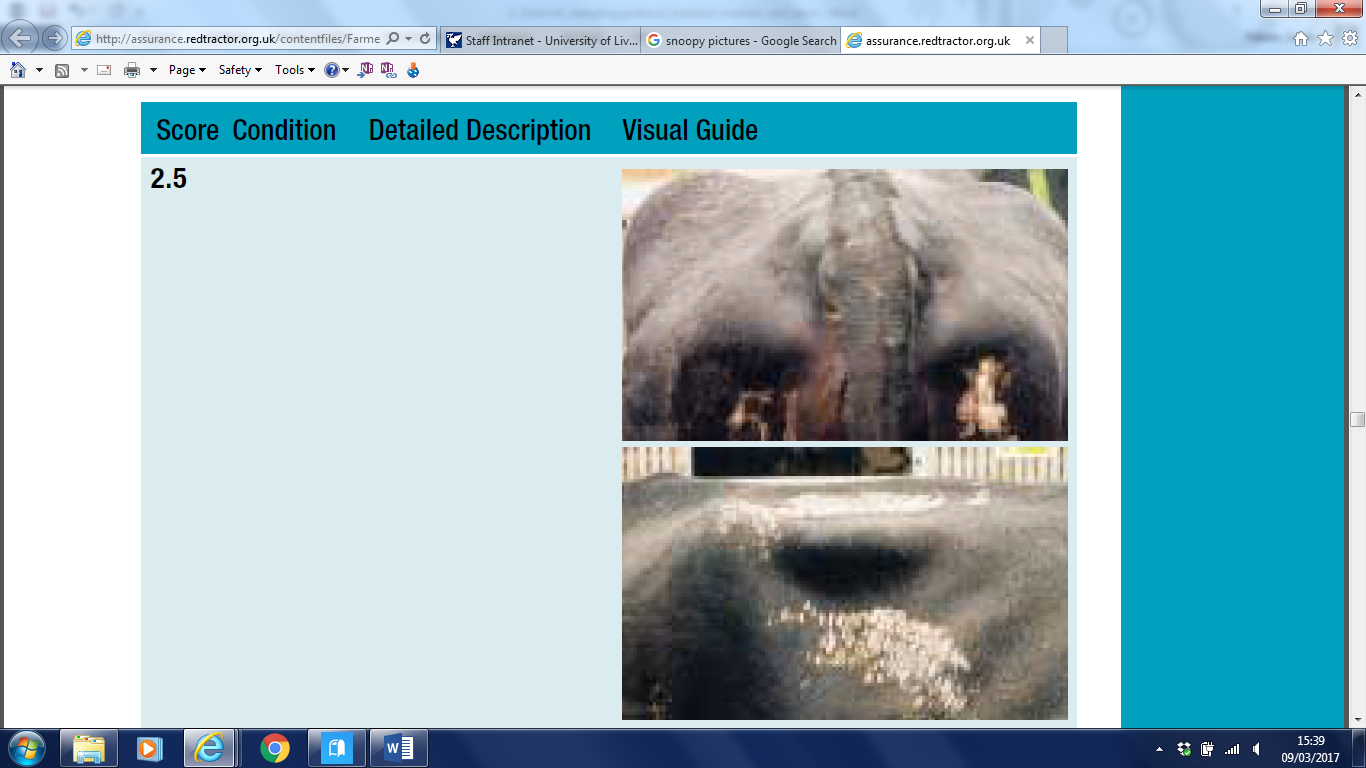


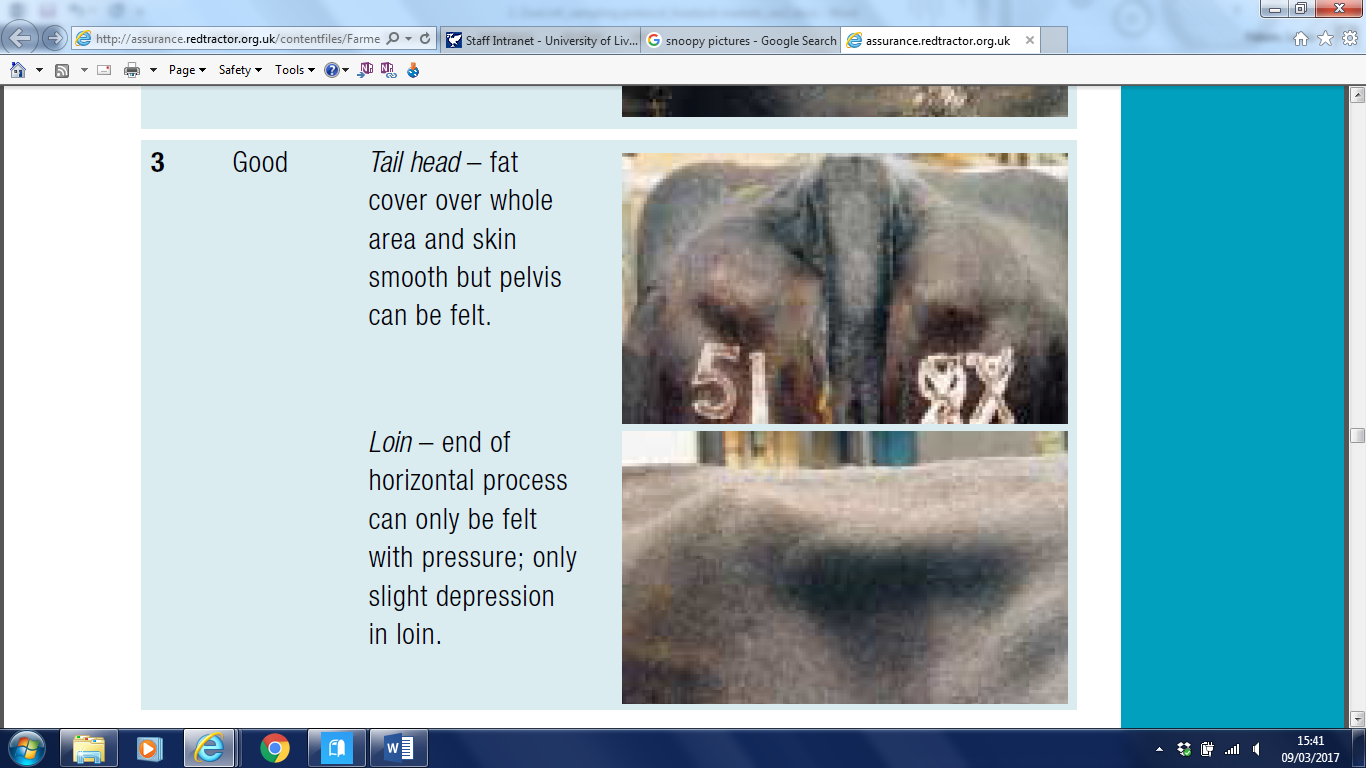


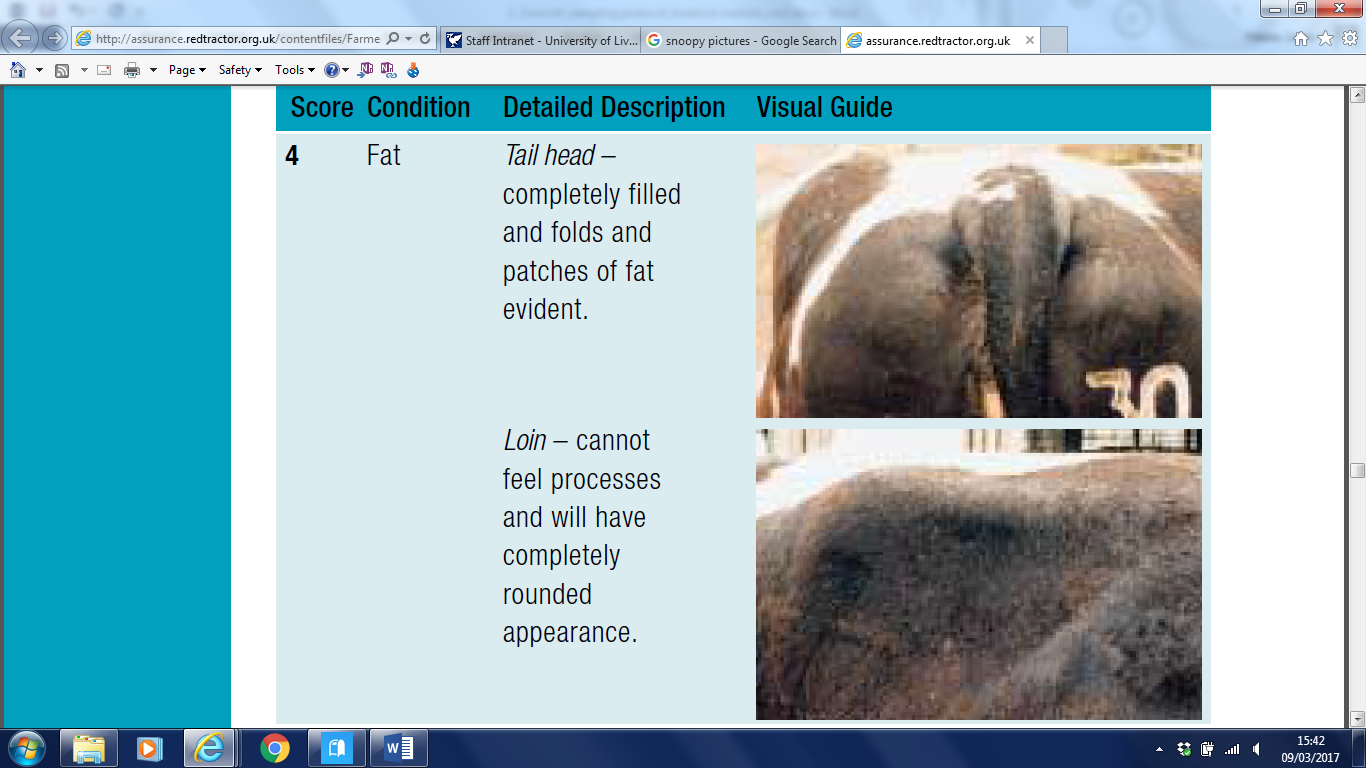


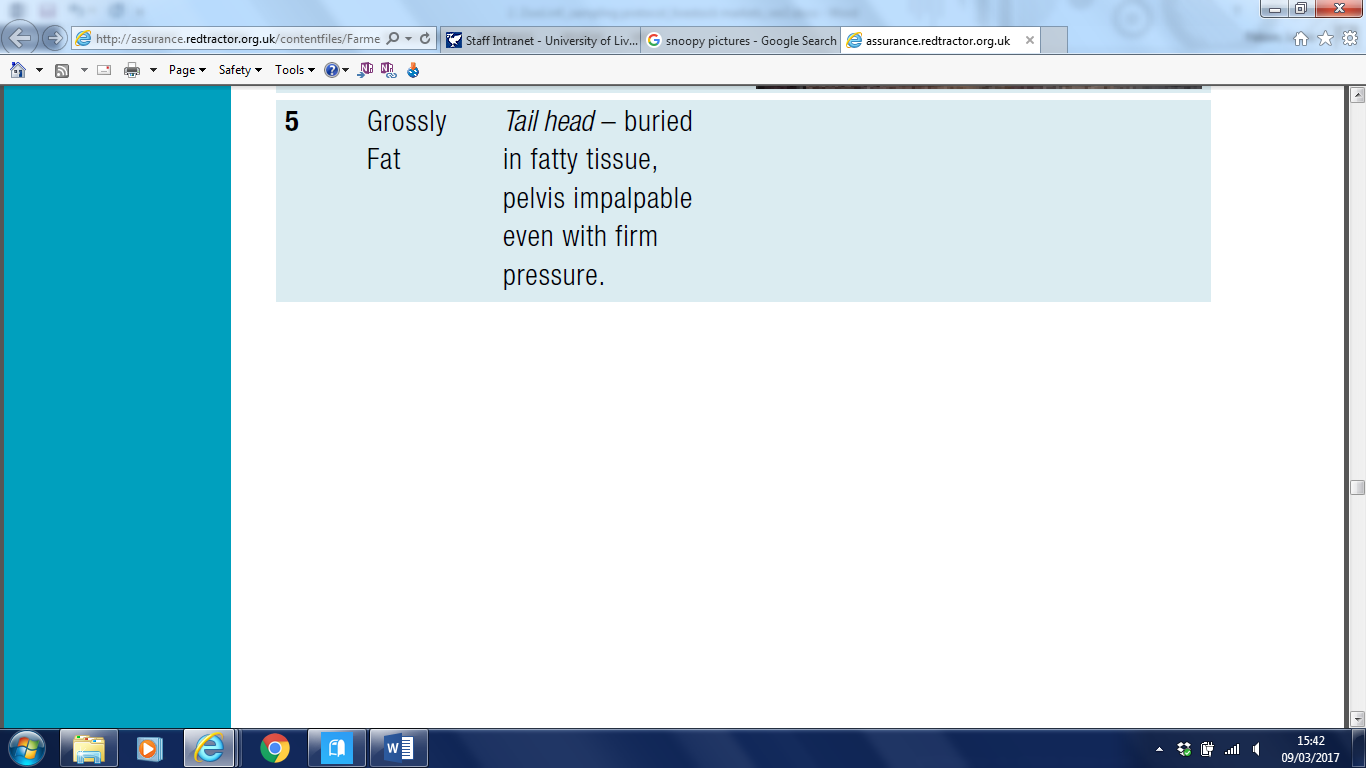


3.1.2 **Sheep and goats** (as described in the “Technical Bulletin No.8: Body Condition Scoring of Sheep and Goats”, available at: <http://www.esgpip.org/Pdf/Technical%20Bulletin%20No.%208.pdf>)

- The body scoring of sheep and goats shall be done using a BCS ranging from 1.0 to 5.0, with 0.5 increments, where an animal of BCS 1.0 is extremely thin with no fat reserves and an animal of BCS of 5.0 is a very over-conditioned (obese) animal (Table 1).


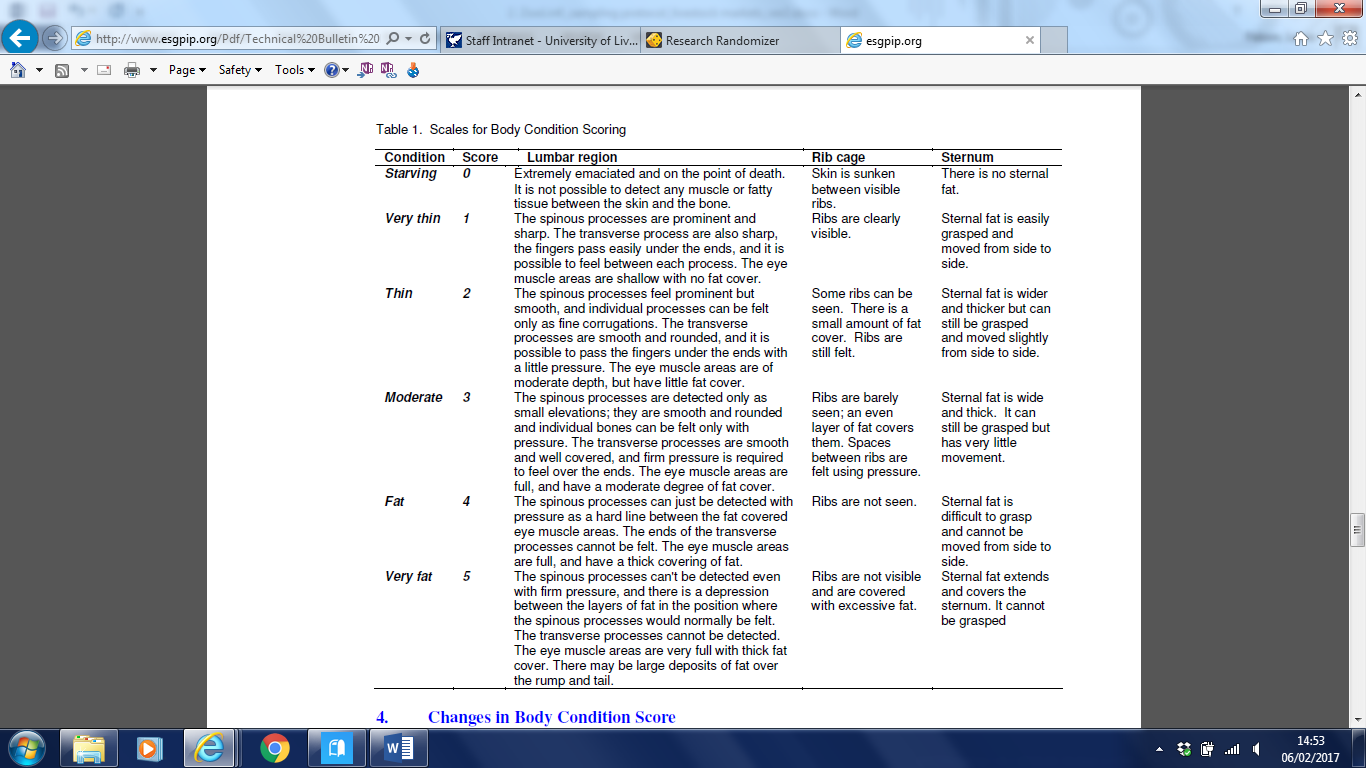


- To assign a BCS, one must touch and feel the animal. In sheep, the lumbar region is the principal site for BCS determination while in goats the rib cage and sternum also play a role.
- The lumbar region contains the loin muscle and is located immediately behind the last rib and before the hip bones. Scoring in this area is based on determining the amount of fat and muscle over and around the vertebrae. Lumbar vertebrae have two protrusions: the vertical protrusion called the spinous process, and the two horizontal protrusions called the transverse process. You should run your hand over this area and try to grasp these processes with your fingertips and hand as shown in Figures 1 to 2. The degree of sharpness or roundness of the lumbar vertebrae is assessed and used to assign BCS.
  - Feel the spinous process in the centre of the sheep/goat back behind the last rib and in front of the hip bone and try to rank the animal based on the answers you give to the following question. Are the tips sharp or rounded?
- Feel the fullness of muscle and fat cover on either side of the spinous process (either side of the backbone) and determine if the ridge of the spine is above the level of the muscle. Is the loin muscle shallow, moderate or full?
- Feel for the tips of the transverse process. Is it sharp or smoothly rounded? How far will the tips of your finger go under the transverse process?

Figure 1. Spinous and transverse process. Figure 2. Spinous process.

- The second area to assess, particularly in goats, is the rib cage and fat cover on the ribs and intercostal (between ribs) spaces. Touch this area and determine if you can feel each of the ribs.
- The sternum is the third part to assess. In goats it is an important area to assess. The fat cover over the sternum (breast bone) is based upon the amount of fat that can be pinched (Fig. 3).

Figure 3. The sternum area

**3.2 Skin Neck Elasticity**

With the animal standing or sitting, tent out the skin on either side of the neck and then release. If the skin immediately falls back into place normally the animal is scored as “normal”. If the skin is slow to return to normal then it is scored as “abnormal”.

- 1. **Rectal Temperature**

- Temperatures are to be measured using the digital thermometer.

- Switch on the thermometer and insert into the rectum. Angle the sensor towards the rectal wall to avoid the sensor ending up in a faecal mass where the reading will be inaccurate. Do not remove the thermometer until you hear it beeping.

- In the event of low [≤ 38°C] or high [>39.5 °C] temperatures the procedure should be repeated to confirm.

- 1. **Lymph node palpation**

Palpate the superficial lymph nodes (including: the parotid, sub-mandibular, pre-scapular, retro-pharyngeal, pre-crural and supra-mammary lymph nodes) using index and middle finger, and thumb. Any anomalies or enlargements (uni- or bi-laterally) should be noted.

- 1. **Examination for external parasites, vesicles, lesions, sores or discharge**

The animal should be visually inspected to check for:

1. The presence of external parasites

- Pay attention to particular areas, such as ears, shoulders, dewlap, axillae, belly, udder and groin.
- An effective way to detect adult ticks, especially when they are engorging, is to feel the hair coat of the host with the palm of your hand. To find immature or unfed adults the hair can be parted systematically using forceps.
- Remove any ticks (max. 5 per animal) using good quality steel forceps to grip the tick firmly at the base of the mouthparts as closely to the host skin as possible, and then pull strongly and directly out from the skin. Ticks should be stored in watertight containers with cotton wool imbibed in ethanol.

1. The presence of vesicles, lesions or sores

Pay attention to particular areas, such as withers, limbs, joints and around the coronary band. The size and location of any vesicle, lesion or sore present should be noted.

1. The presence of discharge

Check for the presence of any discharge from the eye, ear, nose, mouth, external genitalia, and anus. If present, describe location, quantity, colour and consistency.

**3.6 Mucous membranes**

Examine the conjunctival and gingival mucosae and note any anomaly, e.g. cyanosis (blueish colour), anaemia (pallor), congestion (deep red), or jaundice (yellowish colour). Use the FAMACHA Chart described below to assess anaemia levels:

**3.6.1 FAMACHA Chart**

- Read Carefully the FAMACHA information pamphlet.

- Examine the animal in good natural light.

- Open the eyelid; push the upper eyelid down with the upper thumb, while the lower thumb gently pulls the lower lid downward. Look especially at the colour inside the lower eyelid.

- Open the eyelid for a short time only, or else the mucous membrane may become redder. For this reason the FAMACHA classification must be done before the clinical examination of the eye takes place.

- Compare the colours seen to those on the reverse side of the FAMACHA card and score the animal from 1 to 5. If redness is < 1 then consider conjunctivitis. If in doubt, score the animal at the paler category.

- Keep in mind that certain conditions can make the eye’s membranes appear redder than expected and thus mask anaemia (e.g. dust, close sheds, heat, driving animals a long way with no rest period afterwards, any fever, infectious eye disease, any diseases associated with blood circulatory failure).

- The colours of the FAMACHA chart fade with time, especially if exposed to sun. Replace the card after 12 months use.

**3.7 Girth Weight Band**

- Get the animal in a normal standing position with all four legs in a vertical line

- Wrap the measuring tape round the thorax just behind the shoulder blade. Make sure there are no twists in it.

- Use the spring to hook onto the end and pull the tape tight using 2 kg tension. Read the length off in cm by matching up the 0cm with the closest mark on the other end of the tape.

1. **Sample collection:**

**4.1 Blood sampling:**

Collect blood from the jugular vein using **two** 10ml plain (red top) vacutainer tube and a 4ml EDTA (purple top) vacutainer tube. The EDTA tubes should be inverted immediately after collection. For the BoviGam interferon test, blood must be collected in heparinised tubes.

**4.2 Stool Sampling:**

- At the time of taking rectal temperature, if no fresh faecal sample is forthcoming a manual or digital extraction should be attempted.

- The faecal sample (approximately 5g +) should be placed in a plastic bag, sealed and labelled.

**4.3 Milk Sampling:**

If the animal being sampled is a lactating female, clean teats using clean wipe and collect a milk sample directly into a clean, labelled plastic container.

**4.4 Nasal Swabs:**

- Clean any obvious dirt from around the outside of the nostrils with a disposable cloth.

- If swabs are in packets, open from the stem end. Be careful not to touch the swab tip at any point during sampling

- Gently place the swab inside the end of the nostrils, ensuring it is against the internal surface of the mucosa. Rotate the swab a couple of times.

- After sampling, swabs should be put into plain tubes with transport medium, then snap off the tip and seal.

**4.5 Tissue Sample (using the Allflex Tissue Sampling Unit as described in:** <http://www.allflexusa.com/assets/Detail-Sheets/TSU%20Application%20Guide_R4.pdf>**):**

- Remove a Tissue Sampling Unit form the packaging and load the AllFlex Tissue Applicator [the red plunger is visible in used punches].

- Ensure the tube retainer at the base of the applicator gun is open. Push the retainer clip to open.

- Insert the punch into the tissue applicator and release the clip to lock punch into the device.

- Carefully squeeze the applicator handles together, guiding the punch tip into place if necessary. When fully seated, the gun bolt will rest flush against the red plastic clip.

- Release the handle, and remove the red plastic clip by pulling it outward. Take care not to cut finger on the metal cutter as it is very sharp.

- Slide the gun over the ear and position the cutter approximately 1 inch from the edge of the animal’s ear, taking care to avoid any obvious veins and ridges.

- Squeeze handles together to take a sample and then release to free the ear. Try to do this in one swift, fluid motion. Move with the animal and do not fight its movements.

- Remove the punch from the device and check that sampling has been successful. If not, discard the sample and re-sample with a new punch.

- Remove the used cutter from the applicator by pulling the handles apart. This will loosen the cutter. Discard safely.

1. **Marking animal:**

Once the sampling is finished, mark the animal on the back with a non-toxic spray paint.

# ZooLinK sampling protocol for cattle, sheep, goats and pigs at slaughterhouses

**Protocol operational:**

**Version:** 1

**Created:** November 2016

**Last saved:** March 2017

**Primary authors:** Laura Cristina Falzon

**Secondary authors:** James Akoko, Maurice Karani, Kelvin Momanyi, Patrick Muinde, Joseph Ogola

**Total number of pages:** 13

1. **Sampling strategy:**

Slaughterhouse have been selected in each county (1 or 2 next to each of the 12 livestock markets selected to participate in this study). Each of these shall be visited every four weeks, over a two year period. Thus, each location shall be sampled for a total of 24 times during this study.

At each slaughterhouse:

- The location and GPS coordinates of the site shall be automatically recorded by the tablet.
- We shall aim to sample up to 10 slaughter animals on each visit (from one or multiple premises visited on the day): 3-4 pigs, 3-4 cattle and 2 small ruminants; or 6-7 cattle and 3-4 small ruminants (if no pigs are being slaughtered in any of the premises visited on that day). Therefore, all animals (if ≤10 animals are to be slaughtered in the selected premise(s) on that day) or a sub-sample of the animals (if >10 animals are to be slaughtered in the selected premise(s) on that day) shall be sampled.
- Any animals that are dead on arrival or whose carcasses are condemned after slaughter shall also be sampled, and additional data relating to the presumed cause of the animals’ death or organ condemnation shall be collected.
- Once the animals have been identified and/or marked, the person responsible for the animal shall be identified, the study scope and procedure shall be explained to them, and permission to sample the animal and to ask the accompanying person relevant questions shall be sought.
- If the person responsible for the animal agrees to participate in the study and signs the consent form*,* the animal shall be restrained and sampling and data collection will ensue.

1. **Animal handling and restraint:**

All animals should be securely restrained by a competent handler prior to commencing sampling.

- 1. **Cattle:**
- Restrain the animal with a rope halter and/or tethering to the fence or, if not strong enough, a nearby tree or strong poll. An additional handler, if available, may hold the tail.
- Use a crush to restrain the animal if one is available.
- Care must be taken with horned animals; keep one hand on horn at all times when dealing with head.
  1. **Sheep and goats:** Restrain the animal manually by holding between legs.
  2. **Pigs:** Restrain with a pig snare behind the incisors.

1. **Animal Identification and clinical examination:**

**IN THE EVENT OF THE ANIMAL PROVING DIFFICULT TO HANDLE THE CLINICAL EXAMINATION AND SAMPLING WILL BE DISCONTINUED.**

Use a combination of your own knowledge and the animal owner’s information when making observations about the animal’s breed, age, sex and physiological state, etc.

**3.1 Body Condition Scoring**

- - 1. **Cattle** (as described in the “Condition scoring of dairy cows”, available at: <http://assurance.redtractor.org.uk/contentfiles/Farmers-5476.pdf>)
- The scoring method involves a manual assessment of the thickness of fat cover and prominence of bone at the tail head and loin area.
- You should stand directly behind the cow to score both areas and always handle the animal quietly and carefully using the same hand. The tail-head is scored by feeling for the amount of fat around the tail-head and the prominence of the pelvic bones.
- The loin is scored by feeling the horizontal and vertical projections of the vertebrae and the amount of fat in-between. Assessment relies mainly on the tail-head, but is refined by the loin score if both are very different.
- On a scale of 1-5, a score of 1 is extremely thin and a score of 5 is extremely fat (see pictures below for further information). If possible, assess the scores to the nearest half point.
- You may also choose to take a picture of the animal from the back and side.


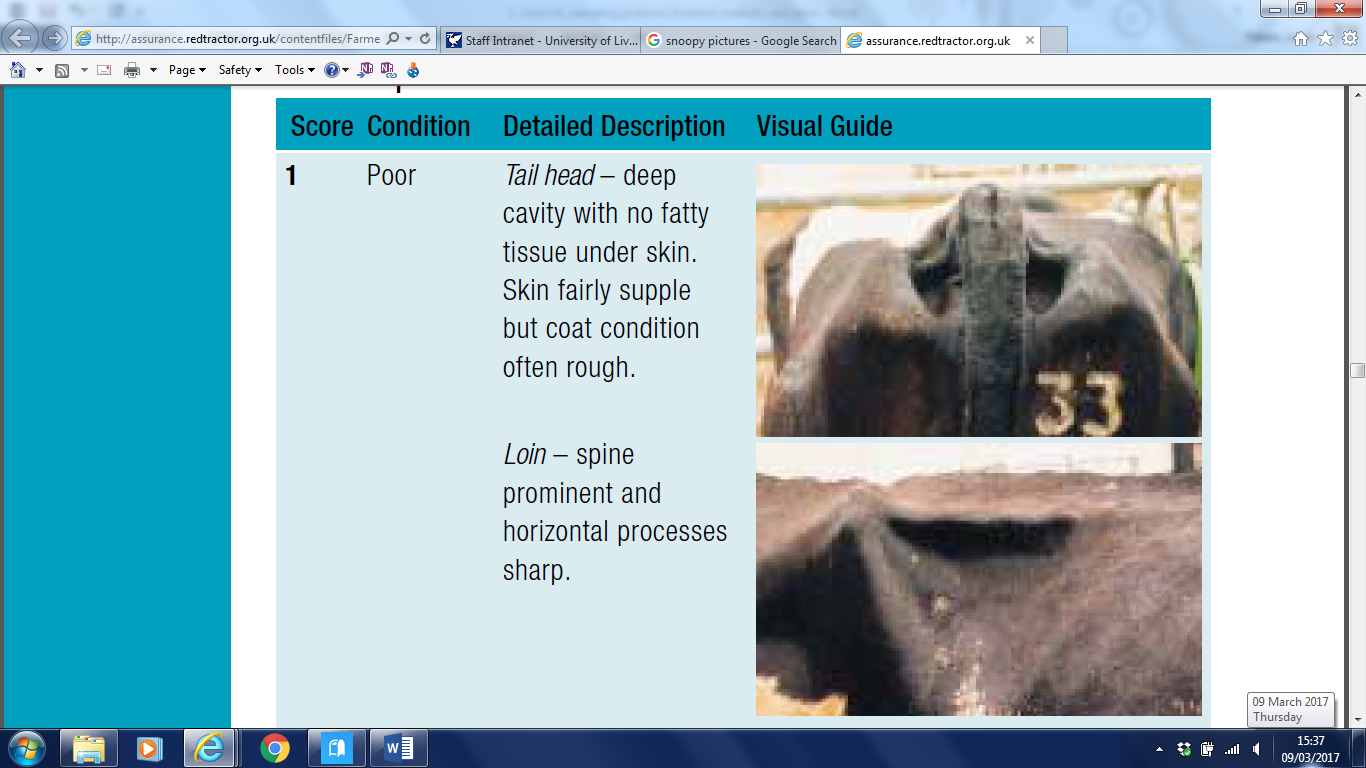


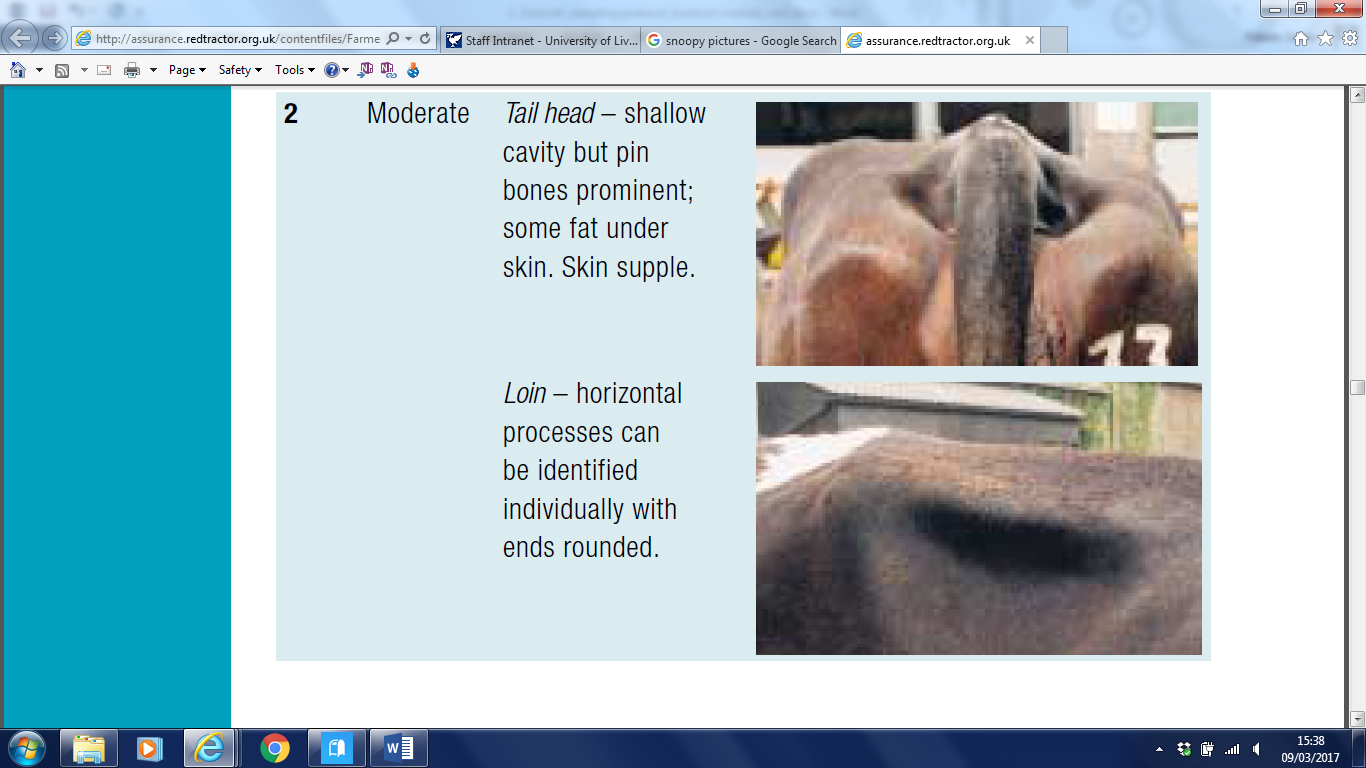


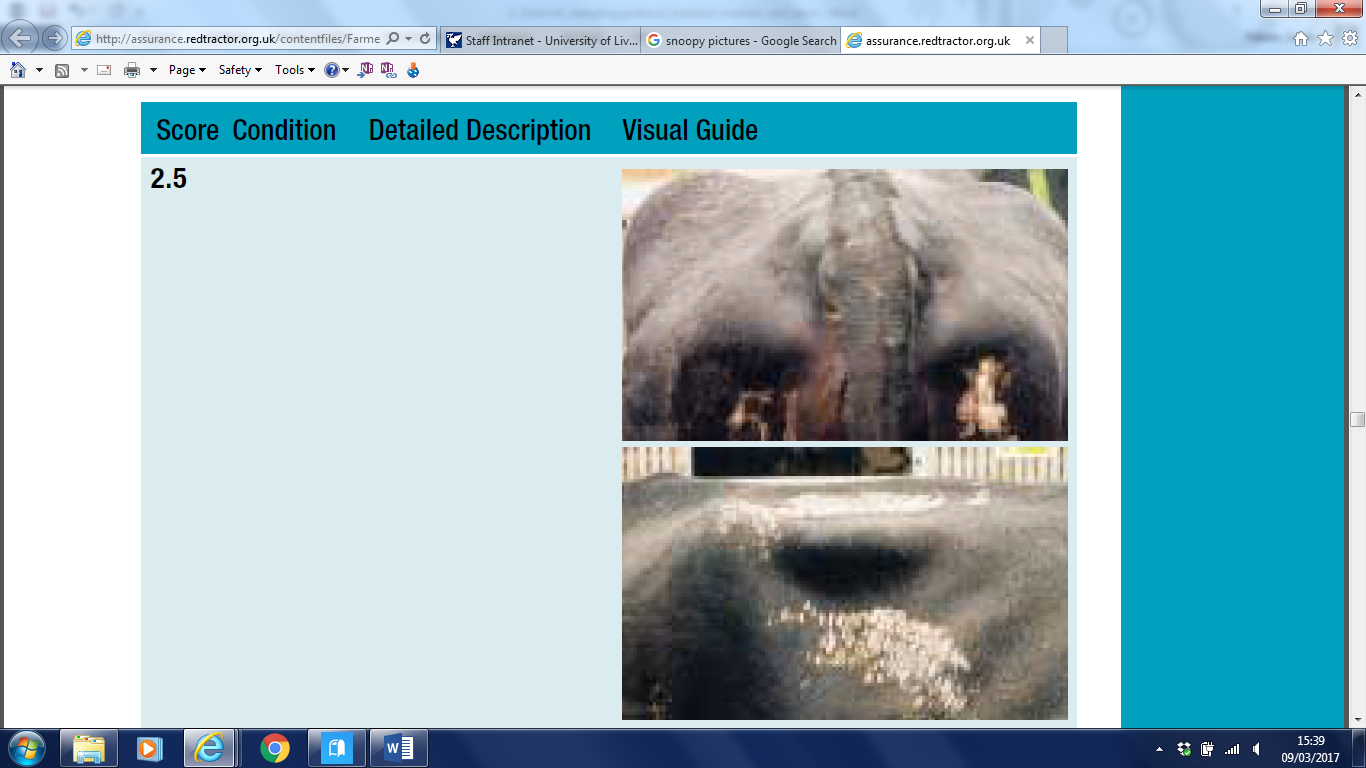


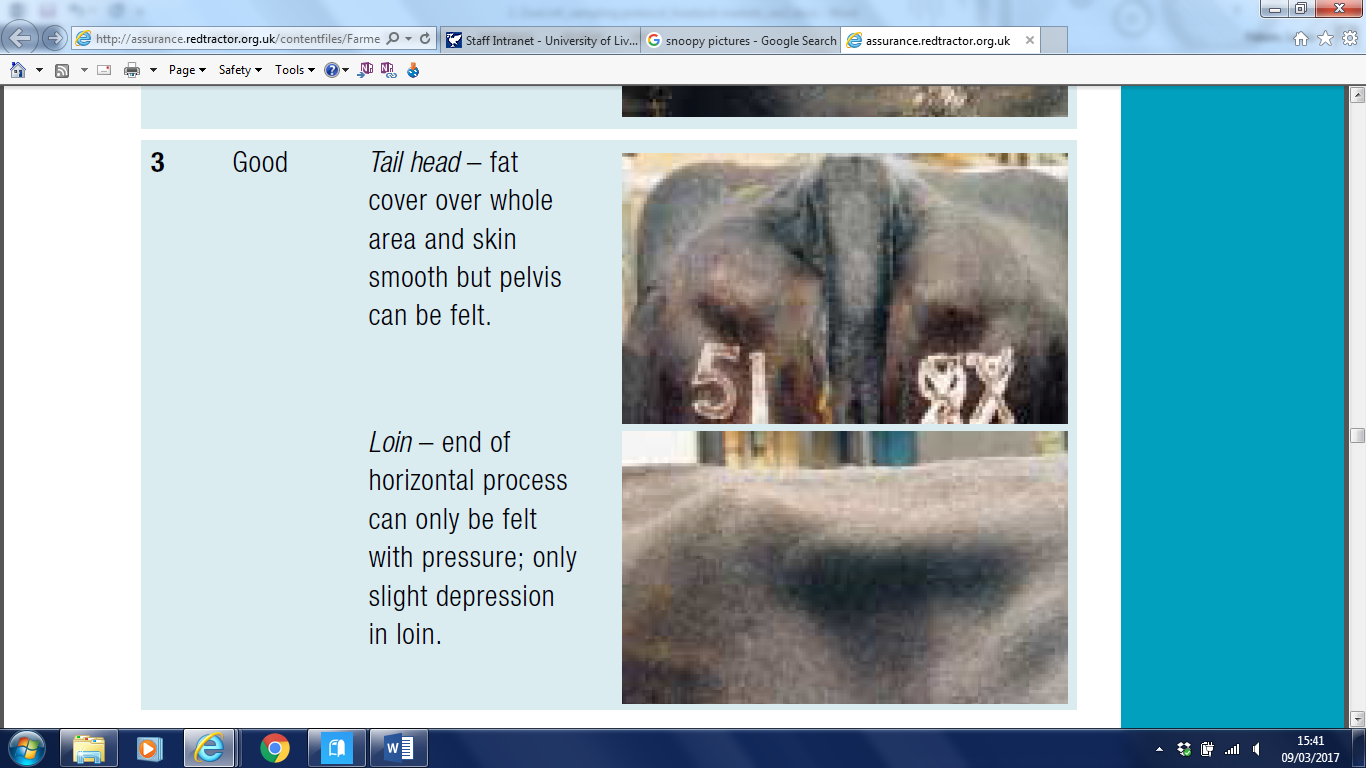


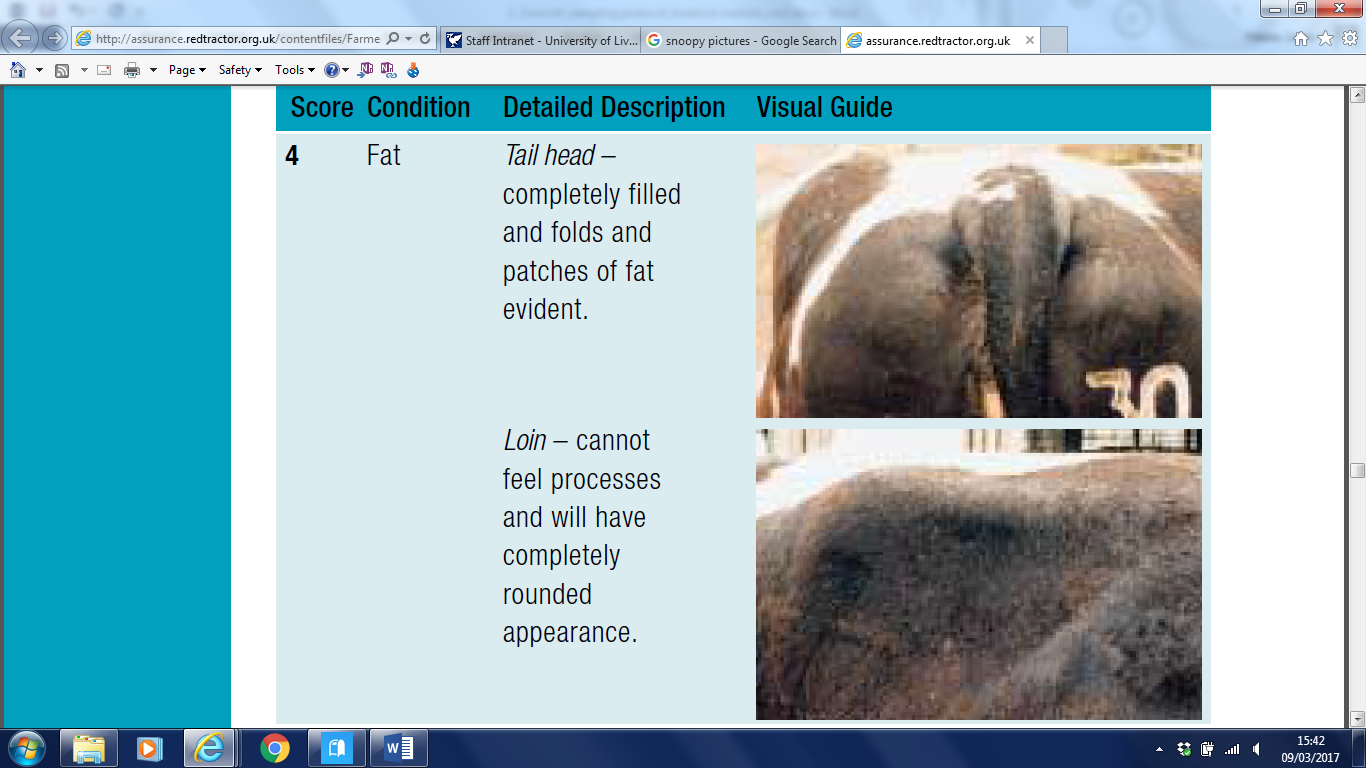


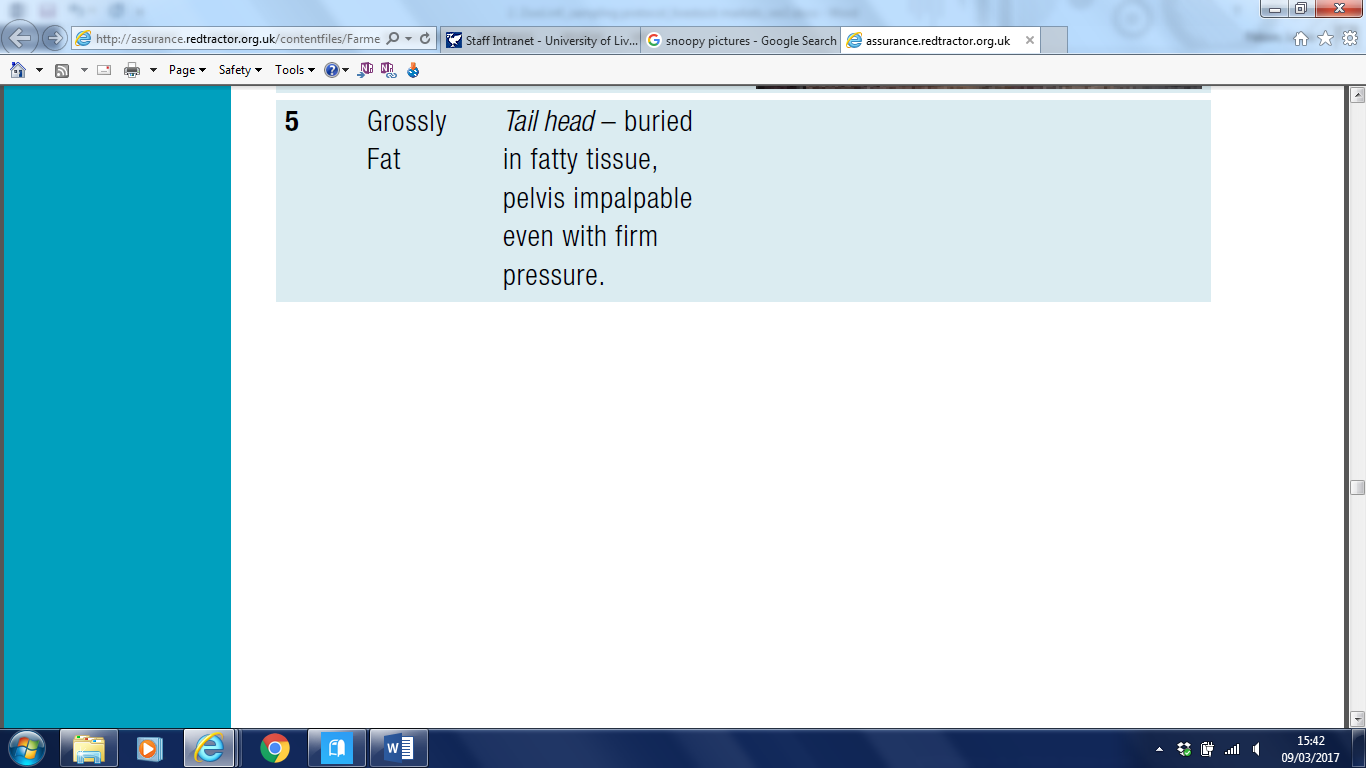


3.1.2 **Sheep and goats** (as described in the “Technical Bulletin No.8: Body Condition Scoring of Sheep and Goats”, available at: <http://www.esgpip.org/Pdf/Technical%20Bulletin%20No.%208.pdf>)

- The body scoring of sheep and goats shall be done using a BCS ranging from 1.0 to 5.0, with 0.5 increments, where an animal of BCS 1.0 is extremely thin with no fat reserves and an animal of BCS of 5.0 is a very over-conditioned (obese) animal (Table 1).


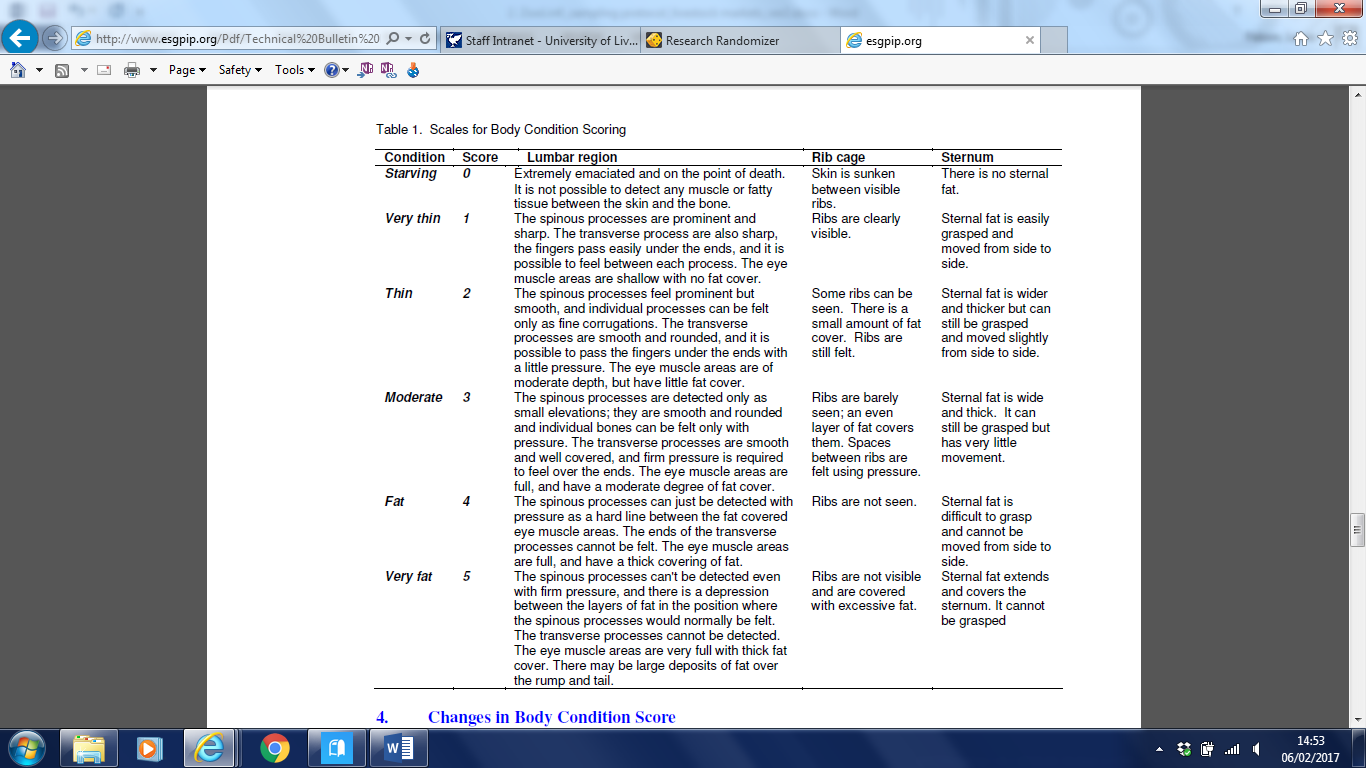


- To assign a BCS, one must touch and feel the animal. In sheep, the lumbar region is the principal site for BCS determination while in goats the rib cage and sternum also play a role.
- The lumbar region contains the loin muscle and is located immediately behind the last rib and before the hip bones. Scoring in this area is based on determining the amount of fat and muscle over and around the vertebrae. Lumbar vertebrae have two protrusions: the vertical protrusion called the spinous process, and the two horizontal protrusions called the transverse process. You should run your hand over this area and try to grasp these processes with your fingertips and hand as shown in Figures 1 to 2. The degree of sharpness or roundness of the lumbar vertebrae is assessed and used to assign BCS.
  - Feel the spinous process in the centre of the sheep/goat back behind the last rib and in front of the hip bone and try to rank the animal based on the answers you give to the following question. Are the tips sharp or rounded?
- Feel the fullness of muscle and fat cover on either side of the spinous process (either side of the backbone) and determine if the ridge of the spine is above the level of the muscle. Is the loin muscle shallow, moderate or full?
- Feel for the tips of the transverse process. Is it sharp or smoothly rounded? How far will the tips of your finger go under the transverse process?

Figure 1. Spinous and transverse process. Figure 2. Spinous process.

- The second area to assess, particularly in goats, is the rib cage and fat cover on the ribs and intercostal (between ribs) spaces. Touch this area and determine if you can feel each of the ribs.
- The sternum is the third part to assess. In goats it is an important area to assess. The fat cover over the sternum (breast bone) is based upon the amount of fat that can be pinched (Fig. 3).

Figure 3. The sternum area

3.1.3 **Pigs** (as described in “The Pig Site”, available at: <http://www.thepigsite.com/stockstds/23/body-condition-scoring/>)

- Observe the animal from the side and from behind, and palpate the animal along the spine, to make an assessment from 1-5 based on the given guide. You may also choose to take a picture of the animal from the back and side.

| **Score** | **Condition** | **Description** | **Shape of Body** |
| --- | --- | --- | --- |
| 5 | Overfat | Hips and Backbone heavily covered | Bulbous |
| 4.5 |  | | |
| 4 | Fat | Hips and backbone cannot be felt | Tending to bulge |
| 3.5 |  | | |
| 3 | Normal | Hips and backbone only felt with firm palm pressure | Tube shape |
| 2.5 | Somewhat thin | Hips and backbone felt without palm pressure | Tube shape but flat sides |
| 2 | Thin | Hips and backbone felt easily | ribs and spine can be felt |
| 1.5 |  | | |
| 1 | Emaciated | Hips and backbone viable | Bone structure apparent |


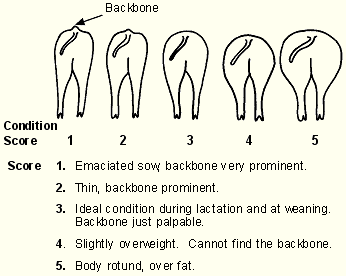


**3.2 Skin Neck Elasticity**

3.2.1 Ruminants: with the animal standing or sitting, tent out the skin on either side of the neck and then release. If the skin immediately falls back into place normally the animal is scored as “normal”. If the skin is slow to return to normal then it is scored as “abnormal”.

- 1. **Rectal Temperature**

- Temperatures are to be measured using the digital thermometer.

- Switch on the thermometer and insert into the rectum. Angle the sensor towards the rectal wall to avoid the sensor ending up in a faecal mass where the reading will be inaccurate. Do not remove the thermometer until you hear it beeping.

- In the event of low [≤ 38°C] or high [>39.5 °C] temperatures the procedure should be repeated to confirm.

- 1. **Lymph node palpation**

Palpate the superficial lymph nodes (including: the parotid, sub-mandibular, pre-scapular, retro-pharyngeal, pre-crural and supra-mammary lymph nodes in ruminants; and the superficial cervical, inguinal/mammary and sub-iliac lymph nodes in pigs) using the index finger, middle finger, and thumb. Any anomalies or enlargements (uni- or bi-laterally) should be noted.

- 1. **Examination for external parasites, vesicles, lesions, sores or discharge**

The animal should be visually inspected to check for:

1. The presence of external parasites

- Pay attention to particular areas, such as ears, shoulders, dewlap, axillae, belly, udder and groin.
- An effective way to detect adult ticks, especially when they are engorging, is to feel the hair coat of the host with the palm of your hand. To find immature or unfed adults the hair can be parted systematically using forceps.
- Remove any ticks (max. 5 per animal) using good quality steel forceps to grip the tick firmly at the base of the mouthparts as closely to the host skin as possible, and then pull strongly and directly out from the skin. Ticks should be stored in watertight containers with cotton wool imbibed in ethanol.

1. The presence of vesicles, lesions or sores

Pay attention to particular areas, such as withers, limbs, joints and around the coronary band in ruminants, and the snout and limbs in pigs. The size and location of any vesicle, lesion or sore present should be noted.

1. The presence of discharge

Check for the presence of any discharges from the eye, ear, nose, mouth, external genitalia, and anus. If present, describe location, quantity, colour and consistency.

**3.6 Mucous membranes**

Examine the conjunctival and gingival mucosae and note any anomaly, e.g. cyanosis (blueish colour), anaemia (pallor), congestion (deep red), or jaundice (yellowish colour). Use the FAMACHA Chart described below to assess anaemia levels:

**3.6.1 FAMACHA Chart**

- Read Carefully the FAMACHA information pamphlet.

- Examine the animal in good natural light.

- Open the eyelid; push the upper eyelid down with the upper thumb, while the lower thumb gently pulls the lower lid downward. Look especially at the colour inside the lower eyelid.

- Open the eyelid for a short time only, or else the mucous membrane may become redder. For this reason the FAMACHA classification must be done before the clinical examination of the eye takes place.

- Compare the colours seen to those on the reverse side of the FAMACHA card and score the animal from 1 to 5. If redness is < 1 then consider conjunctivitis. If in doubt, score the animal at the paler category.

- Keep in mind that certain conditions can make the eye’s membranes appear redder than expected and thus mask anaemia (e.g. dust, close sheds, heat, driving animals a long way with no rest period afterwards, any fever, infectious eye disease, any diseases associated with blood circulatory failure).

- The colours of the FAMACHA chart fade with time, especially if exposed to sun. Replace the card after 12 months use.

**3.7 Girth Weight Band**

- Get the animal in a normal standing position with all four legs in a vertical line

- Wrap the measuring tape round the thorax just behind the shoulder blade. Make sure there are no twists in it.

- Use the spring to hook onto the end and pull the tape tight using 2 kg tension. Read the length off in cm by matching up the 0cm with the closest mark on the other end of the tape.

1. **Sample collection:**
   1. **Blood sampling:**

4.1.1 Ruminants: Collect blood from the jugular vein using **two** 10ml plain (red top) vacutainer tubes and a 4ml EDTA (purple top) vacutainer tube. The EDTA tubes should be inverted immediately after collection. For the BoviGam interferon test, blood must be collected in heparinised tubes.

4.1.2 Pigs: Collect blood from the cranial vena cave using **two** 10ml plain (red top) vacutainer tubes and a 4ml EDTA (purple top) vacutainer tube. The EDTA tubes should be inverted immediately after collection.

**4.2 Stool Sampling:**

- At the time of taking rectal temperature, if no fresh faecal sample is forthcoming a manual or digital extraction should be attempted.

- The faecal sample (approximately 5g +) should be placed in a plastic bag, sealed and labelled.

**4.3 Milk Sampling:**

If the animal being sampled is a lactating female, clean teats using a clean wipe and collect a milk sample directly into a clean, labelled plastic container.

**4.4 Nasal Swabs:**

**-** Clean any obvious dirt from around the outside of the nostrils with a disposable cloth.

- If swabs are in packets, open from the stem end. Be careful not to touch the swab tip at any point during sampling.

- Gently place the swab inside the end of the nostrils, ensuring it is against the internal surface of the mucosa. Rotate the swab a couple of times.

- After sampling, swabs should be put into tubes with transport medium, then snap off the tip and seal.

**FOLLOWING SLAUGHTER, THE SAME ANIMAL WILL BE EXAMINED POST-MORTEM**

1. **Post-mortem examination and organ/tissue collection:**
   1. **Sample collection:**

- Using a scalpel blade, collect samples of mesenteric and tonsillar lymph nodes, liver and lung (if any lesions are present, e.g. tuberculosis; liver flukes), and any foetal/placental material present. Also collect a skin biopsy (e.g. ear notch). All samples should be placed in labelled, transparent containers.
- Aspirate the fluid from any vesicles or cysts identified.

**5.2 Tissue collection (using the Allflex Tissue Sampling Unit as described in:** <http://www.allflexusa.com/assets/Detail-Sheets/TSU%20Application%20Guide_R4.pdf>**):**

- Remove a Tissue Sampling Unit form the packaging and load the AllFlex Tissue Applicator [the red plunger is visible in used punches].

- Ensure the tube retainer at the base of the applicator gun is open. Push the retainer clip to open.

- Insert the punch into the tissue applicator and release the clip to lock punch into the device.

- Carefully squeeze the applicator handles together, guiding the punch tip into place if necessary. When fully seated, the gun bolt will rest flush against the red plastic clip.

- Release the handle, and remove the red plastic clip by pulling it outward. Take care not to cut finger on the metal cutter as it is very sharp.

- Slide the gun over the ear and position the cutter approximately 1 inch from the edge of the animal’s ear, taking care to avoid any obvious veins and ridges.

- Squeeze handles together to take a sample and then release to free the ear. Try to do this in one swift, fluid motion.

- Remove the punch from the device and check that sampling has been successful. If not, discard the sample and re-sample with a new punch.

- Remove the used cutter from the applicator by pulling the handles apart. This will loosen the cutter. Discard safely.

**5.3 Tongue palpation in pigs:**

Open the mouth of the pig, grip the tongue and palpate ventral side for the presence of cyst-like nodules. Any nodules detected should be incised using a scalpel blade and stored in a container.

# ZooLinK sampling protocol for human patients at hospitals

**Protocol operational:**

**Version:** 1

**Created:** December 2016

**Last saved:** March 2017

**Primary authors:** Laura Cristina Falzon, Lorren Alumasa, Fred Amanya

**Secondary authors:**

**Total number of pages:** 7

1. **Sampling Strategy**

The three County referral hospitals shall be visited every other week; in the alternate weeks, three Missionary hospital and three sub-County hospitals (one for each County) shall be visited. Therefore, the referral hospitals shall be visited for a total of 48 times, while the three missionary hospitals and three health centres shall each be visited for a total of 24 times over the two-year study period.

At each hospital and health facility:

- The location and GPS coordinates of each site shall be automatically recorded by the tablet.
- The hospital records on the number of patient admissions in the last four weeks shall be copied, and patient identifiers shall be omitted to preserve patient anonymity.

- We aim to sample 10 out-patients registered at the hospital and with suspected zoonotic disease, on each visit.

To be considered eligible, the patient must:

- Have at least one of the following symptoms (suggestive of a zoonotic disease):

- fever (defined as a body temperature ≥37.5°C )
- anaemia
- back pain
- muscle pain
- joint pain
- chest pain
- severe headache
- epileptic episodes
- loss of coordination
- respiratory signs (e.g. difficulty breathing, cough, phlegm)
- skin lesions
- diarrhoea
- vomiting
- nausea
- abdominal cramps
- general malaise
- loss of appetite
- sudden weight loss
- bleeding
- abortion
- loss of vision [in children]

- Not have a lab-confirmed diagnosis of malaria and/or dengue.

- If the patients are already registered, a random number generator shall be used to randomly select 10 patients; otherwise, every *n*th patient that is registered and that meets our inclusion criteria shall be asked to participate in the study.

- While children shall also be sampled (if the accompanying person consents), we shall ensure that these are not overly represented in our screened population, as this may then not be representative of our overall target population. We shall therefore set a limit of children to be included (e.g. maximum 5 out of the 10 patients?).

- Once the person to be sampled has been identified, the study scope and procedure shall be explained to them, and permission to take specific samples and ask questions shall be sought. In the case of minors, assent will be sought from the legal guardian.

- If the patient consents to participate in the study and signs the required documentation, sampling and data collection shall ensue.

**2. Patient identification and clinical examination:**

2.1 Ask demographic details

2.2. Collect history of patient

2.3. General physical examination

2.3.1. Measure patient’s height and weight

2.3.1.1. Weight

- Use the weighing scales (to be carried by the field team) and ensure that the scale is placed on a flat wooden surface

- Ensure the digital reading reads 0 before the participant stands on the scale

- Read the weight in kg

2.3.1.2. Height

- Use the standard height measure which should be assembled/disassembled at each hospital and health centre

- Ensure the height measure is placed against a wall and straight

- Ensure the participant stands with both feet flat (NOT SHOES) on the base plate

- Read the height in cm

2.3.2. Measure the Mid-Upper Arm Circumference (MUAC)

- Use the MUAC tape for both adults and children. The tape is a coloured plasticized tape for assessing levels of malnutrition. It can be used for children from the age of 6 months and also for adults. The tape is marked in millimetre readings with cut-off from red to yellow at 110mm and yellow to green at 125mm.

- Place the tape around the left limb mid-upper arm. The tape should be tight, but should not wrinkle the skin.

- Pass the tail through the window marked “T” and read the number which shows as the most complete in the window marked “mm”, e.g. in the image below, the reading should be 154 mm.

2.3.3. Measure axillary temperature

- Record temperature using a digital thermometer, under the arm. Do not remove the thermometer until you hear it beeping.

- Express the reading in degrees Celsius, to one decimal place.

- Ensure that the thermometer is cleaned using surgical spirit between participants

2.3.4. Take the patient’s pulse

2.3.5. Measure the patient’s blood pressure

- Use a sphygmomanometer to measure the patient’s blood pressure.

- Record the systolic and diastolic blood pressures separately.

2.3.6. Eye and mouth examination

2.3.7. Check for Oedema

2.3.8. Check for Pain

2.3.8. Perform auscultation (respiratory sounds)

2.3.9. Perform thoracic and abdominal percussion

**3. Sample Collection:**

- 1. **Blood sampling**

- Obtain verbal consent from the participant asking if they would be willing to provide a blood sample.

- A single use vacutainer butterfly needle and adapter MUST be used for drawing blood.

- A clean (non-sterile) pair of gloves MUST be used on every participant.

- Ensure patient is comfortable and ask them to extend their arm.

- Ask the patient to form a fist for the veins to be more prominent.

- Locate a vein that is of good size and visible, preferably the median cubital vein.

- Put on well-fitting clean gloves.

- Apply tourniquet about 4-5 finger widths above the venipuncture site.

- Disinfect the site with 70% alcohol swabs starting from the center of the venipuncture site outwards and allow the area to dry completely. DO NOT touch the disinfected area.

- Using the right size needle, while holding the patient’s arm to anchor the vein, access the vein swiftly at 30^0^ angle or less and draw blood using a 10ml pain tube (or less depending on age) and 4ml EDTA tube (**NOTE:** This can be discussed further).

- Release the tourniquet then withdraw the needle.

- Give patient a dry cotton wool to apply gentle pressure on the injection site to stop bleeding and direct them to the infectious biological waste bag for disposal once done.

- Discard the used vacutainer butterfly needle immediately in the sharps container (DO NOT Recap).

- Invert the EDTA tube immediately and a number of times.

- Label samples with barcodes and scan.

- Place sample in the cool box for transportation.

- Remove gloves and place in infectious waste bag.

- Wash hands with soap and water.


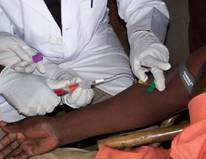


- 1. **Stool sampling**

- Ask the participant if they would be willing to provide a fecal sample.

- Fecal pots should be provided once the participant consents.

- Indicate that the stool sample should be collected from material that has not been in direct contact with the ground using the integrated pot spoon.

- The fecal sample should be as fresh as possible, preferably produced on the morning of sample collection.

- Ideally, the stool should not be contaminated with urine.

- Ideally, a large teaspoon quantity should be collected or 10ml if it is fluid.

- Two swabs should be collected from the freshly passed stool specimen (in the fecal pot) with the provided swabs by project clinicians.

- While collecting swabs from a stool specimen, insert the tip of each swab into the stool and remove. Examine the swabs to ensure that they are stained with fecal matter.

- Insert the fecal stained swabs immediately into the tube containing transport medium, pushing the swab to the bottom of the tube into the medium. Break off and discard the top part of the swab and tighten the screw top of the medium container firmly.

- While wearing gloves, label both the sample pot and the swabs with barcodes from the barcode reel and scan using the tablet.

- Immediately place samples in the cool box.

- Remove gloves and wash hands with soap and water.

- 1. **Sputum sampling**

- Obtain verbal consent to collect the sputum sample. Only patients with productive cough are included.

- Sterile specimen caps should be provided.

- First thing in the morning sample is preferable.

- Discourage brushing of teeth or mouthwash as this would kill bacteria in the sputum.

- Have the patient rinse their mouth with plain water to avoid contaminating sputum with bacteria from the mouth.

- Sample should be taken before breakfast or at least an hour after breakfast.

- Patient to take at least 3 deep breaths then force out a deep cough. Emphasize on bringing out sputum from the lungs and not saliva or secretions from the mouth.

- Ask patient to uncap the container and avoid touching the inside.

- Cough out in a secluded area, away from other patients to avoid cross infection and spit in the container.

- Patient should securely cap the container and wash hands with soap and water.

- Wearing gloves, collect the specimen.

- Label with barcodes and scan.

- Place sample in cool box for transportation.

- Remove gloves and wash hands with soap and water.

- 1. **Nasal swabs**

- Ask participant if they are willing to have two nasal swabs taken.

- Put on clean pair of gloves for each patient

- If swabs are packed, open from the stem end and avoid touching the swab tip even during sample collection.

- Gently insert the tip of each swab into one nostril and rotate against the nostril wall.

- Place the swab in a dry tube, break off the tip of the stick and close the tube.

- Label each swab with a barcode and scan.

- Place the samples in the cool box.

- Remove gloves and clean hands with soap and water.
