# Supplementary material for: One Health in Action: Operational Aspects of an Integrated Surveillance System for Zoonoses in Western Kenya
Source: Front Vet Sci. 2019 Jul 31;6:252. doi: 10.3389/fvets.2019.00252 (PMC6684786; doi:10.3389/fvets.2019.00252)
Supplement: Supplementary file 6 [file Table_6.DOCX]

**Materials/consumables required**

1. **Livestock markets and slaughterhouses:**
2. General:

- Gloves
- Gumboots
- Overalls (3 pairs)
- Consent forms
- Tablets
- Printed list of random numbers
- Stationery: pens, notebooks, pencil, marker pen, clip-board, tape
- Barcoded labels
- Barcode scanner (installed in tablet)
- Hand sanitizer
- Disinfectant
- Field tool box and efficient/waterproof bags
- Rain coat

1. Restrain:

- Ropes
- Pig snare

1. Clinical examination:

- BCS table
- 3 different measuring tapes for the 3 different species (pigs, small ruminants and cattle)
- FAMACHA table
- 70% ethanol
- Cotton wool
- Forceps
- Scissors/clippers
- Kits for collecting tissue samples (Allflex tissue applicator and tissue punches)
- Digital thermometer
- Wipes to clean udder/teats in case of milk samples

1. Sampling

- Biological waste containers
- Sharp containers
- Faecal containers (polythene bags/pots)
- Vacutainer tubes
  - 10 ml plain (red top)
  - 4 ml EDTA (purple top)
- Vacutainer needles gauge 18-25
- Needle holder
- FLOQ nasal swabs (with transport medium)
- Scalpel blades
- Carcass swab sponges
- Syringes
- Tubes (falcon tubes for the ticks)
- Plastic/zip-lock bags and containers
- Disposable cloth during nasal swab collection
- Spray paint for marking
- Gentamicin spray (in case of open wounds during sampling)

1. Transport

- Cool box and ice packs
- Media for transport (e.g. saline solution)

1. **Hospitals**
2. General:

- Gloves (medium and large)
- Clean lab-coat
- Consent forms
- Tablets
- Printed list of random numbers
- Stationery: pens, notebooks, pencil, marker pen, clip-board, tape, rubber, ruler, paper punch, stapler, staple pins, spring/box files, laptop
- Barcoded labels
- Barcode scanner (installed in tablet)
- Hand sanitizer
- Disinfectant
- Field tool box and efficient/waterproof bags
- Face mask
- Rain coat

1. Clinical examination:

- MUAC (mid, upper, arm and circumference) measuring tape
- Height measure
- Weighing scale
- 70% ethanol
- Cotton wool
- Scissors
- Digital thermometer
- Watch
- Sphygmomanometer
- Stethoscope
- Tongue depressor
- Special interior mouth examination torch/light
- Menstrual calendar
- Tourniquets (2)
- Vacutainer butterfly needles

1. Sampling

- Biological waste containers
- Sharp containers
- Stool pots and swabs
- Vacutainer tubes
  - 10 ml plain (red top)
  - 4 ml EDTA (purple top)
- Vacutainer butterfly needles gauge 21-23
- Needle holder and adapter
- FLOQ nasal swabs (with transport media)
- Syringes (5 and 10ml)
- Sputum tubes
- Plastic bags and containers
- Needles (21-23)
- Urine containers
- Scalpel blades and skin tape

1. Transport

- Cool box and ice packs
- Media for transport (e.g. saline solution)
