# Supplementary material for: One Health in Action: Operational Aspects of an Integrated Surveillance System for Zoonoses in Western Kenya
Source: Front Vet Sci. 2019 Jul 31;6:252. doi: 10.3389/fvets.2019.00252 (PMC6684786; doi:10.3389/fvets.2019.00252)
Supplement: Supplementary file 7 [file Table_7.DOCX]

**Livestock Market Visit**

Site: _______________________ Date: ______________________

Time of arrival at site: _________________ Time of departure from site: _________________

Number of ZooLinK staff involved: ________

Number and type of local staff engaged (i.e. paid by project) (Please tick all applicable answers):

Livestock market chairperson

Livestock market master

County staff

Animal handler

Other; please specify: _________________________________________________________

Total number of animals sampled: ________

If fewer than 10, why? (Please tick all applicable answers)

Few animals in market

Many refusals

Other; please specify: _________________________________________________________

Number of animal owners who refused to have their animals sampled: _______________________

Reasons for refusals:

1. _________________________________________________________________________

2. _________________________________________________________________________

3. _________________________________________________________________________

4. _________________________________________________________________________

Number of animals with incomplete data: ________________________________________

| Missing data or sample | Reason |
| --- | --- |
|  |  |
|  |  |
|  |  |

How hard was it to get the samples today?

1. Very hard 2. Hard 3. Moderate 4. Easy 5. Very easy

**Slaughterhouse Visit**

Site: _______________________ Date: ______________________

Time of arrival at site: _________________ Time of departure from site: _________________

Number of ZooLinK staff involved: ________

Number and type of local staff engaged (i.e. paid by project) (Please tick all applicable answers):

Meat inspectors

Slaughterhouse workers

Other; please specify: _________________________________________________________

Total number of animals sampled: ________

If fewer than 10, why? (Please tick all applicable answers)

Fewer than 10 animals were slaughtered

Process too quick (i.e. insufficient time allowed to sample)

Animals already slaughtered by the time we arrive

Other; please specify: _________________________________________________________

**If multiple sites were visited on the same day, please complete the following section for each site separately (if only one site was visited, complete only once).**

Site: _____________________________

Number of animal owners who refused to have their animals sampled: _______________________

Reasons for refusal:

1. ________________________________________________________________________________

2. ________________________________________________________________________________

Total number of animals sampled at this site: ____________________________________________

Number of animals with incomplete data: _______________________________________________

| Missing data or sample | Reason |
| --- | --- |
|  |  |
|  |  |
|  |  |

How hard was it to get the samples at this site today?

1. Very hard 2. Hard 3. Moderate 4. Easy 5. Very easy

Comment: ____________________________________________________________________________________________________________________________________________________________________

- - - - - - - - - - - - - - - - - - - - - - - - - - - - - - - - - - - - - - - - - - - - - - - - - - - - - - - - - - - - - - - - - - - - - - - - - - - - -

Site: _____________________________

Number of animal owners who refused to have their animals sampled: _______________________

Reasons for refusal:

1. ________________________________________________________________________________

2. ________________________________________________________________________________

Total number of animals sampled at this site: ____________________________________________

Number of animals with incomplete data: _______________________________________________

| Missing data or sample | Reason |
| --- | --- |
|  |  |
|  |  |
|  |  |

How hard was it to get the samples from this site today?

1. Very hard 2. Hard 3. Moderate 4. Easy 5. Very easy

Comment:

_______________________________________________________________________________________________________________________________________

**Hospital Visit**

Site: _______________________ Date: ______________________

Time of arrival at site: _________________ Time of departure from site: _________________

Number of ZooLinK staff involved: ________

Number and type of local staff engaged (i.e. paid by project) (Please tick all applicable answers):

Clinical officers

Lab technologists

Other; please specify: _________________________________________________________

Total number of patients sampled: ________

If fewer than 10, why? (Please tick all applicable answers)

Not enough patients

Not enough time

Other; please specify: _________________________________________________________

Number of patients who met inclusion criteria but refused to be sampled: _____________________

Reasons for refusing to be sampled:

1. ________________________________________________________________________________

2. ________________________________________________________________________________

3. ________________________________________________________________________________

Number of patients who accepted to be sampled but refused to give a sample or piece of information: _______________________________________________________________________

| Biological sample refused | Reason |
| --- | --- |
|  |  |
|  |  |
|  |  |
| Piece of information refused | Reason |
|  |  |
|  |  |
|  |  |

How hard was it to get the samples today?

1. Very hard 2. Hard 3. Moderate 4. Easy 5. Very easy
