# Supplementary material for: One Health in Action: Operational Aspects of an Integrated Surveillance System for Zoonoses in Western Kenya
Source: Front Vet Sci. 2019 Jul 31;6:252. doi: 10.3389/fvets.2019.00252 (PMC6684786; doi:10.3389/fvets.2019.00252)
Supplement: Supplementary file 8 [file Table_8.DOCX]

**
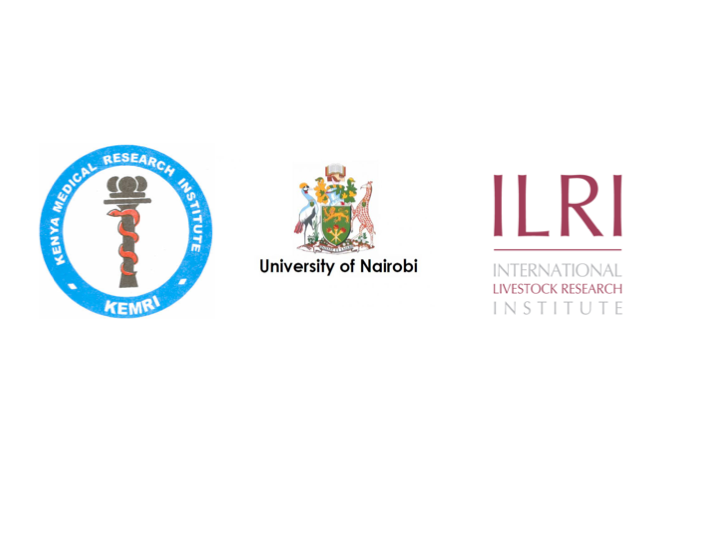
**

| **SOP NO:** **ZOOLINK/BUSIA/2/2017** | **Version: Original** | **Effective date: 1/4/2017** |
| --- | --- | --- |
| **Title: Culture and isolation of *E. coli* – ZooLinK project** | | |
| **Prepared by: Sam Njoroge** | **Sign:** 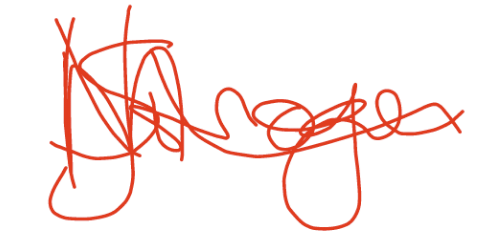 | **Date: 21- Feb-2017** |

1. **PURPOSE / INTRODUCTION:**

The aim of the ZooLinK project is to isolate *Escherichia coli* in animals and humans, and to understand the epidemiology of *E. coli* in Busia, Bungoma and Kakamega Counties.

This SOP describes means and methods needed for the identification by culture of *E. coli* and more.

1. **SCOPE / RESPONSIBILITY:**

This SOP applies to all personnel and persons on attachment who are involved in culture and isolation of *E. coli* - ZooLink project. The section head must ensure that the procedure is strictly followed.

The QA officer should coordinate and supervise the process to ensure all the SOPs are current and up to date.

The technical personnel should prepare, review and update the SOPs related to their work and occasional training for both new and old technical personnel to which the SOP apply.

1. **SAFETY/RISK ASSESSMENT**:

The lab safety measures require that all specimens be considered highly infectious and handled with total care. Some pathogenic strains of *E. coli* produce toxins which when ingested can cause severe diarrhoea and even hospitalization. Care should be observed while handling live cultures.

1. **EQUIPMENT / MATERIALS/ REAGENTS:**

- Petri-dishes with already prepared agar
- Hand gloves
- Clean laboratory coats
- 70% ethanol and JIK in spray bottle
- Biohazard waste bin
- Sterile loops OR metal loops with flame

1. **METHODOLOGY:**

**5.1 Isolation of *E. coli* from faecal material**

**Day 1**

**Buffed peptone water enrichment in broth from human and animal faeces**

- Faecal samples come in 30ml faecal containers transported under cold chain from the field.
- Open the faecal pot and pick approximately 1g of the faecal material with a sterile loop. Ensure that you also touch the mucoid or bloody spots on the faecal material
- Dip the Ig of the faecal matter in 3mL of buffered peptone water. Ensure and even suspension especially for goat and sheep stool.
- Incubate for 18-24 h at 37^0^C

**Day 2**

**Plating on McConkey or EMBA agar**

- Place a barcode on the McConkey or Eosin Methylen Blue agar plate
- Pick an inoculum of the overnight broth and plate it on McConkey or Eosin Methylen Blue agar [Oxoid].
- Incubate the plates for 18-24 h at 37^0^C.

**Day3**

**Picking suspect *E. coli* colonies**

- *E. coli* colonies are pink, round, smooth edged, raised colonies in McConkey. They have a green metallic sheen in EMBA.
- Scan the barcode from the MacConkey or EMBA plate.
- Pick one colony and individually plate on Nutrient Agar or tryptone soy agar.
- Additionally pick other four colonies to have five colonies in total and together plate on plate on Nutrient Agar or tryptone soy agar. Make sure you indicate that this are mixed colonies in the Nutrient Agar or tryptone soy agar plate.

**Day 4**

**Archiving from Nutrient Agar or tryptone soy agar plate**

From the Nutrient Agar or tryptone soy agar, make a emulsion of the *E. coli* colonies in Tryptonse soy broth with 15% glycerol for freezing. Remember to indicate which vial contains the mixed colonies

1. **DOCUMENT CHANGE HISTORY:**

**Version Table:**

| Original:  Title: | Dated:  **1/4/2017** | SOP No.:  **ZOOLINK/BUSIA/1/2017** | No. Pages:  **3** |
| --- | --- | --- | --- |
| Version:  Title: | Dated: | SOP No.: | No. Pages: |
| Version:  Title: | Dated: | SOP No.: | No. Pages: |

**Training Documentation Log for SOP Files**

| Kenya Medical Research Institute  **ZOOLINK/BUSIA/** SOP | | |  | SOP No: **ZOOLINK/BUSIA/2/2017**  Version: **Original**  Effective Date: **1/4/2017** | | |
| --- | --- | --- | --- | --- | --- | --- |
| Title: Culture and isolation of *E. coli* – ZooLink project | | | | | | |
| **NO.** | **DATE** | **NAME** | | | **SIGNATURE** | **TRAINER** |
|  |  |  | | |  |  |
|  |  |  | | |  |  |
|  |  |  | | |  |  |
|  |  |  | | |  |  |
|  |  |  | | |  |  |
|  |  |  | | |  |  |
|  |  |  | | |  |  |
|  |  |  | | |  |  |
|  |  |  | | |  |  |
|  |  |  | | |  |  |
|  |  |  | | |  |  |
|  |  |  | | |  |  |
|  |  |  | | |  |  |
|  |  |  | | |  |  |
|  |  |  | | |  |  |
|  |  |  | | |  |  |
|  |  |  | | |  |  |
|  |  |  | | |  |  |
