# Supplementary material for: One Health in Action: Operational Aspects of an Integrated Surveillance System for Zoonoses in Western Kenya
Source: Front Vet Sci. 2019 Jul 31;6:252. doi: 10.3389/fvets.2019.00252 (PMC6684786; doi:10.3389/fvets.2019.00252)
Supplement: Supplementary file 9 [file Table_9.DOCX]

**
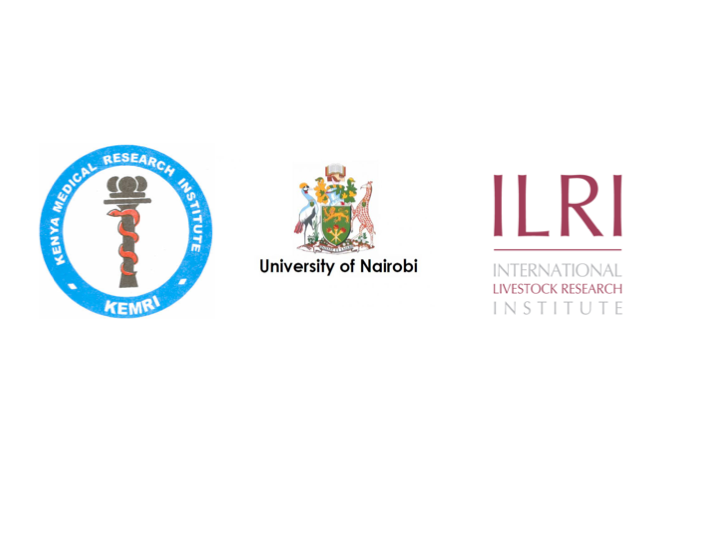
**

| **SOP NO:** **ZOOLINK/BUSIA/3/2017** | **Version: Original** | **Effective date: 1/4/2017** |
| --- | --- | --- |
| **Title: Culture and isolation of *Salmonella spp* – ZooLinK project** | | |
| **Prepared by: Sam Njoroge** | **Sign:** 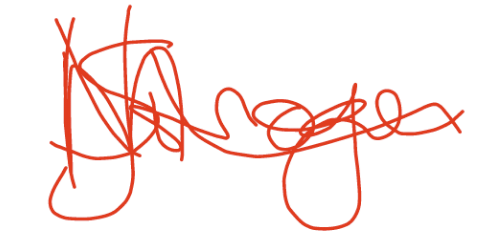 | **Date:21-Feb-2017** |

1. **PURPOSE / INTRODUCTION:**

Some farm animals are infected with *Salmonella* without showing signs of the illness, i.e. they are sub-clinically infected. Feces from these herds may contain *Salmonella* in low numbers. In raw meat, *Salmonella* may also be present in the mesenteric lymph nodes of pigs. To diminish the risk of obtaining false negative results in non-selective pre-enrichment of a feaces, a combination of two selective enrichments and plating on two selective media are performed:

- Selective enrichment in selenite F broth (for human samples) and Rappaport Vassiliadis soy soy peptone broth
- Sub-cultivation on Xylose Lactose Tergitol™ 4 (XLT-4) agar and on Brilliant Green agar (BGA) (or another selective agar media).

**Biochemical confirmation and serotyping of *Salmonella***

Subsequently it is confirmed with biochemical tests whether the colonies resembling *Salmonella* on XLT-4 and BGA are *Salmonella*. Biochemical test are TSI agar, Urea agar (Christensen), and Indole tests in this order [Oxoid].

In addition, the *Salmonella* colonies are serotyped and classified on subspecies level. The biochemical confirmation of *Salmonella* and the serotyping may be performed at the same time.

The aim of the ZooLink project is to isolate *Salmonella spp* in animals and humans, and to understand the epidemiology of *Salmonella spp* in Busia, Bungoma and Kakamega Counties. Prevalence, strains, diversity and AMR profile of Non-Typhoidal Salmonela in human and livestock will be determined in the three Counties. A comparison will be made of results between the three study Counties to assess whether there is any epidemiological explanation for any strain differences in invasive NTS recovered during this project in animal and human populations.

This SOP describes means and methods needed for the identification by culture of *Salmonella spp* and more.

1. **SCOPE / RESPONSIBILITY:**

This SOP applies to all personnel and persons on attachment who are involved in culture and isolation of *Salmonella spp* - ZooLink project. The section head must ensure that the procedure is strictly followed.

The QA officer should coordinate and supervise the process to ensure all the SOPs are current and up to date.

The technical personnel should prepare, review and update the SOPs related to their work and occasional training for both new and old technical personnel to which the SOP apply.

1. **SAFETY/RISK ASSESSMENT**:

Biosafety Level 2 practices should be observed for all the *Salmonella*, except *S*. Typhi. For *S.* Typhi.Carry out all procedures in accordance with local safety codes of practice

1. **EQUIPMENT / MATERIALS/ REAGENTS:**

- • Erlenmeyer flasks (500 ml) etc. sterile (for pre-enrichment)
- • Disposable inoculation loops (1 μl and 10 μl)
- • Plastic petri dishes (9 cm diameter) sterile
- • Balance
- • Incubators at 37^o^C and 41.5^o^C
- • Bunsen burner
- • Pipettes for 0.1 ml (e.g. 1 ml pipettes)
- • Wood spatulas

**5.0 Media**

- • Selenite F broth 7 ml [Oxoid].
- • Rappaport Vassiliadis soy peptone broth 7 ml [Oxoid].
- • Xylose Lysine Desoxycholate (XLT-4) agar plates [Oxoid].
- • Brilliant Green (BGA) agar plates [Oxoid].
- • Nutrient agar plates [Oxoid].

**6.0 Samples**

- • Feaces
- • Pig lymph nodes from slaughterhouse

1. **METHODOLOGY:**

**Day 1: selective pre-enrichment**

Weigh out 3-5 g feces with a sterile wood spatula, put it in 7 ml Rappaport-Vassiliadis soy peptone (RVS) broth (for animal samples) or 7 ml selenite F broth (for human samples).

Stomacher/Homogenise 2-3 lymph nodes with 10ml buffered peptone water and aseptically put 200uL in 7ml of Vassiliadis soy peptone (RVS) broth.

Incubate for 16-20 hours.

**Day 2: Spread on selective agar plates**

Spread a 10 μl loop full from the inoculated and incubated selenite F broth or RVS broth on XLT-4 and on BGA agar plates and incubate at 37^o^C overnight (18-24 hours).

**Day 3: Subcultivation of *Salmonella* suspect colonies**

Read the XLT4 plates:

A typical *Salmonella* colony has a slightly transparent zone of reddish colour and a black centre, a pink-red zone may be seen in the media surrounding the colonies. Mark typical *Salmonella* growth on XLT-4 with a + in the record sheets.

Read the BGA agar plate

Typical *Salmonella* colonies on a BGA agar plate cause the colour of the medium to be red/pink (phenolred is the indicator).

The colonies are grey-reddish/pink and slightly convex.

Mark typical *Salmonella* growth on BGA with a + in the record sheets.

Plate one *Salmonella* suspect colony from XLT-4 and/or BGA agar onto non-selective media, e.g. nutrient agar plates or typtone soy agar plate for biochemical confirmation of *Salmonella* and serotyping

**Day 4-6: Biochemical confirmation and serotyping of *Salmonella***

**Serotyping procedure using Remel^TM^ polyO antisera**

Step 1: Put two separate drops (40 µl each) of 0.85% saline on a glass slide. Emulsify portions of the culture under test with a loop in each drop of 0.85% saline to give a smooth, fairly dense suspension.

Step 2 To one suspension, as a control, add one drop (40 µl) of 0.85% saline and mix. To the other suspension add one drop (40 µl) of undiluted antiserum and mix

Step 3 Rock the slide gently for one minute and observe for agglutination using indirect lighting over a dark background. Discard the used slide for safe disinfection and disposal.

**DOCUMENT CHANGE HISTORY:**

**Version Table:**

| Original:  Title: | Dated:  **1/4/2017** | SOP No.:  **ZOOLINK/BUSIA/3/2017** | No. Pages:  **5** |
| --- | --- | --- | --- |
| Version:  Title: | Dated: | SOP No.: | No. Pages: |
| Version:  Title: | Dated: | SOP No.: | No. Pages: |

**Training Documentation Log for SOP Files**

| Kenya Medical Research Institute  **ZOOLINK/BUSIA/** SOP | | |  | SOP No: **ZOOLINK/BUSIA/3/2017**  Version: **Original**  Effective Date: **1/4/2017** | | |
| --- | --- | --- | --- | --- | --- | --- |
| Title: Culture and isolation of *Salmonella spp* – ZooLink project | | | | | | |
| **NO.** | **DATE** | **NAME** | | | **SIGNATURE** | **TRAINER** |
|  |  |  | | |  |  |
|  |  |  | | |  |  |
|  |  |  | | |  |  |
|  |  |  | | |  |  |
|  |  |  | | |  |  |
|  |  |  | | |  |  |
|  |  |  | | |  |  |
|  |  |  | | |  |  |
|  |  |  | | |  |  |
|  |  |  | | |  |  |
|  |  |  | | |  |  |
|  |  |  | | |  |  |
|  |  |  | | |  |  |
|  |  |  | | |  |  |
|  |  |  | | |  |  |
|  |  |  | | |  |  |
|  |  |  | | |  |  |
|  |  |  | | |  |  |
|  |  |  | | |  |  |
|  |  |  | | |  |  |
