# Supplementary material for: One Health in Action: Operational Aspects of an Integrated Surveillance System for Zoonoses in Western Kenya
Source: Front Vet Sci. 2019 Jul 31;6:252. doi: 10.3389/fvets.2019.00252 (PMC6684786; doi:10.3389/fvets.2019.00252)
Supplement: Supplementary file 10 [file Table_10.DOCX]

**
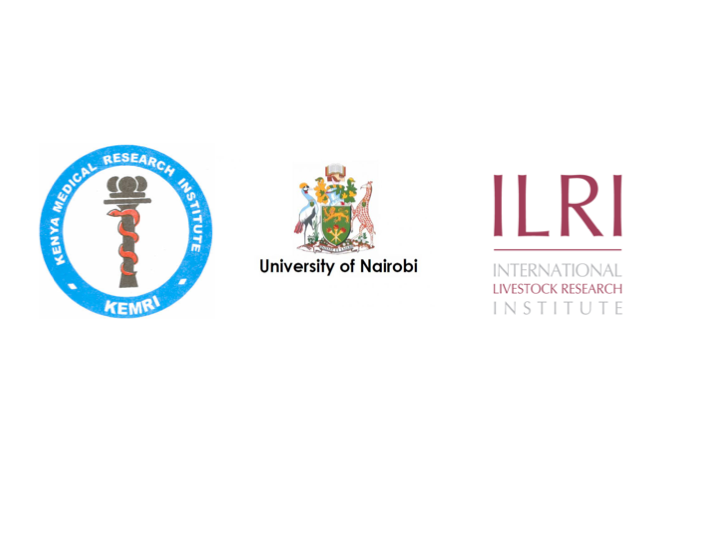
**

| **SOP NO:** **ZOOLINK/BUSIA/1/2017** | **Version: Original** | **Effective date: 1/4/2017** |
| --- | --- | --- |
| **Title: Campylobacter culture and PCR – ZooLink project** | | |
| **Prepared by: Sam Njoroge** | **Sign:** 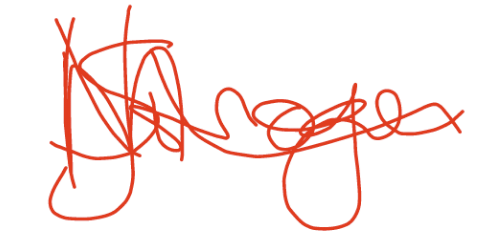 | **Date:21-Feb-2017** |

**PURPOSE / INTRODUCTION:**

The aim of the ZooLink project is to determine the prevalence and diversity of *Campylobacter* species in animals and humans, and to understand the epidemiology of *Campylobacter* in the Busia, Bungoma and Kakamega Counties.

This SOP describes means and methods needed for culture and identification by molecular biology of Campylobacter spp, and more specifically *C. jejuni* and *C. coli*. All positive culture on Blood Agar after anaerobic incubation must be tested by PCR.

1. **SCOPE / RESPONSIBILITY:**

This SOP applies to all personnel and persons on attachment who are involved in Molecular Biology in the Campy - ZooLink project. The section head must ensure that the procedure is strictly followed.

The QA officer should coordinate and supervise the process to ensure all the SOPs are current and up to date.

The technical personnel should prepare, review and update the SOPs related to their work and occasional training for both new and old technical personnel to which the SOP apply.

1. **SAFETY/RISK ASSESSMENT**:

The lab safety measures require that all specimens be considered highly infectious and handled with total care. The transilluminator produces dangerous levels of ultraviolet (UV) light. Although the system has safety interlocks to prevent exposure, always take care to ensure that the system is working properly. Failure to do so could result in exposure to UV light. Do not look directly at the UV light. Use a UV-protecting face shield or stay behind the uv-protective transilluminator shield.

1. **EQUIPMENT / MATERIALS/ REAGENTS:**

- Exeter broth
- Blood agar [Oxoid]
- mCCDA [Oxoid]
- 15ml glass test tubes or Plastic bijous
- Pipettes and tips: 10µL, 100µL, 1000µL
- Disposable loops
- Eppendorf tube 1.5 ml
- Centrifuge
- Heating block
- Hand gloves
- Clean laboratory coats
- 70% ethanol and JIK in spray bottle
- Biohazard waste bin
- Chelex-100
- TE buffer (10mM Tris-HCl, 1mM EDTA pH 8.0)
- Sterile distilled water
- 2% agarose gel (Appendix 1)
- Primers diluted to a stock solution of 50 pmol (C412F, CampR2, CjejlpxAF, CjejlpxAR, CcollpxAF)
- Illustra PureTaq Ready-To-Go^TM^ PCR Beads

1. **METHODOLOGY:**

**4.1 Culture**

**4.1.1 Making of media**

***Procedure***

1. Sterilise one litre of Oxoid Nutrient Broth CMB0001B, cool to 50^O^C and hold at this temperature. For the preparation of agar, add 13g/L of the agar to the broth before sterilisation and supplement as above.
2. Reconstitute the contents of one vial of campylobacter growth selectavial (FBP, SV61) and one vial of campylobacter enrichment selectavial (EXETER, SV59) using an appropriate diluent. The best method is to aseptically add the diluent using a sterile needle and syringe. Draw the diluent into a 10ml syringe and after removing her plastic cap, inject through the rubber stopper of the vial. The lyophilised supplement will rapidly dissolve and may be withdrawn into the syringe.
3. Aseptically add 5%v/v lysed horse blood into the nutrient broth and mix.
4. Put the broth in sterile falcon tubes to be almost full with minimal head space to allow anaerobic conditions.

**Culture of *Campylobacter spp* using mCCDA**

***Directions***Suspend 22.75g of Campylobacter Blood-Free Selective Agar Base in 500ml of distilled water and bring to the boil to dissolve. Sterilize by autoclaving at 121°C for 15 minutes. Cool to 50°C. Aseptically add 1 vial of CCDA Selective Supplement SR0155 reconstituted as directed. Mix well and pour into sterile Petri dishes.

***Description***Modified CCDA Medium is based on the original formulation described by Bolton *et al.,*1987, which was developed to replace blood with charcoal, ferrous sulphate and sodium pyruvate. Improved selectivity was achieved when cephazolin in the original formulation was replaced by cefoperazone as the selective agent^2^. More recent work has shown an increased isolation rate can be achieved if the plates are incubated at 37°C rather than 42°C.

Amphotericin B has been added to the formula to suppress the growth of yeast and fungal contaminants that may occur at 37°C.

Modified CCDA medium and Campy-BAP medium were equal in performance in a rapid colony-lift procedure for detection of thermophilic *campylobacters* in which membranes are blotted on agar cultures and then subjected to immunoassay.

In a study of healthy puppies and kittens for carriage of *Campylobacter* species, modified CCDA medium was found to be a suitable medium and more productive for *Campylobacter upsaliensis* in this application than CAT medium. Modified CCDA medium has been confirmed as suitable for isolation of *Campylobacter*spp. from non-clinical samples following enrichment in Exeter broth.

The use of Campylobacter Blood-Free Medium is specified by the U.K. Ministry of Agriculture, Fisheries and Food (MAFF) in a validated method for isolation of *Campylobacter* from foods.

**4.1.2 Cultures**

***Day 1: Enrichment and platting procedure***

- Fill Exeter broth in glass tube to the three quarters mark
- Put approximately 2g of feaces, or 200mL of homogenized fecal sample to the almost full mark.
- Incubate for 18-24 hours at 42^O^C

***Day 2: Platting***

- Aseptically place 0.45 Micron filter on a clean barcoded mCCDA plate
- Invert the exter tube once and pour 100μL on top of the filter and leave for 30 minutes for the fluid to pass through. Campylobacter spp are able to pass through the filter while other bacteria are not.
- Incubate the mCCDA plates at 42^O^C after inserting one sachet of campygen in a 3.5liter jar for 48 hours.

***Day 4: Reading mCCDA plate***

- Observe for creamy-grey or grey colonies and record them as suspects for follow-up or additional test. Streak positive colonies in blood agar plates and Incubate for 18-24 hours at 42^O^C

***Day 5: Archiving and preparation for PCR***

The colony morphology of campylobacters can be used as a guideline for identification to species level. *Campylobacter jejuni* strains produce grey, moist flat spreading colonies. Some strains may have a green hue or a dry appearance, with or without a metallic sheen. *Campylobacter coli* strains tend to be creamy-grey in colour, moist, slightly raised and often produce discrete colonies.

Colonies tend to swarm when initially isolated from clinical specimens.

***Storage/Archival of Positive samples***

Positive samples should be first plated on Blood Agar and stocking of grown isolates done after a minimum of 18 hours. Strains are stored in microbank tubes awaiting PCR-species differentiation.

- 1. **PCR**

**4.2.1 Bacterial DNA Extraction using Chelex-100^1^**

1. Make a solution of 20% (w/v) Chelex-100 in 10mM Tris-HCl, 1mM EDTA, pH8.0 (20g Chelex in 100ml TE Buffer). Be aware when pipetting the Chelex solution that the Chelex particles settle quickly, so shake the bottle after every three aliquots
2. Suspend 1 loopful of bacterial cells from Blood Agar plates in 300µl Chelex solution in a 1.7ml Eppendorf.
3. Heat the Eppendorf at 95°C for 10 minutes.
4. Centrifuge the Eppendorf at 10,000 RPM for 2 minutes.
5. Remove 50µl of the clear supernatant and add to a new Eppendorf containing 450µl sterile distilled water.
6. Store the DNA extract for short-term storage at 2–8°C, for long-term storage at –80°C.

**4.2.2 LINTON PCR**

The aim of this PCR protocol is to identify *Campylobacter spp* ^2^

1. Primers (diluted to a stock solution of 50 pmol)

| Forward | Sequence | Reverse | Sequence | Amplicon Size |
| --- | --- | --- | --- | --- |
| C412F | GGA TGA CAC TTT TCG GAG C | CampR2 | GGC TTC ATG CTC TCG AGT T | 857 bp |

1. Reaction Conditions

94°C for 5 minutes

94°C for 1 minute

58°C for 1 minute 25 cycles

72°C for 1 minute

72°C for 7 minutes

1. Method
2. Add reagents to master mix Illustra PureTaq Ready-To-Go^TM^ PCR Beads as below:

| Reagent | Calculated 25µL final volume for one sample | × (N) number of samples |
| --- | --- | --- |
| RNA/DNA free Water | 22µL |  |
| Primer F | 0.5µL |  |
| Primer R | 0.5µL |  |
| DNA Extract | 2µL |  |

1. Perform PCR reaction as stated above.
2. Run products on 2% agarose gel (120V for 40-70 minutes). The length of time you run the gel is dependent on the size of the gel.

**4.2.3 LPX PCR**

The aim of this PCR protocol is to identify *Campylobacter jejuni* and *coli*^3^

1. Primers

| Forward | Sequence | Reverse | Sequence | Amplicon  Size |
| --- | --- | --- | --- | --- |
| CjejlpxAF | ACA ACT TGG TGA CGA TGT TGT A | CjejlpxAR | CAA TCA TGD GCD ATA TGA SAA TAH GCC AT | 331 |
| CcollpxAF | AGA CAA ATA AGA GAG AAT CAG |  |  | 391 |

1. Reaction Conditions

94°C for 5 minutes

94°C for 1 minute

50°C for 1 minute 30 cycles

72°C for 1 minute

72°C for 10 minutes

1. Method
2. Add reagents to master mix Illustra PureTaq Ready-To-Go^TM^ PCR Beads as below:

| Reagent | Calculated 25µL final volume for one sample | × (N) number of samples |
| --- | --- | --- |
| RNA/DNA free Water | 21.5 µL |  |
| Primer CjejlpxAF | 0.5 µL |  |
| Primer CcollpxAF | 0.5µL |  |
| Primer CjejlpxAR | 0.5 µL |  |
| DNA Extract | 2µL |  |

1. Perform PCR reaction as stated above.
2. Run products on 2% agarose gel (120V for 40-70 minutes). The length of time you run the gel is dependent on the size of the gel.
3. **APPENDIX: Preparation of 2% agarose gel**
4. Materials/Reagents

- AGTC Bioproducts Hi-Res Agarose (Order No: AGD1 500gm)
- 0.5X TBE buffer
- Microwave
- 300ml Conical Flask/Erlenmeyer Flask
- Electrophoresis gel setting tray
- Combs
- Syber green/ Ethidium bromide

1. Methods
2. Wearing gloves, weigh 2.0 grams of Hi-Res Agarose.
3. Measure 100ml of 0.5X TBE.
4. Boil using microwave for 2 minutes until the agraose dissolves completely and no visible flecks are seen. **CAUTION:** Wear heat resistant gloves while handling the hot flask
5. Cool at 50^0^C for 30 minutes.
6. Add 5 µL of Ethidium bromide or 20 µL of Syber green. **CAUTION:** Ethidium Bromide is a powerful mutagen. Always use gloves when handling the agarose gels. Dispose of the pipette tip in a chemical waste container designated for ethidium bromide waste. Gloves in contact with ethidium bromide must be disposed in ethidium bromide waste container.
7. Pour on electrophoresis gel setting tray, place comb and let it set for 30-40 minutes.
8. Carefully remove the combs (wash these and replace in the drawer), ensuring that the wells are not damaged. Remove the gel from the casting setup and place it in the gel box. Make sure that the wells are at the end where the black electrodes are. You will run toward the red
9. Flood with 0.5x buffer until gel is submerged.
10. Load the first well with DNA marker/ladder and the other respective wells with pcr products.
11. Place the top on the tank ensuring that the electrodes are located correctly and the positive is connected to positive etc. Connect the leads to the voltage regulator/powerpack again checking for correct polarity, then turn on the powerpack and set the voltage at an appropriate value (usually 130 volts).
12. Run at 130V for 40-45 minutes. Check to ensure that electrophoresis is occurring (bubbles will be generated at the electrodes).
13. Observe gel Under Ultra-Violet light using Gel-Max Imager. Place it on the glass surface so that the area containing your samples is in the centre. Close the door and switch on the transilluminator and camera. Alter the exposure, focus and zoom to obtain a clear image of the correct size and brightness. Capture the image and save the picture. **CAUTION:** Ultra-Violet light is harmful to the naked eye, wear U.V protected goggles if the gel visualization is not done inside a hood.
14. **APPENDIX: Preparation of 0.5X TBE Buffer**

5X TBE (1.1M Tris; 900mM Borate; 25mM EDTA; pH 8.3)

| REAGENT | Weight in grams for 1 Litre | Sigma Aldrich Cat# |
| --- | --- | --- |
| EDTA | 4.69g | E-5134 |
| Trizma Base | 54g | T-1503 |
| Boric Acid | 27.5g | B-0252 |

In an appropriately sized beaker, dissolve Tris, Boric Acid and EDTA in a volue of distilled deionized water that is equal to about 75% of the volume of 5X buffer to be made. Mix completely. EDTA may be slow to dissove, but low heat can be applied to facilitate dissolution. Once dissolved, add water to just below the desired volume. pH must then be adjusted to 8.3 by adding concentrated HCl, using a pH meter to constantly monitor the pH. There is no need to sterilize the solution and it can be stored at room temperature. Precipitation will form over time, but buffer is gererally still usable.

0.5X TBE Electrophoresis Buffer for 1L

| 5X TBE | 100 mL |
| --- | --- |
| Distilled, deionized water | 900 mL |

In an erlynmeyer flask, add 100mL of 5X TBE to 900mL of distilled deionized water.

Mix well and use.

**NOTE:** 5X TBE buffer is premade. You will only be required to dilute the 5X buffer to 0.5X for use.

1. **REFERENCES:**

1. Walsh, Metzger and Higuchi (1991*) Biotechniques* 10:506-513

2. Katzav, M., et al., *PCR assay for the detection of Campylobacter in marinated and non-marinated poultry products.* Food Microbiology, 2008. 25(7): p. 908-914.

3. Klena, J. D., C. T. Parker, et al. (2004). Differentiation of Campylobacter coli, Campylobacter jejuni, Campylobacter lari, and Campylobacter upsaliensis by a multiplex PCR developed from the nucleotide sequence of the lipid a gene lpxA. Journal of Clinical Microbiology **42**(12): 5549-5557.

1. **DOCUMENT CHANGE HISTORY:**

**Version Table:**

| Original:  Title: | Dated:  **1/4/2017** | SOP No.:  **ZOOLINK/BUSIA/1/2017** | No. Pages:  **9** |
| --- | --- | --- | --- |
| Version:  Title: | Dated: | SOP No.: | No. Pages: |
| Version:  Title: | Dated: | SOP No.: | No. Pages: |

**Training Documentation Log for SOP Files**

| Kenya Medical Research Institute  **ZOOLINK/BUSIA/** SOP | | |  | SOP No: **ZOOLINK/BUSIA/1/2017**  Version: **Original**  Effective Date: **1/4/2017** | | |
| --- | --- | --- | --- | --- | --- | --- |
| Title: Campylobacter culture and PCR – ZooLink project | | | | | | |
| **NO.** | **DATE** | **NAME** | | | **SIGNATURE** | **TRAINER** |
|  |  |  | | |  |  |
|  |  |  | | |  |  |
|  |  |  | | |  |  |
|  |  |  | | |  |  |
|  |  |  | | |  |  |
|  |  |  | | |  |  |
|  |  |  | | |  |  |
|  |  |  | | |  |  |
|  |  |  | | |  |  |
|  |  |  | | |  |  |
|  |  |  | | |  |  |
|  |  |  | | |  |  |
|  |  |  | | |  |  |
|  |  |  | | |  |  |
|  |  |  | | |  |  |
|  |  |  | | |  |  |
|  |  |  | | |  |  |
|  |  |  | | |  |  |
|  |  |  | | |  |  |
|  |  |  | | |  |  |
