# Supplementary material for: One Health in Action: Operational Aspects of an Integrated Surveillance System for Zoonoses in Western Kenya
Source: Front Vet Sci. 2019 Jul 31;6:252. doi: 10.3389/fvets.2019.00252 (PMC6684786; doi:10.3389/fvets.2019.00252)
Supplement: Supplementary file 11 [file Table_11.DOCX]

**
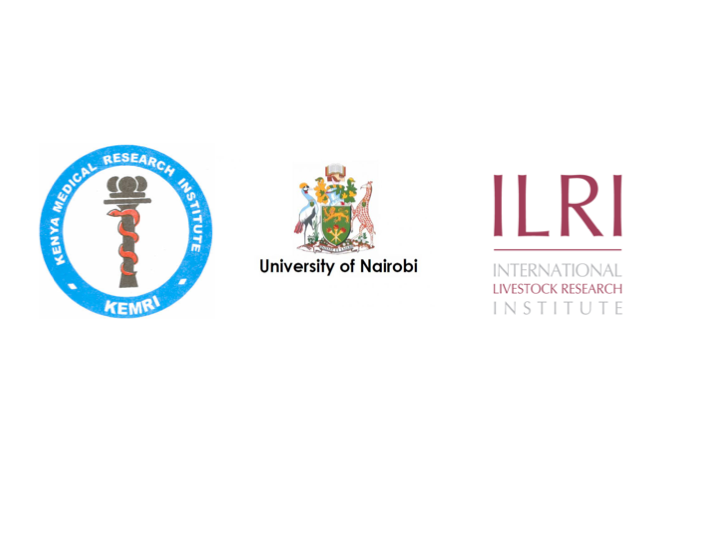
**

| **SOP NO:** **ZOOLINK/BUSIA/4/2017** | **Version: Original** | **Effective date: 1/4/2017** |
| --- | --- | --- |
| **Title: Culture and isolation of *Staphylococcus aureus* – ZooLink project** | | |
| **Prepared by: Sam Njoroge** | **Sign:** 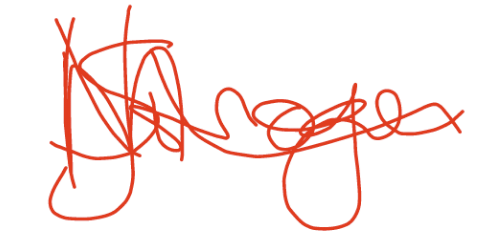 | **Date:21-Feb-2017** |

1. **PURPOSE / INTRODUCTION:**

Some farm animals are colonized with *Staphylococcus aureus* in their nares. *Staphylococcus aureus* pose a threat to humans as a source of especially S. aureus resistant to methicillin.

The aim of the ZooLink project is to isolate *Staphylococcus aureus* in animal and human nares, and to understand the epidemiology of Methicillin Resistant *Staphylococcus aureus* (MRSA) in Busia, Bungoma and Kakamega Counties.

This SOP describes means and methods needed for the identification by culture of *Staphylococcus aureus* and more.

1. **SCOPE / RESPONSIBILITY:**

This SOP applies to all personnel and persons on attachment who are involved in culture and isolation of *Staphylococcus aureus* - ZooLink project. The section head must ensure that the procedure is strictly followed.

The QA officer should coordinate and supervise the process to ensure all the SOPs are current and up to date.

The technical personnel should prepare, review and update the SOPs related to their work and occasional training for both new and old technical personnel to which the SOP apply.

1. **SAFETY/RISK ASSESSMENT**:

Biosafety Level 2 practices should be observed for all the *Staphylococcus aureus*. Carry out all procedures in accordance with local safety codes of practice

1. **EQUIPMENT / MATERIALS/ REAGENTS:**

- • Erlenmeyer flasks (500 ml) etc. sterile (for pre-enrichment)
- • Disposable inoculation loops (1 μl and 10 μl)
- • Plastic petri dishes (9 cm diameter) sterile
- • Balance
- • Incubators at 37^o^C and 41.5^o^C
- • Bunsen burner
- • Pipettes for 0.1 ml (e.g. 1 ml pipettes)
- • Wood spatulas

**5.0 Media**

- • E-swabs with amies transport media [481CE] for human sampling and Purflock swabs [MW176PF] for animal sampling.
- Tryptone soya broth 3 ml [Oxoid].
- • Mannitol salt agar plates [Oxoid].
- • Oxacillin Resistance Screening agar plates with OSRAB supplement [Oxoid].
- • Nutrient agar plates [Oxoid].

**6.0 Samples**

- •Nasal swab samples

1. **METHODOLOGY:**

**Day 1: Non-selective pre-enrichment**

Put nasal swab already containing inoculum into 3ml tryptone soya broth. Mix. Incubate at 37^o^C overnight (16-20 hours).

**Day 2: Spread on selective agar plates**

Spread a 10 μl loop full from the inoculated and incubated tryptone soya broth on mannitol salt agar plates and incubate at 37^o^C overnight (18-24 hours).

**Day 3: Subcultivation of *Staphylococcus aureus s*uspect colonies**

Read the Mannitol plates:

A typical *Staphylococcus aureus* colony is small, raised and yellow in colour. Mark typical *Staphylococcus spp* growth on Mannitol salt with a + in the record sheets.

Pick one yellow colony and streak onto non-selective media, e.g. nutrient agar plates for biochemical confirmation of *Staphylococcus aureus* and serotyping.

**Day 4: Biochemical confirmation and serotyping of *Staphylococcus aureus***

| Using StaphTEX™ Blue latex agglutination test, emulsify a small portion of the yellow colony now grown in nutrient agar plates onto a white card and check for agglutination.  Clumping of blue latex particles indicate test organism is coagulase positive for *Staphylococcus aureus.* Record results  **Day 5: Plating on Oxacillin resistance screening agar**  Plate suspect colony on Oxacillin resistance screening agar for 18-24 hours at 37^O^C  **Day 6: Read results and archive**  Blue colonies are indicative of Methicillin Resistant *Staphylococcus aureus.*  From the Nutrient Agar or tryptone soy agar, make a emulsion of the *Staphylococcus aureus* colonies in Tryptonse soy broth with 15% glycerol for freezing. Remember to indicate which vial is MRSA positive. |
| --- |

**DOCUMENT CHANGE HISTORY:**

**Version Table:**

| Original:  Title: | Dated:  **1/4/2017** | SOP No.:  **ZOOLINK/BUSIA/4/2017** | No. Pages:  **4** |
| --- | --- | --- | --- |
| Version:  Title: | Dated: | SOP No.: | No. Pages: |
| Version:  Title: | Dated: | SOP No.: | No. Pages: |

**Training Documentation Log for SOP Files**

| Kenya Medical Research Institute  **ZOOLINK/BUSIA/** SOP | | |  | SOP No: **ZOOLINK/BUSIA/4/2017**  Version: **Original**  Effective Date: **1/4/2017** | | |
| --- | --- | --- | --- | --- | --- | --- |
| Title: Culture and isolation of *Staphylococcus aureus* – ZooLink project | | | | | | |
| **NO.** | **DATE** | **NAME** | | | **SIGNATURE** | **TRAINER** |
|  |  |  | | |  |  |
|  |  |  | | |  |  |
|  |  |  | | |  |  |
|  |  |  | | |  |  |
|  |  |  | | |  |  |
|  |  |  | | |  |  |
|  |  |  | | |  |  |
|  |  |  | | |  |  |
|  |  |  | | |  |  |
|  |  |  | | |  |  |
|  |  |  | | |  |  |
|  |  |  | | |  |  |
|  |  |  | | |  |  |
|  |  |  | | |  |  |
|  |  |  | | |  |  |
|  |  |  | | |  |  |
