# Supplementary material for: One Health in Action: Operational Aspects of an Integrated Surveillance System for Zoonoses in Western Kenya
Source: Front Vet Sci. 2019 Jul 31;6:252. doi: 10.3389/fvets.2019.00252 (PMC6684786; doi:10.3389/fvets.2019.00252)
Supplement: Supplementary file 12 [file Table_12.DOCX]

**
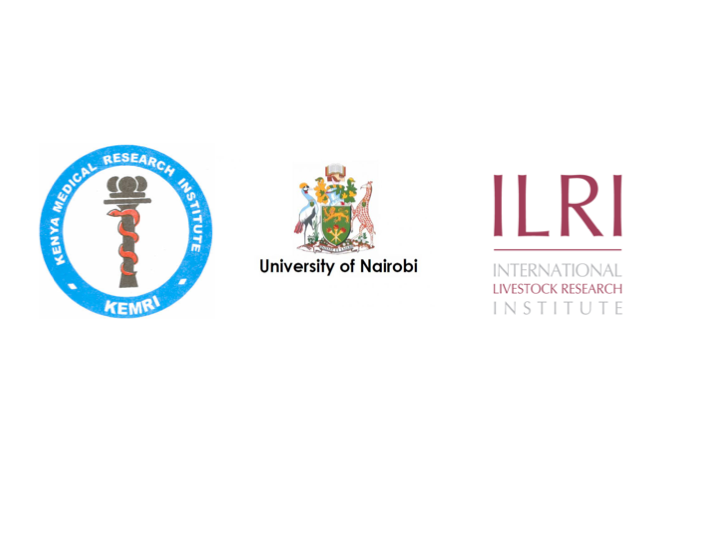
**

| **SOP NO:** **ZOOLINK/BUSIA/RBT/2017** | **Version: Original** | **Effective date: 1/4/2017** |
| --- | --- | --- |
| **Title: Rose Bengal Test – ZooLink project** | | |
| **Prepared by: Sam Njoroge** | **Sign:** 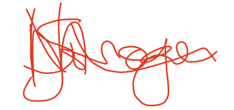 | **Date:21-Feb-2017** |

1. **PURPOSE / INTRODUCTION:**

Humans and animals maybe infected with Brucella.

The aim of the ZooLink project is to screen for Brucella in animal and human sera, and to assess the sero-prevalemce of Brucella in Busia, Bungoma and Kakamega Counties using the Rose Bengal Test.

This SOP describes means and methods needed for the screening of Brucella using the Rose Bengal Test.

1. **SCOPE / RESPONSIBILITY:**

This SOP applies to all personnel and persons on attachment who are involved screening of Brucella using the Rose Bengal Test - ZooLink project. The section head must ensure that the procedure is strictly followed.

The QA officer should coordinate and supervise the process to ensure all the SOPs are current and up to date.

The technical personnel should prepare, review and update the SOPs related to their work and occasional training for both new and old technical personnel to which the SOP apply.

1. **SAFETY/RISK ASSESSMENT**:

Biosafety Level 2 practices should be observed when handling sera. Carry out all procedures in accordance with local safety codes of practice

1. **EQUIPMENT / MATERIALS/ REAGENTS:**

- • plastic card with white background
- • Pipettes for 25μL (e.g. 100μL pipettes)
- • white light source
- • disposable pipette tips

**5.0 Reagent**

- • Rose Bengal Reagent supplied by Universidad de Navarra, department of microbiology.

**6.0 Samples**

- •Serum samples form animals and humans

1. **METHODOLOGY:**
2. Bring the test reagents and samples to room temperature.
3. Re-suspend the RBT reagent bottle gently to obtain a consistent mixture. Aspirate with a pipette several times to obtain a thorough mixing.
4. Take out the test sera from the freezer which should be frozen for at least a week to thaw and attain room temperature.
5. Place 1 drop (25 μL) of the RBT reagent and mix with an equal amount of serum (25 μL) under test into one of the circles on the test card and mix using a disposable stirrer. **Note:** Do the same with 1 drop of positive control serum and 1 drop of negative control serum into two additional circles on the test cards. Mix the contents of each circle while spreading over the entire area enclosed by the ring. Use separate stirrers for each mixture
6. Rotate the slide slowly either by hand or by means of a mechanical rotator (100 r.p.m.) for a period of **2 minutes**.
7. Observe immediately under a suitable light source for any degree of agglutination.

**DOCUMENT CHANGE HISTORY:**

**Version Table:**

| Original:  Title: | Dated:  **1/4/2017** | SOP No.:  **ZOOLINK/BUSIA/RBT/2017** | No. Pages:  **3** |
| --- | --- | --- | --- |
| Version:  Title: | Dated: | SOP No.: | No. Pages: |
| Version:  Title: | Dated: | SOP No.: | No. Pages: |

**Training Documentation Log for SOP Files**

| Kenya Medical Research Institute  **ZOOLINK/BUSIA/** SOP | | |  | SOP No: **ZOOLINK/BUSIA/RBT/2017**  Version: **Original**  Effective Date: **1/4/2017** | | |
| --- | --- | --- | --- | --- | --- | --- |
| Rose Bengal Test – ZooLink project | | | | | | |
| **NO.** | **DATE** | **NAME** | | | **SIGNATURE** | **TRAINER** |
|  |  |  | | |  |  |
|  |  |  | | |  |  |
|  |  |  | | |  |  |
|  |  |  | | |  |  |
|  |  |  | | |  |  |
|  |  |  | | |  |  |
|  |  |  | | |  |  |
|  |  |  | | |  |  |
|  |  |  | | |  |  |
|  |  |  | | |  |  |
|  |  |  | | |  |  |
|  |  |  | | |  |  |
|  |  |  | | |  |  |
|  |  |  | | |  |  |
