# Supplementary material for: One Health in Action: Operational Aspects of an Integrated Surveillance System for Zoonoses in Western Kenya
Source: Front Vet Sci. 2019 Jul 31;6:252. doi: 10.3389/fvets.2019.00252 (PMC6684786; doi:10.3389/fvets.2019.00252)
Supplement: Supplementary file 13 [file Table_13.pdf]

Detection of viable metacestodes of Taenia spp. in human, porcine and bovine serum samples with the use of a monoclonal antibody-based sandwich ELISA

Department/Unit: BMW/ VHU

Table of contents

General information
Definitions and abbreviations
Method
Quality control
Reporting and archiving
References
Revision
Approval

1 General information

- Background information

Monoclonal antibody-based antigen detection uses two monoclonal antibodies to "trap" the antigen circulating in blood, CSF or urine[1] (Correa et al., 1989; Harrison et al., 1989; Choromanski et al., 1990; Brandt et al., 1992; Wang et al., 1992; Garcìa et al., 1998, 2000; Erhart et al., 2002; Castillo et al., 2009). These assays give a positive result when viable, well-established cysts are present.

The monoclonal antibodies used in the test developed by Brandt et al. (1992) and modified by Van Kerckhoven et al. (1998) and Dorny et al. (2000) were originally prepared against antigens from T. saginata, but can not only detect viable cysticerci in bovines, but also cysticerci of T. solium in pigs and humans. The cross-reactions between antigens produced by T. solium and T. saginata metacestodes are a welcome advantage here. There are, unfortunately, also cross-reactions with antigens from T. hydatigena metacestodes (Dorny et al., 2004) and T. s. asiatica metacestodes (Fall et al., 1996; Geerts et al., 1992) in pigs. Therefore, in regions where these parasites are endemic, the detection of T. solium cysticercosis is restricted (Dorny et al., 2001) and accurate data on the prevalence of porcine cysticercosis are not easily available or are of questionable reliability (Rajshekhar et al., 2003).

1 The test used for antigen detection in urine is a slightly modified test!

- General principle of the test

To use this assay (see Figure), a purified antibody (the "capture" antibody) is bound to a solid phase (a polystyrene plate). Antigen is then added and allowed to complex with the bound antibody. Unbound products are then washed away; a labeled second antibody (the "detection" antibody) is allowed to bind to the antigen, thus completing the "sandwich". The assay is then quantified by measuring the amount of labeled antibody. The intensity of the color can be measured (OD). The obtained OD's are then processed statistically to

determine whether a sample is positive or not. This interpretation is based on a set of known positive and negative reference samples.

- First (capturing) antibody   B158C11A10
- Blocking
- Serum (with Ag)
- Second (detecting) antibody   B60H8A4
- Streptavidine labelled peroxidase
- OPD substrate

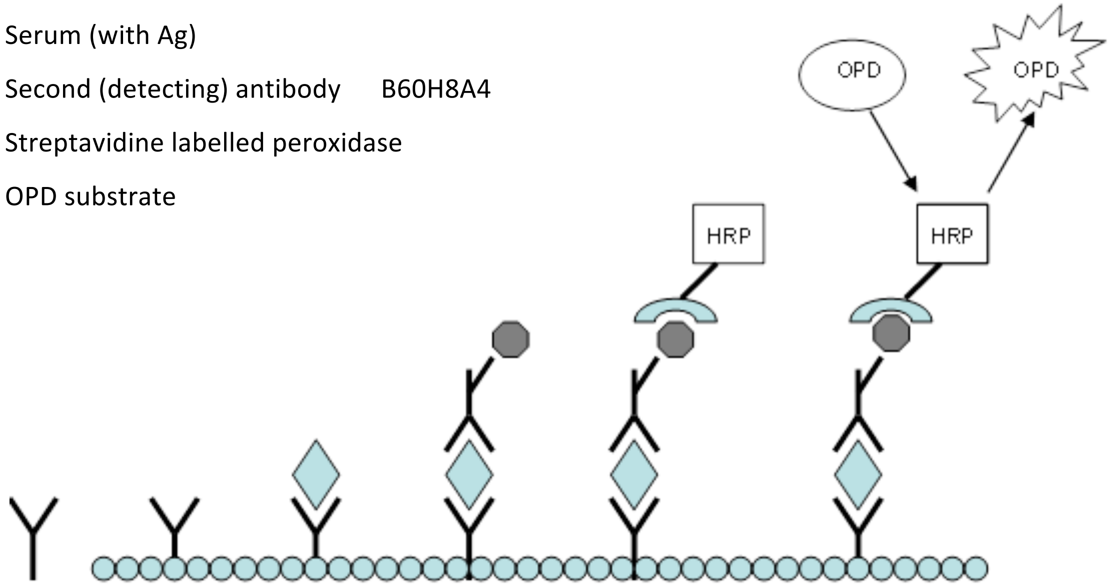

Figure 1: *The general principle of the sandwich ELISA*

1.1 Reagents

- Reference codes and suppliers

Table1 gives you a list of the products used in the ELISA assay. For accurate pricing and availability, please contact the company/supplier.

Please note that the information in this table can change without prior notice!

Table 1*: Product name, quantity, company/supplier name and product reference code of the products needed to do the ELISA.*

**Product**	**Quantity**	**Company or supplier name**	**Reference code**
Biotin Protein Labeling Kit	5 reactions	Roche	1418165
Carbonate-Bicarbonate Buffer tablets	50 capsules	Sigma-Aldrich	C3041
H2O2 30% P.A. ISO	250 ml	VWR	1.072.090.250
Immunoplate Maxisorp F96	60 plates	VWR	NUNC439454
Monoclonal antibodies	for 10 plates	please contact VHU	
Na2CO3 powder	1 kg	VWR	1.063.921.000
NAHCO3 powder	1 kg	VWR	1.063.291.000

Newborn Bovine Serum (NBCS)	100 ml	Invitrogen	16010-167
OPD tablets	100 tablets	Thermo scientific	34006
Phosphate-citrate buffer	50 tablets	Sigma-Aldrich	P-4809
PBS tablets	100 tablets	OXOID	BR0014G
Streptavidin-HRP (Jackson Immunoresearch)	1 mg/vial	Lucron	016-030-0840
TCA ≥ 99.0% (titration)	500 g	Sigma-Aldrich	T4885-500g
TITRISOL H2SO4 (0.5 mol/l)	250 ml	VWR	1.099.810.001
Tween 20	100 ml	Sigma-Aldrich	P1379-100ml

- Monoclonal antibodies

The monoclonal antibodies are developed, produced and labeled by the Veterinary Helmintholgy Unit (VHU). The production procedure is described in general by Harlow and Lane (1988).

• The capturing antibody

The capturing antibody (B158C11A10) is used at 5 µg/ml coating buffer (pH 9.6). The quantity of monoclonal you must take to have 5 µg depends on the batch of monoclonal you are using.

We recommend that the capturing antibody be stored at -20°C.

• The detecting antibody

The detecting antibody (B60H8A4) is labeled to biotin and is used at 1.25 µg/ml blocking buffer. The quantity of monoclonal you must take to have 1.25 µg depends on the batch of monoclonal you are using. We recommend that you store the antibody at +4°C. Following the manufacturer's instructions, we added 1% bovine serum albumin.

- Buffers and product preparations

1. Phosphate Buffered Saline (PBS)

The PBS buffer is prepared using tablets.
One tablet in 100 ml of RO-DI water yields a 100 ml PBS buffer, pH 7.3.

2. Trichloroacetic acid (TCA)

The solution used for the "pretreatment" of the serum samples is a 5% (W/V) solution in RO-DI water.
Example: dissolve 0.5 g of TCA crystals in 10 ml RO-DI water.

3. Washing buffer

The washing buffer consists of PBS with 0.05% (V/V) Tween 20.
Example 1: 1 liter of PBS + 0.5 ml of Tween 20.
Example 2: 1 liter of PBS + 0.555 g of Tween 20 (density is 1.11)

4. Blocking buffer

The blocking buffer consists of washing buffer + 1% (V/V) of Newborn Calf Serum (NBCS).
Example: 49.5 ml of PBS-Tween 20 + 0.5 ml of NBCS
Note that the NBCS has to be heat inactivated before use. To do this, you must put the serum at 56°C for 30 minutes (in a waterbath for example).

5. Coating buffer

The coating buffer is prepared using powder-filled capsules.
One tablet in 100 ml of RO-DI water yields a 0.05 M carbonate/bicarbonate buffer, pH 9.6.

Alternative:
Stock solution A: Na_2CO_3 (0.06 M) = 0.159 g/25 ml
Stock solution B: $NaHCO_3$ (0.06 M) = 0.504 g/100 ml

10 ml A + 50 ml B + 175 ml RO-DI water.
Carefully add HCl or NaOH until pH to 9.6 is reached.
Adjust volume to 250 ml with RO-DI water

6. Neutralisation buffer (Carbonate/Bicarbonate buffer, 0.156 M, pH 10)

Stock solution A: Na_2CO_3 (0.61 M) = 6.466 g/100 ml
Stock solution B: $NaHCO_3$ (0.61 M) = 5.124 g/100 ml

72 ml A + 55 ml B + 300 ml RO-DI water.
Adjust pH to 10 by adding either HCl or NaOH.
Once the pH is set, adjust the volume to 500 ml with RO-DI water.

7. Phosphate citrate buffer

The Phosphate-citrate buffer is prepared using tablets.
1 tablet in 100 ml of RO-DI water to obtain a 0.05M Phosphate citrate buffer, pH 5.0.

8. Sulfuric acid (H_2SO_4)

The acid we use comes in cartridges. Take the contents of a cartridge and add RO-DI water up to 250 ml. This gives you 250 ml of H_2SO_4 4N.

- Recommended storage temperature and storage time for buffers and products

Table.2 shows the recommended storage time and storage temperature for the products and buffers used in the test. Please note that storage times of 3-4 months are indications.

Always check the state of your products. If you see a contamination inside a 1 month old buffer, you should discard it immediately. On the other hand, an unopened autoclaved bottle of PBS can still be used after 6-7 months. Check your Tween 20 each time you use it to see if it is still clear. Should it become cloudy, the Tween 20 should be replaced.

The storage time of Streptavidin-HRP depends on the storage temperature.

Table 2: *Recommended storage temperature and storage time.*

**Product/buffer**	**Storage T**	**Storage time**
Biotin Protein Labeling Kit	+ 4°C	See expiration date
Neutralisation buffer	+ 4°C	3-4 months
Carbonate-Bicarbonate Buffer tablets	Ambient temp	No indication
Coating buffer	+ 4°C	3-4 months
PBS tablets	Ambient temp	See expiration date
PBS buffer	+ 4°C	3-4 months
Tween 20	Ambient temp	No indication
Washing buffer	+ 4°C	No more than 1 week, but it is recommended to prepare new buffer before each test
Newborn Bovine serum	- 20°C	See expiration date
Blocking buffer	NA	NA
H2O2	+ 4°C	No indication
B158C11A10	- 20°C	6-7 months
B60H8A4 (biotinylated)	+ 4°C	3-4 months
OPD tablets	+ 4°C	See expiration date
Streptavidin-HPR	Follow manufacturer's instructions	Follow manufacturer's instructions
TCA ≥ 99.0% (titration)	+ 4°C	No indication
TCA solution	NA	NA
Phosphate citrate tablets	Ambient temp	No indication
Phosphate citrate buffer	+ 4°C	3-4 months
TITRISOL H2SO4	Ambient temp	See expiration date
H2SO4 4N solution	Ambient temp	6 months

NA = Not applicable; prepare fresh buffer/solution before each test.

1.2 Apparatus, equipment and materials

- Centrifuge for eppendorf tubes
- Micro pipettes (0.5 µl - 10 µl, 50 µl - 200 µl, 100 µl - 1000 µl) + tips (When available: an Eppendorf Multipette 4780 + 5 ml cartridge tips and/or multichannel pipettes)
- ELISA plates
- ELISA reader with 492 (and if available 655 nm) filters
- Shaker incubator (37°C)
- Incubator at 30°C
- Vortex and magnetic stirrer
- Balance (reach: 0.001 g) + spatula
- Dark recipient for making the substrate or aluminum wrapping foil.
- Eppendorf tubes (1.5 ml) + rack
- Disposable syringe of 20 ml that is filled with washing buffer, a handheld dispenser connected to a container filled with washing buffer or a fully automated ELISA washer.
- Measuring flasks and beakers
- Falcon tubes (15 hand 50 ml)

1.3 Sample for analysis

- "Pretreatment" of the samples

Aim of the pretreatment:

1. Break down immune complexes to obtain free circulating antigen.
2. Reduce cross-reactions with sera of i.a. individuals infected with Trypanosoma.

To do this, the samples are mixed with an equal volume of trichloroacetic acid (TCA).

This breaks down the immune complexes. The samples are then neutralised (= bring pH from low the neutral) with a neutralisation buffer.

- Negative control sera (only 1 well required): 75 µl TCA (5%) solution + 75 µl serum sample.

  Positive control and unknown sera (2 wells per sample): 150 µl TCA (5%) solution + 150 µl serum sample.
- Mix immediately by vortexing.

- Incubate for 20 min. at ambient temperature.
- Mix again by vortexing.
- Centrifuge for 9 min. at 12000*g*.
- While centrifuging, prepare eppendorfs with 75 µl (negative control sera) or 150 µl (positive and unknown sera) neutralisation buffer (pH 10).
- Neutralise mixture by adding 75 or 150 µl (for negative controls or other samples, respectively) of the supernatant into the eppendorfs with the same amount of neutralisation buffer. This results in a final dilution of 1/4 of your sample(s).

Important note: When using positive control sample K504, you must "pre-dilute" the sample before doing the TCA treatment.

- Add 5 µl K504 serum to 795 µl PBS
- Vortex well
- From this 800 µl you take 150 µl and add it to 150 µl of TCA

  Then you continue with a normal TCA treatment.

This gives the sample a final dilution of 1/640 as opposed to a 1/4 dilution for a normal sample.

- Controls

  Please adopt the plate layout as it is shown in the table.

  Note that positive controls (+1 & +2) and the unknown samples (? 1 to ? 40) are 2wells/ sample, while the negative controls (-1 to -8) are 1 well/sample.

Table 3: *Plate layout*

	1	2	3	4	5	6	7	8	9	10	11	12
**A**	SC	-1	? 1	? 5	? 9	? 13	? 17	? 21	? 25	? 29	? 33	? 37
**B**	SC	-2	? 1	? 5	? 9	? 13	? 17	? 21	? 25	? 29	? 33	? 37
**C**	CC	-3	? 2	? 6	? 10	? 14	? 18	? 22	? 26	? 30	? 34	? 38
**D**	CC	-4	? 2	? 6	? 10	? 14	? 18	? 22	? 26	? 30	? 34	? 38
**E**	+1	-5	? 3	? 7	? 11	? 15	? 19	? 23	? 27	? 31	? 35	? 39
**F**	+1	-6	? 3	? 7	? 11	? 15	? 19	? 23	? 27	? 31	? 35	? 39
**G**	+2	-7	? 4	? 8	? 12	? 16	? 20	? 24	? 28	? 32	? 36	? 40
**H**	+2	-8	? 4	? 8	? 12	? 16	? 20	? 24	? 28	? 32	? 36	? 40

1.4 (Bio-)Safety and environment

- Always wear gloves when handling samples, especially human samples, as there might be HIV, ... danger
- TCA and H_2SO_4 may cause severe burns, so wear protective gloves and avoid release to the environment. In case of contact with eyes, rinse immediately with plenty of water and seek medical advice.

- OPD may cause discomfort after skin contact and there is limited evidence of a carcinogenic effect, so wear protective gloves and avoid release to the environment.

2 Definitions and abbreviations

B158C11A10	first -capturing- antibody
B60H8A4	second -detecting- antibody
CC	conjugate control
CSF	cerebrospinal fluid
ELISA	enzyme linked immunosorbent assay
g	RCF - Rotational centrifugal force
M	molar
N	normal
NBCS	newborn calf serum
nm	nanometer
OD	optical density
OPD	ortho phenylenediamine
PBS	phosphate buffered saline
RO-DI water	reverse osmotic de-ionised water
SD	standard deviation
SC	substrate control
TCA	trichloroacetic acid
VHU	veterinary helmintholgy

3 Method

Check to make sure all reagents and products are prepared and/or available. If you have a lot of samples to test, start with the pretreatment of the samples (as described in section 1.3). If not, you can start with the coating of the plate and do the treatment during the incubation time of the coating and the blocking step.

Note: The below procedure should be done quickly. A multichannel pipet can be used to expedite the process.

**Coating**	• Capturing antibody (B158C11A10) (see tube for concentration) in coating buffer, @ concentration of 5 µg/ml<br>• 100µl in all wells except SC: only coating buffer	15min 37°C shaking or overnight at 4°C
**Wash 1X**	• Washing buffer (PBS -Tw20 0.05%)<br>• Individual wells are completely filled with washing buffer, but spill-over from one well into another must be avoided.	
**Blocking**	• Blocking buffer (PBS-Tw 20 0.05% + 1% NBCS)<br>• 150 µl in each well	15min 37°C, shaking
**Sample**	• Empty the plate, but do not wash<br>• 100 µl of pretreated samples in designated wells<br>• Positive controls and unknown samples: 2 wells/sample<br>• Negative controls: 1 well/sample.<br>• In SC and CC: blocking buffer	15min 37°C, shaking
**Wash 5X**	Washing buffer	
**Detecting**	• Detecting antibody (B60H8A4-biot.) (see tube for concentration) in blocking buffer, @ concentration of 1.25 µg/ml<br>• 100µl in each well except SC: only blocking buffer	15min 37°C, shaking
**Wash 5X**	Washing buffer	
**Conjugate**	• Dilute peroxidase labelled streptavidin 1/10 000 in blocking buffer<br>• 100µl in each well except SC only blocking buffer<br>• Take the OPD out of the fridge (20 minutes before use)	15min 37°C, shaking
**Wash 5X**	Washing buffer	
**Substrate**	• OPD: 1 tablet in 10ml phosphate citrate buffer .Prepare in a dark recipient (e.g. falcon tube wrapped in aluminium foil).<br>• Add the10µl H_2O_2 just before just before putting the substrate on the plate.<br>(If you need more than 10 ml of OPD, you must take 2 tablets in 20 ml and add 20 µl of H2O2 and so on...)<br>• 100 µl in each well	15 minutes 30°C; dark, not shaking
**Stopping**	• H_2SO_4 (4N)<br>• 50µl in all wells	
**Read**	492 and 655nm (The 492 nm filter is the most important one, as it is used to measure the maximum absorption. 655 nm is used to measure the background.)	

4 Quality control

There is a conjugate and substrate control on each plate (CC en SC, respectively). Non-specific reactions between the plate, coating/blocking and conjugate are intercepted by the conjugate control. The quality of the substrate (e.g.

by influence of light) can be traced by the substrate control. Both controls need to be negative (below cut-off value). The table below shows the contents of the SC and CC controls for each step of the ELISA assay.

Table 4*: SC and CC controls*

ELISA step	SC	CC
Coating	Only coating buffer	Capturing antibody
Blocking	Blocking buffer	Blocking buffer
Sample	Blocking buffer	Blocking buffer
Detecting	Blocking buffer	Detecting antibody
Conjugate	Blocking buffer	Streptavidin
Substrate	Substrate	Substrate

Negative control samples (-1 to -8) are used to calculate the cut-off of the assay and should be matched to the species of the samples to be tested, to avoid any bias in the interpretation of the results.

Positive control samples (+1 & +2) are used to see if the assay itself was successful.

If desired, you can contact the VHU for a set of positive and negative reference samples to evaluate the assay in your lab.

- Consistency between the wells

  One of the most important aspects of any assay is consistency and standardization of conditions as this will affect the reproducibility and accuracy of your results.

  Therefor (automatic) multichannel pipets and plate washers provide more consistent and faster results, as well as higher throughput. Certainly in this ELISA the use of a multichannel pipet is very important if you work with full multiwell plates because of the short incubations. Further calibrations of all pipettors on a regular base are necessary, or there can be significant variation in the results.

- Technical validation

  - The eight negative reference samples can be checked by means of the following formula:

    100 X SD/MEAN < 100

  - The unknown samples can be evaluated also with the above formula.
  - The positive reference samples are used to avoid mistakes during the test. The fact that they give a positive result suffices.

- Interpretation of the results

  - All positive and unknown samples are done in duplicate. Check if the 2 wells containing the same sample give roughly the same OD. If this is ok, you then calculate the average OD for every sample.

$$\text{average OD} = [OD_{\text{well 1}} + OD_{\text{well 2}}]/2$$

- The cut off is calculated based on the OD's of the negative samples using a variation of the students test (Sokal and Rohlf, 1981). Once the cut off has been determined, it can be used to calculated a ratio.

$$\text{Ratio} = \text{average OD/cut off}$$

- When the ratio is > 1, the sample is considered positive with a certainty of 99.9 %.
- There is a Microsoft Office Excel file available to assist with these calculations.

  Please contact the VHU for more information.

5 Reporting and Archiving

- Storage location of all raw data generated, L:Biomedical Science/Parasitology/ Vet helm/ labo/ ELISA
- Storage location of samples. -20°C Freezer VHU

6 References

- Brandt, J., Geerts, S., De Deken, R., Kumar, V., Ceulemans, F., Brijs, L., Falla, N., 1992. A monoclonal antibody-based ELISA for the detection of circulating excretory-secretory antigens in Taenia saginata cysticercosis. Int J Parasitol 22, 471-477.
- Castillo, Y., Rodrìguez, S., García, H. H., Brandt, J., Van Hul, A., Silva, M., Rodriguez-Hidalgo, R., Portocarrero, M., Melendez, D. P., Gonzalez, A. E., Gilman, R. H., Dorny, P., 2009. Urine antigen detection for the diagnosis of human neurocysticercosis. Am.J.Trop.Med.Hyg. 80 (3), 379-383.
- Choromanski, L., Estrada, J. J., Kuhn, R. E., 1990. Detection of antigens of larval Taenia solium in the cerebrospinal fluid of patients with the use of HPLC and ELISA. J Parasitol 76 (1), 69-73.
- Correa, D., Sandoval, M. A., Harrison, L. J., Parkhouse, R. M. E., Plancarte, A., Meza-Lucas, A., Flisser, A., 1989. Human neurocysticercosis: comparison of enzyme immunoassay capture techniques based on monoclonal and polyclonal antibodies for the detection of parasite products in cerebrospinal fluid. Trans.R.Soc.Trop.Med.Hyg. 83 (6), 814-816.
- Dorny, P., Brandt, J., Geerts, S., 2004. Immunodiagnostic approaches for detecting Taenia solium. Trends Parasitol. 20 (6), 259-260.
- Dorny, P., Holland, W., My, L. N., Erhart, A., De, N. V., Cong, L. D., Geerts, S., Brandt, J., Vercruysse, J., 2001. Taenia solium cysticercosis in Vietnam. Proceedings of the 18th International Conference of the World Association for the Advancement of Veterinary Parasitology, Stresa, Italy, 26 - 30 August.

- Dorny, P., Vercammen, F., Brandt, J., Vansteenkiste, W., Berkvens, D., Geerts, S., 2000. Sero-epidemiological study of Taenia saginata cysticercosis in Belgian cattle. Vet Parasitol 88 (1-2), 43-49.
- Erhart, A., Dorny, P., van De, N., Vien, H. V., Thach, D. C., Toan, N. D., Cong, L. D., Geerts, S., Speybroeck, N., Berkvens, D., Brandt, J., 2002. Taenia solium cysticercosis in a village in northern Viet Nam: seroprevalence study using an ELISA for detecting circulating antigen. Trans.R.Soc.Trop.Med.Hyg. 96 (3), 270-272.
- Fall, E. H., Kumar, V., Geerts, S., Salvoldi, M., Kama, M., 1996. Comparison of single and trickle infections of pigs with eggs of the Asian Taenia. Vet Parasitol 61 (3-4), 231-237.
- Garcìa, H. H., Harrison, L. J., Parkhouse, R. M. E., Montenegro, T., Martinez, S. M., Tsang, V. C., Gilman, R. H., 1998. A specific antigen-detection ELISA for the diagnosis of human neurocysticercosis. The Cysticercosis Working Group in Peru. Trans.R.Soc.Trop.Med.Hyg. 92 (4), 411-414.
- Garcìa, H. H., Parkhouse, R. M. E., Gilman, R. H., Montenegro, T., Bernal, T., Martinez, S. M., Gonzalez, A. E., Tsang, V. C., Harrison, L. J., 2000. Serum antigen detection in the diagnosis, treatment, and follow-up of neurocysticercosis patients. Trans.R.Soc.Trop.Med.Hyg. 94 (6), 673-676.
- Geerts, S., Zorloni, A., Kumar, V., Brandt, J., De Deken, R., Eom, K. S., 1992. Experimental infection of pigs with a Taenia species from Korea: parasitological and serological aspects. Parasitol Res 78 (6), 513-515.
- Harlow, E., Lane, D., 1988. Antibodies, A Laboratory Manual. Cold Spring Harbor Laboratory.
- Harrison, L. J., Joshua, G. W., Wright, S. H., Parkhouse, R. M. E., 1989. Specific detection of circulating surface/secreted glycoproteins of viable cysticerci in Taenia saginata cysticercosis. Parasite Immunol 11 (4), 351-370.
- Rajshekhar, V., Joshi, D. D., Doanh, N. Q., van De, N., Xiaonong, Z., 2003. Taenia solium taeniosis/cysticercosis in Asia: epidemiology, impact and issues. Acta Trop 87 (1), 53-60.
- Sokal, R. R., Rohlf, F. J., 1981. Biometry, 2nd Edition. Freedman and Company, New
- York.
- Van Kerckhoven, I., Vansteenkiste, W., Claes, M., Geerts, S., Brandt, J., 1998. Improved detection of circulating antigen in cattle infected with Taenia saginata metacestodes. Vet Parasitol 76 (4), 269-274.
- Wang, C. Y., Zhang, H. H., Ge, L. Y., 1992. A MAb-based ELISA for detecting circulating antigen in CSF of patients with neurocysticercosis. Hybridoma 11 (6), 825-827.

7 Revision

Revision	Version number: …..
Changes with respect to the previous published version:	New format of WEBISO

8 Approval

	Name and function
**Created by (First Version)**	Anke Van Hul, lab technician
**Updated by (Current Version):**	ELISA cysticercosis protocol.v1.5
**Approved by:**	Pierre Dorny, Unit Head
