# Supplementary material for: One Health in Action: Operational Aspects of an Integrated Surveillance System for Zoonoses in Western Kenya
Source: Front Vet Sci. 2019 Jul 31;6:252. doi: 10.3389/fvets.2019.00252 (PMC6684786; doi:10.3389/fvets.2019.00252)
Supplement: Supplementary file 14 [file Table_14.docx]

**
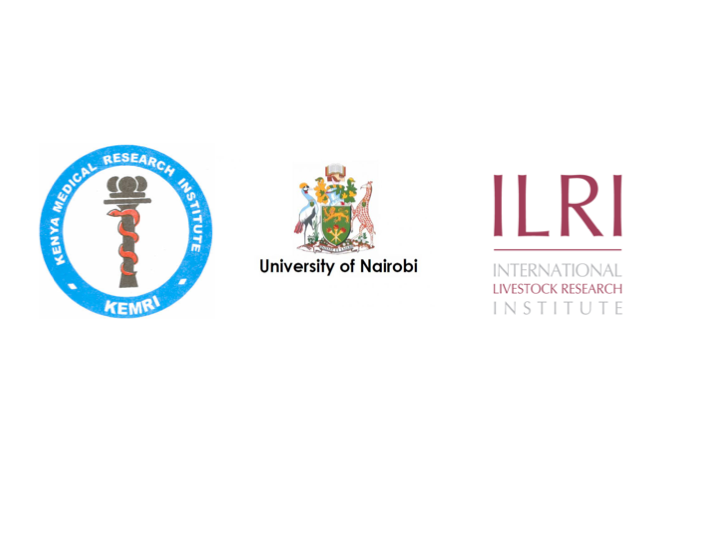
**

| **SOP NO:** **ZOOLINK/BUSIA/TPM/2017** | **Version: Original** | **Effective date: 1/4/2017** |
| --- | --- | --- |
| **Title: Trypanosome microscopy – ZooLink project** | | |
| **Prepared by: Sam Njoroge** | **Sign:** 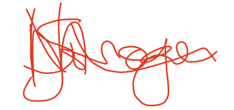 | **Date:21-Feb-2017** |

1. **PURPOSE / INTRODUCTION:**

Animals and humans maybe infected with trypanosomes.

The aim of the ZooLink project is to screen for trypanosomes in animals and humans from EDTA blood samples collected from Busia, Bungoma and Kakamega Counties using microscopy.

This SOP describes means and methods needed for the screening of trypanosomes using microscopy.

1. **SCOPE / RESPONSIBILITY:**

This SOP applies to all personnel and persons on attachment who are involved in trypanosome microscopy in the ZooLink project. The section head must ensure that the procedure is strictly followed.

The QA officer should coordinate and supervise the process to ensure all the SOPs are current and up to date.

The technical personnel should prepare, review and update the SOPs related to their work and occasional training for both new and old technical personnel to which the SOP apply.

1. **SAFETY/RISK ASSESSMENT**:

Biosafety Level 2 practices should be observed when handling sera. Carry out all procedures in accordance with local safety codes of practice.

All blood samples are potentially contagious. Respect the universal precautions.

USE SINGLE- USE GLOVES DURING THE ENTIRE PROCEDURE!

• Giemsa is inflammable; manipulate this product far from any flame.

1. **EQUIPMENT / MATERIALS/ REAGENTS:**

• Single-use, non-sterile gloves

• New microscopic glass slides

• Concentrated Giemsa (Merck N° 1.09204)

• pH 7.2 tablets (Merck N° 9468)

• pH-meter and calibration solution

• Bottle of 1000 ml

• Clean water or tap water

• Dropper bottle

• Pasteur pipettes

• 10 ml graduated cylinder

• 200 ml beaker

• Support for staining

• Rack for slides

• Timer

• Microscope (objective 100 x)

• Immersion oil

• 2 touch counters

• Storage box for slides

• Tissue

**5.0 Reagent**

- • Giemsa stain.

**6.0 Samples**

- •Thin smears from EDTA blood

1. **METHODOLOGY:**

Method This method applies to both thin blood films and tissue films

a. Fix in methanol for 2 minute

b. Stain with Giemsa 1 in 10 in buffered distilled water pH 6.8 for 30

c. Wash the slide in running water and drain dry

Trypomastigotes of Trypanosoma species is an elongated cell with single nucleus which usually lies near the centre of the cell. Each cell bears a single flagellum which appears to arise from a small granule - the kinetoplast. The length and position of the trypanosome’s flagellum is variable. In trypanosomes from the blood of a host the flagellum originates near the posterior end of the cell and passes forward over the cell surface, its sheath is expanded and forms a wavy flange called an undulating membrane.

**DOCUMENT CHANGE HISTORY:**

**Version Table:**

| Original:  Title: | Dated:  **1/4/2017** | SOP No.:  **ZOOLINK/BUSIA/TPM/2017** | No. Pages:  **4** |
| --- | --- | --- | --- |
| Version:  Title: | Dated: | SOP No.: | No. Pages: |
| Version:  Title: | Dated: | SOP No.: | No. Pages: |

**Training Documentation Log for SOP Files**

| Kenya Medical Research Institute  **ZOOLINK/BUSIA/** SOP | | |  | SOP No: **ZOOLINK/BUSIA/TPM/2017**  Version: **Original**  Effective Date: **1/4/2017** | | |
| --- | --- | --- | --- | --- | --- | --- |
| Trypanosome microscopy – ZooLink project | | | | | | |
| **NO.** | **DATE** | **NAME** | | | **SIGNATURE** | **TRAINER** |
|  |  |  | | |  |  |
|  |  |  | | |  |  |
|  |  |  | | |  |  |
|  |  |  | | |  |  |
|  |  |  | | |  |  |
|  |  |  | | |  |  |
|  |  |  | | |  |  |
|  |  |  | | |  |  |
|  |  |  | | |  |  |
|  |  |  | | |  |  |
|  |  |  | | |  |  |
|  |  |  | | |  |  |
|  |  |  | | |  |  |
|  |  |  | | |  |  |
