# Supplementary material for: One Health in Action: Operational Aspects of an Integrated Surveillance System for Zoonoses in Western Kenya
Source: Front Vet Sci. 2019 Jul 31;6:252. doi: 10.3389/fvets.2019.00252 (PMC6684786; doi:10.3389/fvets.2019.00252)
Supplement: Supplementary file 15 [file Table_15.docx]

**
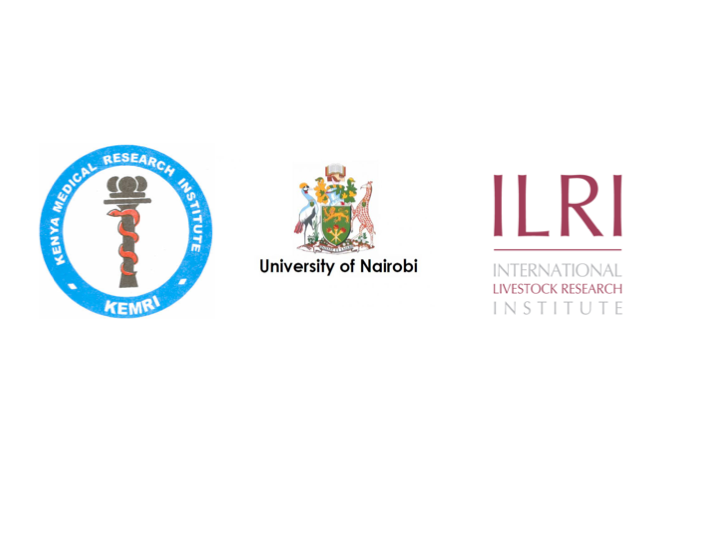
**

| **SOP NO:** **ZOOLINK/BUSIA/ATX/2017** | **Version: Original** | **Effective date: 1/4/2017** |
| --- | --- | --- |
| **Title: Anthrax microscopy – ZooLink project** | | |
| **Prepared by: Sam Njoroge** | **Sign:** 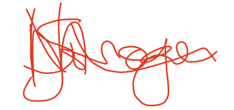 | **Date:21-Feb-2017** |

1. **PURPOSE / INTRODUCTION:**

Animals maybe infected with anthrax.

The aim of the ZooLink project is to screen for anthrax in dead animals, and to assess anthrax as a possible cause of sudden death in Busia, Bungoma and Kakamega Counties using microscopy.

This SOP describes means and methods needed for the screening of anthrax using microscopy.

Polychrome methylene blue is a complex mixture of methylene blue and substantial amounts of other homologs, primarily azure A and azure B, which are produced by oxidation ("ripening") which takes place in methylene blue solution upon standing. Natural ripening takes a year or more to complete but can be hastened by addition of 1% K2CO3 to Loeffler's alkaline methylene blue.

1. **SCOPE / RESPONSIBILITY:**

This SOP applies to all personnel and persons on attachment who are involved the microscopy of ZooLink project. The section head must ensure that the procedure is strictly followed.

The QA officer should coordinate and supervise the process to ensure all the SOPs are current and up to date.

The technical personnel should prepare, review and update the SOPs related to their work and occasional training for both new and old technical personnel to which the SOP apply.

1. **SAFETY/RISK ASSESSMENT**:

Biosafety Level 3 practices should be observed when handling sera. Carry out all procedures in accordance with local safety codes of practice

1. **EQUIPMENT / MATERIALS/ REAGENTS:**

- • Microscope
- • glass slides
- • disposable pipette tips
- • small biohazard bags

**5.0 Reagent**

- • Methylene blue stain.

**6.0 Samples**

- •tissue smears from dead animals

1. **METHODOLOGY:**

**Polychrome methylene blue staining procedure**

1. make a thin smear of blood, tissue fluid, etc. by spreading a small (1-5 µl) drop on a microscope slide with the edge of a coverslip (remember this is potentially infective material; discard the coverslip into hypochlorite solution). Air dry and fix by dipping in absolute, or 95% methanol or ethanol for 30-60 seconds and re-drying
2. put a large drop (approx. 20 µl) of polychrome methylene blue on the smear, spreading with an inoculating loop to cover all parts of the smear. Leave for 30-60 seconds ("flooding the slide" with the stain is unnecessary and wasteful)
3. wash the stain off with water into hypochlorite solution. Blot, dry and examine. The bacteria can be seen as fine threads of varying length under the low power (10x) objective. Under oil immersion (100x objective), the capsule is seen clearly (pink) surrounding the blue-black, often square-ended bacilli. (Discard the blotting paper and slide into containers destined for autoclaving or into hypochlorite solution when finished with).

Cautions on negatives

Very little is known about the development and changing nature of the capsule with time, but it has been noted by Turnbull and Lindeque (unpublished), and in occasional other personal communications, that commercial polychrome methylene blue may fail to reveal the capsule in specimens from anthrax victims in the field while being able to do so with artificially induced capsules obtained by in vitro growth of the organisms in blood or on bicarbonate agar (see A.I.2.2 below). It is suspected that this is related to inadequate maturation of the stain. The consequences are considerable, however, and laboratories are urged to ensure new stocks are thoroughly tested for reliability before old stocks are finished and discarded.


**DOCUMENT CHANGE HISTORY:**

**Version Table:**

| Original:  Title: | Dated:  **1/4/2017** | SOP No.:  **ZOOLINK/BUSIA/ATX/2017** | No. Pages:  **3** |
| --- | --- | --- | --- |
| Version:  Title: | Dated: | SOP No.: | No. Pages: |
| Version:  Title: | Dated: | SOP No.: | No. Pages: |

**Training Documentation Log for SOP Files**

| Kenya Medical Research Institute  **ZOOLINK/BUSIA/** SOP | | |  | SOP No: **ZOOLINK/BUSIA/ATX/2017**  Version: **Original**  Effective Date: **1/4/2017** | | |
| --- | --- | --- | --- | --- | --- | --- |
| Anthrax microscopy – ZooLink project | | | | | | |
| **NO.** | **DATE** | **NAME** | | | **SIGNATURE** | **TRAINER** |
|  |  |  | | |  |  |
|  |  |  | | |  |  |
|  |  |  | | |  |  |
|  |  |  | | |  |  |
|  |  |  | | |  |  |
|  |  |  | | |  |  |
|  |  |  | | |  |  |
|  |  |  | | |  |  |
|  |  |  | | |  |  |
|  |  |  | | |  |  |
|  |  |  | | |  |  |
|  |  |  | | |  |  |
|  |  |  | | |  |  |
|  |  |  | | |  |  |
